# Synthesis of 2*H*-Chromenones from Salicylaldehydes and Arylacetonitriles

**DOI:** 10.3390/molecules22071197

**Published:** 2017-07-18

**Authors:** Chengcai Li, Hailin Zhu, Hang Zhang, Yongfeng Yang, Feng Wang

**Affiliations:** 1 Zhejiang Provincial Key Laboratory of Fiber Materials and Manufacturing Technology, Zhejiang Sci-Tech University, Xiasha Campous, Hangzhou 310018, China; lcc692716700@163.com (C.L.); m15988811917@163.com (H.Z.); 13735539235@163.com (Y.Y.); membr_wang@163.com (F.W.); 2 Zhejiang Kertice Hi-Tech Fluor-Material Co., LTD, Huzhou 313000, China

**Keywords:** chromenones synthesis, metal-free, green chemistry, heterocycle synthesis, salicylaldehydes

## Abstract

An efficient and convenient protocol for the synthesis of 2*H*-chromenones has been developed. In the presence of *^t^*BuOK in DMF, good to excellent yields of various chromenones were obtained from the corresponding salicylaldehydes and arylacetonitriles. No protection of inert gas atmosphere is required here.

## 1. Introduction

**Figure 1:**
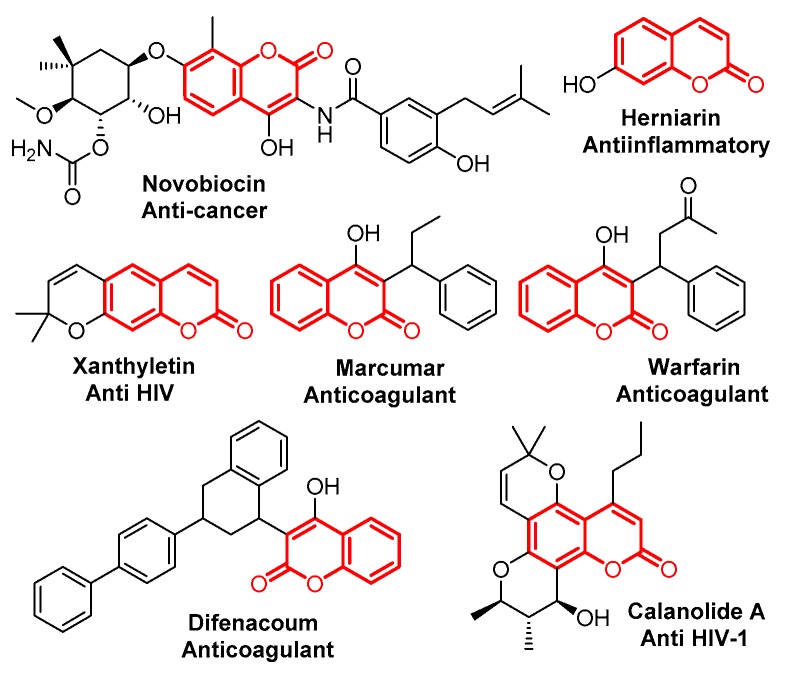
Selected examples of bioactive chromenones.

Coumarin is an important class of benzo-fused six-membered heterocycles, which was first isolated as a natural product in 1820, and has been found to have various interesting bioactivities (Figure 1) [1,2,3,4,5,6,7,8]. Due to its importance, many efforts have been made to develop new synthetic procedures for coumarin’s preparation. Classical routes to coumarins based on Pechmann- [9], Knoevenagel- [10], Perkin- [11], Reformatsky- [12] and Wittig- [13] reactions have been extensively investigated. Recently, procedures based on transition metal catalysts, ionic liquids and microwaves have been developed as well [14,15,16,17].

In the 21st century, the demands of sustainable development drive organic chemists to pay more attention to the principles of green chemistry in designing their new procedures. Among the various possible directions, the development of new transition metal-free methodologies will be one attractive choice. On one hand, transition metal catalysts are usually considered to be toxic and non-environmentally benign. On the other hand, special attention has to be taken to avoid the problem of transition metal contamination of the final products, especially when in heterocycles synthesis chemistry. With these points in mind and also based on our continual interests in the development of new procedures for the synthesis of heterocycles under transition metal-free conditions [18], we wish to report here a convenient methodology for the construction of coumarins from salicylaldehydes and arylacetonitriles. In the presence of *^t^*BuOK in DMF, good to excellent yields of the desired chromenones were obtained and no protection of inert gas atmosphere is required here.

## 2. Results and Discussion

Initially, we choose salicylaldehyde and 2-phenylacetonitrile as the model substrates to establish this reaction system (Table 1). As we expected, with two equivalents of *^t^*BuOK as the base in 2 mL of dimethylformamide (DMF) at 110 °C, 77% of the desired product can be isolated (Table 1, entry 1). No better results can be obtained with an increased amount of promotor and similar yield can be observed with a higher concentration (Table 1, entries 2 and 3). Then, several other inorganic bases were screened and none of them could give better results than *^t^*BuOK (Table 1, entries 5–10). The reaction temperature was also checked and yields were reduced when the reaction temperature was decreased or increased (Table 1, entries 11 and 12). Subsequently, various solvents were examined but without improved results (Table 1, entries 13–17).

With the optimal reaction conditions in hand, several substituted salicylaldehydes were tested and shown in Table 2. Moderate to good yields of 2*H*-chromenones can be obtained from the corresponding salicylaldehydes and 2-phenylacetonitrile.

Then, various arylacetonitriles were examined with salicylaldehyde (Table 3). Both electron-donating and electron-withdrawing substituted phenylacetonitriles afforded the corresponding products in moderate to good yields. Notably, when 2-(2-fluorophenyl)acetonitrile and 2-(2-chlorophenyl)acetonitrile were applied as the reaction partner, good yields of dibenzo(*b,f*)oxepine-10-carbonitrile can be obtained via intermolecular condensation and intramolecular nucleophilic substitution (Table 3, entries 6 and 9) [19]. It is also important to mention that 3-oxo-3-phenylpropanenitrile, 3-phenylpropanenitrile and malononitrile were tested under standard conditions but no desired products can be detected (Table 3, entries 14–16).

**Scheme 1:**
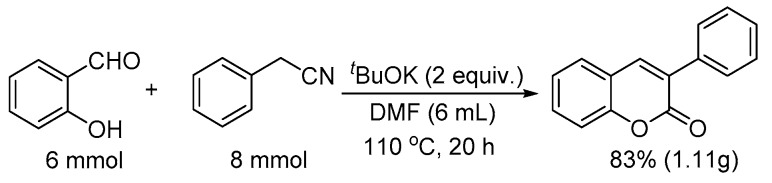
Gram scale synthesis of 3-phenyl-2*H*-chromen-2-one.

To demonstrate the potential utility of this method, we conducted the reaction in gram scale as well (Scheme 1). Thus, salicylaldehyde (6 mmol) was reacted with phenylacetonitrile (8 mmol) in the presence of two equivalents; for *^t^*BuOK at 110 °C for 20 h, 83% yield of 3-phenyl-2*H*-chromen-2-one was obtained (1.11 g).

**Scheme 2:**
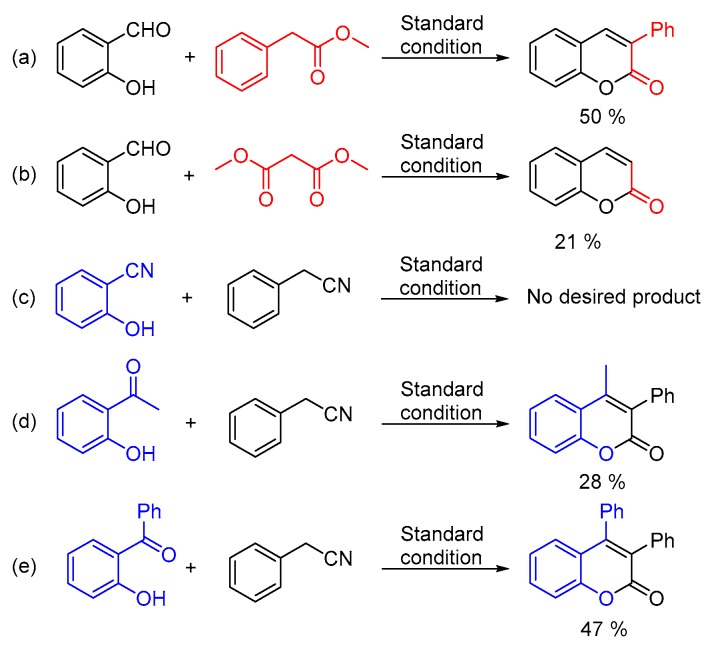
Substrate analogues testing. (**a**) methyl 2-phenylacetate (**b**) dimethyl malonate (**c**) 2-hydroxybenzonitrile (**d**) 2-acetylphenol (**e**) 2-hydroxybenzophenone.

In addition, analogues of the substrates have been tested as well (Scheme 2). Under the standard conditions, 50% of 3-phenyl-2*H*-chromen-2-one and 21% of 2*H*-chromen-2-one could be obtained from methyl 2-phenylacetate and dimethyl malonate, respectively (Scheme 2, equation. a and equation. b). Moreover, 2-hydroxybenzonitrile, 2-acetylphenol and 2-hydroxybenzophenone were taken into consideration as well. Unfortunately, no desired product could be detected from 2-hydroxybenzonitrile (Scheme 2, equation. c). Interestingly, 2-acetylphenol could afford acceptable yield of the goal product (Scheme 2, equation. d) and moderate yield of 3,4-diphenyl-2*H*-chromen-2-one was generated from 2-hydroxybenzophenone and phenylacetonitrile without any further optimization (Scheme 2, equation. e) [20].

**Scheme 3:**
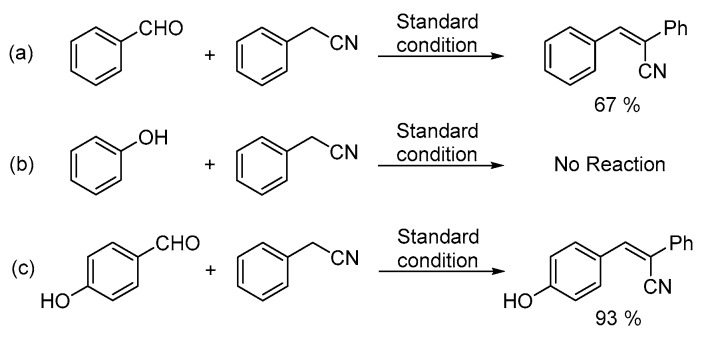
Control experiments. (**a**) Benzaldehyde (**b**) phenol (**c**) 4-hydroxybenzaldehyde.

In order to obtain more insight into the reaction pathway, control experiments were performed (Scheme 3). Benzaldehyde, phenol and 4-hydroxybenzaldehyde were reacted with phenylacetonitrile under the standard reaction conditions, respectively. When benzaldehyde was reacted with phenylacetonitrile, 67% of 2,3-diphenylacrylonitrile was obtained while no product could be detected with phenol (Scheme 3, equation. a and equation. b). Compared with salicylaldehyde, 4-hydroxybenzaldehyde is considered as a compound with the same electron properties. Under the same reaction conditions, 93% of 3-(4-hydroxyphenyl)-2-phenylacrylonitrile was generated as the sole product by the reaction of 4-hydroxybenzaldehyde with phenylacetonitrile (Scheme 3, equation. c), which indicated that the first step of this transformation is the intermolecular condensation instead of the nucleophilic addition [21].

**Scheme 4:**
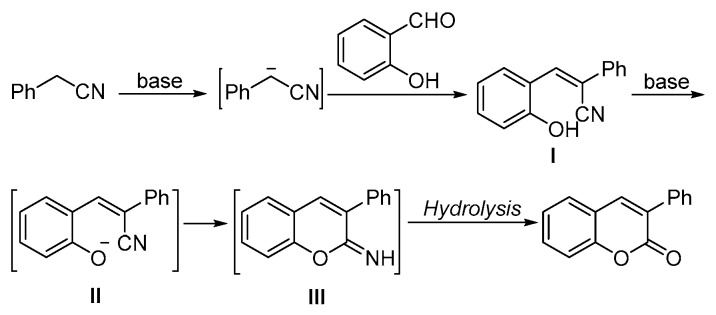
Proposed mechanism of the synthesis of chromenone.

Based on these results, a possible reaction pathway has been proposed (Scheme 4). In the presence of base, phenylacetonitrile transformed into cyano(phenyl)methanide, which subsequently reacted with salicylaldehyde to give intermediate **I**. With the assistance of the other equivalent base, the hydroxyl group of intermediate **I** was activated and then reacted with the cyano via an intramolecular addition. The final products will be formed after in situ hydrolysis.

## 3. Materials and Methods 

### 3.1. Materials and General Procedures

#### 3.1.1. Materials

General comments: All reactions were carried out under air. Reactions were monitored by TLC analysis (pre-coated silica gel plates with fluorescent indicator UV254, 0.2 mm) and visualized with 254 nm UV light. Chemicals were purchased from Aldrich (Tianjin, China), Alfa-Aesar (Tianjin, China), TCI (Shanghai, China) and unless otherwise noted were used without further purification. All compounds were characterized by ^1^H-NMR and ^13^C-NMR spectroscopy and recorded on Bruker (Beijing, China) AV 300 and AV 400 spectrometers. Gas-chromatography-mass-analysis was performed using an Agilent HP-5890 with an Agilent HP-5973 Mass Selective Detector (EI) and an HP-5-capillary column using helium as a carrier gas.

#### 3.1.2. General Procedures

Salicylaldehyde (1 mmol) and two equivalents of *^t^*BuOK were added in a 25 mL tube equipped with a stirring bar. Then, 1 mL of DMF and 2-phenylacetonitrile (1.5 mmol) were injected by syringe. After that, the tube was closed and heated up to 110 °C for 16 h. When the reaction was completed, the reaction mixture was cooled to room temperature. The reaction was quenched with distilled water and the solution was extracted with ethyl acetate. The crude product was purified by column chromatography (ethyl acetate/pentane = 1:25–1:8).

### 3.2. Synthesis of Adducts (Specific Spectral Reference Supplementary Materials)

*3-Phenyl-2H-chromen-2-one*: ^7t1^H-NMR (300 MHz, Chloroform-*d*) δ 7.74 (d, *J* = 0.6 Hz, 1H), 7.66–7.60 (m, 2H), 7.50–7.41 (m, 2H), 7.41–7.32 (m, 3H), 7.29 (dq, *J* = 7.7, 0.9 Hz, 1H), 7.25–7.19 (m, 1H). ^13^C-NMR (75 MHz, Chloroform-*d*) δ 160.55, 153.48, 139.83, 134.67, 131.36, 128.83, 128.49, 128.44, 128.33, 127.87, 124.46, 119.64, 116.42. GC-MS (EI, 70 ev): *m*/*z* (%) = 222 (M^+^, 100), 195 (14), 194 (93), 166 (12), 165 (89), 164 (16), 163 (10), 82 (11).

*6-Methyl-3-phenyl-2H-chromen-2-one*: ^7u1^H-NMR (300 MHz, Chloroform-*d*) δ 7.70 (s, 1H), 7.67–7.58 (m, 2H), 7.48–7.33 (m, 3H), 7.31–7.24 (m, 2H), 7.23–7.14 (m, 1H), 2.36 (s, 3H). ^13^C-NMR (75 MHz, Chloroform-*d*) δ 160.74, 151.61, 139.84, 134.81, 134.11, 132.40, 128.71, 128.48, 128.40, 128.14, 127.65, 119.36, 116.11, 20.76. GC-MS (EI, 70 ev): *m*/*z* (%) = 236 (M^+^, 100), 209 (10), 208 (67), 207 (62), 179 (24), 178 (40), 152 (16), 139 (10), 89 (12), 77 (13), 76 (12), 51 (11).

*6-Fluoro-3-phenyl-2H-chromen-2-one*: ^1^H-NMR (300 MHz, Chloroform-*d*) δ 7.75 (s, 1H), 7.73–7.67 (m, 2H), 7.50–7.41 (m, 3H), 7.35 (dddd, *J* = 8.8, 4.5, 1.8, 1.1 Hz, 1H), 7.29–7.19 (m, 2H). ^13^C-NMR (75 MHz, Chloroform-*d*) δ 160.17, 149.63, 138.73, 134.28, 129.52, 129.16, 128.54, 128.53, 120.28, 118.76 (d, *J* = 24.6 Hz), 118.05, 117.94, 113.05 (d, *J* = 23.9 Hz). GC-MS (EI, 70 ev): *m*/*z* (%) = 240 (M^+^, 94), 213 (15), 212 (96), 184 (15), 183 (100), 182 (12), 181 (10), 163 (11), 157 (13), 91 (10). HRMS (EI): Calcd. for [[M + H]^+^: C_15_H_9_FO_2_]^+^: 241.06593, found: 241.06566.

*6-Chloro-3-phenyl-2H-chromen-2-one*: ^7t1^H-NMR (300 MHz, Chloroform-*d*) δ 7.73 (t, *J* = 0.5 Hz, 1H), 7.72–7.66 (m, 2H), 7.53 (d, *J* = 2.4 Hz, 1H), 7.50–7.41 (m, 4H), 7.31 (dt, *J* = 8.8, 0.6 Hz, 1H). ^13^C-NMR (75 MHz, Chloroform-*d*) δ 160.02, 151.88, 138.45, 134.25, 131.31, 129.75, 128.69–128.43 (m), 129.56, 129.25, 128.58, 127.10, 120.73, 117.93. GC-MS (EI, 70 ev): *m*/*z* (%) = 256 (M^+^, 100), 230 (30), 229 (15), 166 (10), 165 (77), 164 (20), 163 (28), 139 (18), 82 (18), 63 (15).

*Methyl-2-oxo-3-phenyl-2H-chromene-6-carboxylate*: ^7t1^H-NMR (300 MHz, Chloroform-*d*) δ 8.28 (d, *J* = 2.0 Hz, 1H), 8.19 (dd, *J* = 8.7, 2.0 Hz, 1H), 7.89–7.84 (m, 1H), 7.76–7.65 (m, 2H), 7.55–7.34 (m, 5H), 3.96 (s, 3H). ^13^C-NMR (75 MHz, Chloroform-*d*) δ 165.70, 159.85, 156.21, 139.23, 134.17, 132.28, 129.94, 129.19, 128.56, 128.50, 128.35, 126.59, 119.39, 116.65, 52.47. GC-MS (EI, 70 ev): *m*/*z* (%) = 280 (M^+^, 100), 252 (11), 249 (30), 221 (45), 193 (29), 165 (27), 164 (12), 163 (14), 139 (22), 83 (15).

*8-Methyl-3-phenyl-2H-chromen-2-one*: ^7t1^H-NMR (300 MHz, Chloroform-*d*) δ 7.79 (s, 1H), 7.75–7.64 (m, 2H), 7.51–7.27 (m, 5H), 7.19 (dd, *J* = 8.1, 7.0 Hz, 1H), 2.49 (s, 3H). ^13^C-NMR (75 MHz, Chloroform-*d*) δ 160.59, 151.78, 140.19, 134.76, 132.61, 129.02, 128.64, 128.43, 128.35, 127.80, 125.78, 125.56, 123.97, 119.29, 15.38. GC-MS (EI, 70 ev): *m*/*z* (%) = 236 (M^+^, 100), 209 (12), 208 (76), 207 (45), 179 (19), 178 (36), 165 (30), 152 (12), 89 (14), 77 (10), 76 (12). 

*6,8-Dichloro-3-phenyl-2H-chromen-2-one*: ^7v1^H-NMR (300 MHz, Chloroform-*d*) δ 7.75–7.63 (m, 3H), 7.57 (d, *J* = 2.3 Hz, 1H), 7.48–7.39 (m, 4H). ^13^C-NMR (75 MHz, Chloroform-*d*) δ 158.81, 147.77, 137.89, 133.72, 131.20, 130.23, 129.50, 129.48, 128.60, 128.51, 125.61, 122.27, 121.44. GC-MS (EI, 70 ev): *m*/*z* (%) = 291 (M^+^, 63), 290 (94), 266 (11), 265 (10), 264 (65), 263 (16), 262 (100), 201 (20), 200 (10), 199 (62), 164 (28), 163 (60), 162 (10), 139 (10), 99 (16), 87 (11), 81 (19), 63 (10).

*7-Chloro-3-phenyl-2H-chromen-2-one*: ^1^H-NMR (300 MHz, Chloroform-*d*) δ 7.66 (s, 1H), 7.61–7.52 (m, 2H), 7.44–7.30 (m, 4H), 7.28–7.24 (m, 1H), 7.21–7.09 (m, 1H). ^13^C-NMR (75 MHz, Chloroform-*d*) δ 159.84, 153.66, 138.90, 137.23, 134.30, 129.03, 128.64, 128.49, 128.44, 128.26, 125.08, 124.91, 118.21, 116.73. GC-MS (EI, 70 ev): *m*/*z* (%) = 256 (M^+^, 100), 230 (16), 228 (100), 166 (12), 165 (85), 164 (27), 163 (28), 139 (16), 115 (14), 114 (12), 82 (11), 63 (15). HRMS (EI): Calcd. for [[M + H]^+^: C_15_H_9_ClO_2_]^+^: 257.03638, found: 257.03614.

*2-Phenyl-3H-benzo[f]chromen-3-one*: ^7u1^H-NMR (300 MHz, Chloroform-*d*) δ 8.36 (d, *J* = 1.7 Hz, 1H), 8.09 (d, *J* = 8.4 Hz, 1H), 7.83–7.68 (m, 2H), 7.67–7.59 (m, 2H), 7.50 (ddd, *J* = 8.4, 7.0, 1.4 Hz, 1H), 7.42–7.26 (m, 5H). ^13^C-NMR (75 MHz, Chloroform-*d*) δ 160.55, 153.04, 135.60, 135.00, 132.62, 130.23, 129.01, 128.80, 128.50 (d, *J* = 2.1 Hz), 128.12, 127.10, 125.96, 121.34, 116.58, 113.65. GC-MS (EI, 70 ev): *m*/*z* (%) = 272 (M^+^, 92), 245 (23), 244 (100), 243 (23), 215 (60), 213 (27), 189 (10), 122 (10), 107 (25), 94 (18).

*3-(o-Tolyl)-2H-chromen-2-one*: ^7x1^H-NMR (300 MHz, Chloroform-*d*) δ 7.65 (s, 1H), 7.60–7.46 (m, 2H), 7.39 (ddt, *J* = 8.2, 1.2, 0.6 Hz, 1H), 7.36–7.28 (m, 3H), 7.28–7.22 (m, 2H). ^13^C-NMR (75 MHz, Chloroform-*d*) δ 160.21, 153.80, 141.59, 136.82, 134.66, 131.41, 130.30, 129.73, 128.81, 127.81, 125.85, 124.43, 119.28, 116.55, 19.92. GC-MS (EI, 70 ev): *m*/*z* (%) = 236 (M^+^, 100), 220 (12), 219 (64), 208 (37), 207 (86), 189 (27), 179 (26), 178 (53), 177 (10), 176 (11), 165 (24), 152 (21), 117 (12), 115 (23), 89 (18), 76 (14), 63 (18), 39 (11).

*3-(m-Tolyl)-2H-chromen-2-one*: ^7t1^H-NMR (300 MHz, Chloroform-*d*) δ 7.76 (s, 1H), 7.55–7.43 (m, 4H), 7.37–7.22 (m, 3H), 7.22–7.16 (m, 1H), 2.39 (s, 3H). ^13^C-NMR (75 MHz, Chloroform-*d*) δ 160.55, 153.42, 139.70, 138.03, 134.59, 131.24, 129.59, 129.09, 128.44, 128.32, 127.81, 125.60, 124.40, 119.65, 116.36, 21.45. GC-MS (EI, 70 ev): *m*/*z* (%) = 236 (M^+^, 100), 209 (14), 208 (81), 207 (18), 179 (14), 178 (30), 165 (38), 152 (13), 117 (11), 89 (13), 63 (10).

*3-(p-Tolyl)-2H-chromen-2-one*: ^7t1^H-NMR (300 MHz, Chloroform-*d*) δ 7.67 (s, 1H), 7.55–7.47 (m, 2H), 7.45–7.35 (m, 2H), 7.25 (dt, *J* = 7.8, 0.9 Hz, 1H), 7.21–7.09 (m, 3H), 2.29 (s, 3H). ^13^C-NMR (75 MHz, Chloroform-*d*) δ 160.60, 153.32, 139.12, 138.82, 131.71, 131.10, 129.09, 128.31, 128.17, 127.74, 124.36, 119.68, 116.30, 21.23. GC-MS (EI, 70 ev): *m*/*z* (%) = 236 (M^+^, 100), 209 (10), 208 (62), 207 (37), 179 (13), 178 (28), 165 (26), 152 (12), 89 (11), 63 (10), 114 (12), 82 (11), 63 (15). 

*3-(Naphthalen-1-yl)-2H-chromen-2-one*: ^7t1^H-NMR (300 MHz, Chloroform-*d*) δ 7.97–7.87 (m, 2H), 7.84–7.76 (m, 2H), 7.64–7.41 (m, 7H), 7.38–7.30 (m, 1H). ^13^C-NMR (75 MHz, Chloroform-*d*) δ 160.77, 153.97, 142.77, 133.66, 132.64, 131.65, 131.53, 129.36, 128.53, 128.37, 127.93, 127.63, 126.48, 126.07, 125.23, 124.54, 119.32, 116.68. GC-MS (EI, 70 ev): *m*/*z* (%) = 272 (M^+^, 100), 273 (19), 271 (79), 255 (11), 244 (24), 243 (50), 216 (11), 215 (58), 214 (10), 213 (28), 189 (17), 107 (18), 95 (17), 63 (11).

*3-(4-Methoxyphenyl)-2H-chromen-2-one*: ^7t1^H-NMR (300 MHz, Chloroform-*d*) δ 7.75 (s, 1H), 7.71–7.63 (m, 2H), 7.56–7.44 (m, 2H), 7.34 (ddd, *J* = 8.0, 1.3, 0.7 Hz, 1H), 7.31–7.23 (m, 1H), 7.02–6.90 (m, 2H), 3.85 (s, 3H). ^13^C-NMR (75 MHz, Chloroform-*d*) δ 160.74, 160.10, 153.24, 138.43, 130.95, 129.78, 127.81, 127.65, 127.02, 124.38, 119.79, 116.32, 113.87, 55.32. GC-MS (EI, 70 ev): *m*/*z* (%) = 252 (M^+^, 100), 224 (10), 210 (10), 209 (65), 181 (41), 152 (35).

*3-(3-Methoxyphenyl)-2H-chromen-2-one*: ^7t1^H-NMR (300 MHz, Chloroform-*d*) δ 7.81 (s, 1H), 7.53 (td, *J* = 7.4, 1.6 Hz, 2H), 7.40–7.32 (m, 2H), 7.32–7.25 (m, 3H), 6.95 (ddd, *J* = 8.1, 2.6, 1.2 Hz, 1H), 3.85 (s, 3H). ^13^C-NMR (75 MHz, Chloroform-*d*) δ 160.41, 159.48, 153.45, 139.94, 135.96, 131.40, 129.43, 128.09, 127.89, 124.44, 120.86, 119.55, 116.38, 114.47, 114.16, 55.32. GC-MS (EI, 70 ev): *m*/*z* (%) = 252 (M^+^, 100), 224 (46), 194 (10), 182 (10), 181 (68), 167 (10), 165 (21), 153 (13), 152 (62), 151 (16), 127 (10), 126 (14), 63 (16), 39 (10).

*3-(4-Fluorophenyl)-2H-chromen-2-one*: ^7u1^H-NMR (300 MHz, Chloroform-*d*) δ 7.79 (s, 1H), 7.75–7.64 (m, 2H), 7.54 (ddt, *J* = 7.6, 6.0, 1.8 Hz, 2H), 7.37 (dt, *J* = 8.8, 0.8 Hz, 1H), 7.34–7.27 (m, 1H), 7.19–7.06 (m, 2H). ^13^C-NMR (75 MHz, Chloroform-*d*) δ 164.70, 160.51, 153.47, 139.65, 131.48, 130.70, 130.39 (d, *J* = 8.3 Hz), 127.87, 127.30, 124.56, 119.54, 116.47, 115.46 (d, *J* = 21.6 Hz). GC-MS (EI, 70 ev): *m*/*z* (%) = 240 (M^+^, 93), 212 (100), 184 (14), 183 (81), 181 (14), 157 (19), 107 (12), 106 (21), 92 (12), 91 (13).

*3-(3-Fluorophenyl)-2H-chromen-2-one*: ^7y1^H-NMR (300 MHz, Chloroform-*d*) δ 7.79 (s, 1H), 7.56–7.47 (m, 2H), 7.47–7.38 (m, 2H), 7.38–7.28 (m, 2H), 7.27–7.19 (m, 1H), 7.05 (tdd, *J* = 8.3, 2.6, 1.1 Hz, 1H). ^13^C-NMR (75 MHz, Chloroform-*d*) δ 164.24, 160.57 (d, *J* = 61.2 Hz), 153.54, 140.38, 136.63 (d, *J* = 8.1 Hz), 131.78, 129.95 (d, *J* = 8.4 Hz), 128.04, 126.99 (d, *J* = 2.4 Hz), 124.61, 124.13 (d, *J* = 3.1 Hz), 119.36, 116.49, 115.84 (d, *J* = 7.0 Hz), 115.55 (d, *J* = 8.9 Hz). GC-MS (EI, 70 ev): *m*/*z* (%) = 240 (M^+^, 80), 212 (90), 183 (100), 157 (10), 63 (10).

*Dibenzo[b,f]oxepine-10-carbonitrile*: ^91^H-NMR (300 MHz, Chloroform-*d*) δ 7.62 (dd, *J* = 8.1, 1.7 Hz, 1H), 7.54–7.41 (m, 3H), 7.35–7.18 (m, 5H). ^13^C-NMR (75 MHz, Chloroform-*d*) δ 158.30, 157.43, 142.37, 132.86, 131.91, 130.42, 128.29, 128.03, 126.17, 125.60, 125.41, 121.91, 121.67, 118.46, 113.99.GC-MS (EI, 70 ev): *m*/*z* (%) = 219 (M^+^, 100), 191 (25), 190 (93), 165 (12), 164 (30), 163 (25), 82 (10), 63 (12).

*3-(3-Chlorophenyl)-2H-chromen-2-one*: ^1^H-NMR (300 MHz, Chloroform-*d*) δ 7.83 (s, 1H), 7.70 (td, *J* = 1.7, 1.0 Hz, 1H), 7.64–7.59 (m, 1H), 7.59–7.51 (m, 2H), 7.42–7.37 (m, 2H), 7.37–7.28 (m, 2H). ^13^C-NMR (75 MHz, Chloroform-*d*) δ 160.15, 153.59, 140.43, 136.35, 134.38, 131.82, 129.68, 128.89, 128.54, 128.05, 126.96, 126.74, 124.63, 119.37, 116.53. GC-MS (EI, 70 ev): *m*/*z* (%) = 256 (M^+^, 100), 230 (27), 229 (17), 228 (95), 166 (10), 165 (80), 164 (22), 163 (27), 139 (12), 110 (10), 82 (13), 75 (12), 63 (12). HRMS (EI): Calcd. for [[M + H]^+^: C_15_H_9_ClO_2_]^+^: 257.03638, found: 257.03614.

*3-(4-Chlorophenyl)-2H-chromen-2-one*: ^7t1^H-NMR (300 MHz, Chloroform-*d*) δ 7.82 (d, *J* = 0.6 Hz, 1H), 7.71–7.62 (m, 2H), 7.59–7.50 (m, 2H), 7.46–7.40 (m, 2H), 7.37 (dt, *J* = 8.9, 0.8 Hz, 1H), 7.34–7.28 (m, 1H). ^13^C-NMR (75 MHz, Chloroform-*d*) δ 160.32, 153.52, 139.91, 134.92, 133.05, 131.66, 129.82, 128.67, 127.95, 127.15, 124.60, 119.46, 116.50. GC-MS (EI, 70 ev): *m*/*z* (%) = 256 (M^+^, 100), 230 (24), 229 (10), 228 (73), 165 (60), 164 (18), 163 (20).

*3-(Pyridin-3-yl)-2H-chromen-2-one*: ^7z1^H-NMR (300 MHz, Chloroform-*d*) δ 8.80 (d, *J* = 2.4 Hz, 1H), 8.61 (dd, *J* = 4.9, 1.7 Hz, 1H), 8.05 (d, *J* = 8.5 Hz, 1H), 7.28–7.22 (m, 1H), 7.44–7.31 (m, 3H), 7.25 (s, 1H), 7.14 (td, *J* = 7.6, 1.0 Hz, 2H). ^13^C-NMR (75 MHz, Chloroform-*d*) δ 153.33, 149.55, 149.14, 136.43, 134.27, 132.25, 130.90, 127.68, 123.61, 122.92, 119.62, 115.38. GC-MS (EI, 70 ev): *m*/*z* (%) = 221 (M+, 100), 222 (26), 139 (12).

*4-Methyl-3-phenyl-2H-chromen-2-one*: ^7t1^H-NMR (300 MHz, Chloroform-*d*) δ 7.69 (dd, *J* = 8.0, 1.5 Hz, 1H), 7.55 (ddd, *J* = 8.6, 7.2, 1.5 Hz, 1H), 7.50–7.36 (m, 4H), 7.36–7.28 (m, 3H), 2.32 (s, 3H). ^13^C-NMR (75 MHz, Chloroform-*d*) δ 160.93, 152.66, 147.59, 134.42, 131.29, 129.99, 128.40, 128.18, 127.33, 125.08, 124.22, 120.54, 116.85, 16.56. GC-MS (EI, 70 ev): *m*/*z* (%) = 236 (M^+^, 96), 235 (82), 208 (60), 207 (100), 179 (24), 178 (62), 177 (11), 176 (13), 165 (22), 152 (22), 151 (10), 139 (15), 131 (28), 115 (20), 102 (12), 89 (23), 77 (21), 75 (10), 63 (21), 51 (17), 50 (10), 39 (15).

*3,4-Diphenyl-2H-chromen-2-one*: ^101^H-NMR (300 MHz, Chloroform-*d*) δ 7.54 (ddd, *J* = 8.6, 6.6, 2.2 Hz, 1H), 7.44 (ddd, *J* = 8.3, 1.2, 0.6 Hz, 1H), 7.34–7.28 (m, 3H), 7.23–7.10 (m, 9H). ^13^C-NMR (75 MHz, Chloroform-*d*) δ 161.26, 153.22, 151.57, 134.46, 133.84, 131.43, 130.51, 129.35, 128.33, 128.25, 127.78, 127.73, 127.63, 126.99, 124.11, 120.51, 116.76. GC-MS (EI, 70 ev): *m*/*z* (%) = 298 (M^+^, 100), 297 (90), 281 (11), 270 (28), 269 (28), 268 (16), 255 (13), 253 (17), 252 (11), 241 (32), 240 (10), 239 (47), 165 (12), 119 (19).

*2H-Chromen-2-one*: ^7m1^H-NMR (300 MHz, Chloroform-*d*) δ 7.62 (d, *J* = 9.6 Hz, 1H), 7.50–7.35 (m, 2H), 7.30–7.13 (m, 2H), 6.34 (d, *J* = 9.5 Hz, 1H). ^13^C-NMR (75 MHz, Chloroform-*d*) δ 160.74, 154.03, 143.39, 131.80, 127.83, 124.39, 118.81, 116.88, 116.69. GC-MS (EI, 70 ev): *m*/*z* (%) = 146 (M^+^, 56), 118 (100), 90 (44), 89 (41), 64 (10), 63 (28), 62 (12).

*(Z)-2,3-Diphenylacrylonitrile*: ^111^H-NMR (300 MHz, Chloroform-*d*) δ 7.98–7.85 (m, 2H), 7.73–7.64 (m, 2H), 7.55 (s, 1H), 7.52–7.39 (m, 6H). ^13^C-NMR (75 MHz, Chloroform-*d*) δ 142.20, 134.41, 133.66, 130.49, 129.22, 129.16, 129.02, 128.91, 125.95, 117.95, 111.64. GC-MS (EI, 70 ev): *m*/*z* (%) = 205 (M^+^, 100), 204 (92), 203 (26), 190 (52), 178 (23), 177 (27), 176 (24), 165 (13), 151 (13), 102 (12), 89 (14), 88 (11), 77 (11), 76 (16), 75 (11), 63 (13), 51 (22), 50 (14), 39 (11).

*(Z)-3-(4-Hydroxyphenyl)-2-phenylacrylonitrile*: ^111^H-NMR (300 MHz, DMSO-*d*_6_) δ 10.29 (s, 1H), 7.93–7.76 (m, 3H), 7.75–7.64 (m, 2H), 7.54–7.43 (m, 2H), 7.42–7.31 (m, 1H), 6.92 (d, *J* = 8.7 Hz, 2H). ^13^C-NMR (75 MHz, DMSO-*d*_6_) δ 160.02, 142.91, 134.37, 131.44, 129.14, 128.61, 125.41, 124.79, 118.64, 115.89, 105.85. GC-MS (EI, 70 ev): *m*/*z* (%) = 221 (M^+^, 100), 206 (18), 204 (10), 203 (11), 202 (24), 192 (11), 191 (14) 190 (19), 177 (11), 165 (40), 164 (13), 63 (12), 51 (16), 39 (10).

## 4. Conclusions

In summary, a practical procedure for the synthesis of 3-aryl-2*H*-chromen-2-ones from salicylaldehydes and arylacetonitriles has been established. With *^t^*BuOK as the promotor and DMF as the solvent, good to excellent yields of chromenones were obtained. Additionally, no protection of inert gas atmosphere is required here.

## Figures and Tables

**Table 1:**
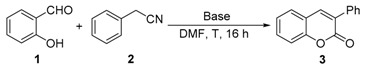
Optimization of the reaction conditions ^[a]^.

Entry	Base	Solvent	T (°C)	Yield (%) ^[b]^
1	*^t^*BuOK	DMF ^[c]^	110	77
2	*^t^*BuOK ^[d]^	DMF ^[c]^	110	75
3	*^t^*BuOK	DMF	110	81
4	*^t^*BuOK	DMF	110	48
				80
5	K_2_CO_3_	DMF	110	34
6	K_3_PO_4_	DMF	110	55
7	KOH	DMF	110	27
8	*^t^*BuOLi	DMF	110	75
9	NaOMe	DMF	110	55
10	*^t^*BuONa	DMF	110	74
11	*^t^*BuOK	DMF	90	73
12	*^t^*BuOK	DMF	130	41
13	*^t^*BuOK	DMAc	110	54
14	*^t^*BuOK	DMSO	110	30
15	*^t^*BuOK	Toluene	110	9
16	*^t^*BuOK	*o*-xylene	110	12
17	*^t^*BuOK	1,4-dioxane	110	15

^[a]^ Reaction conditions: **1** (1 mmol), **2** (1.5 mmol), base (2.0 equiv.), solvent (1 mL), 110 °C, 16 h. ^[b]^ Isolated yields. ^[c]^ DMF (2 mL). ^[d]^ *^t^*BuOK (3 equiv.).

**Table 2:**
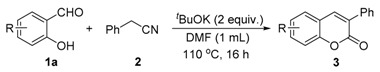
Synthesis of chromenones from salicylaldehydes ^[a]^.

Entry	Substrate	Product	Yield (%) ^[b]^
1	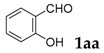	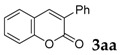	81
2	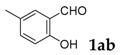	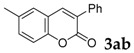	77
3	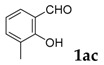	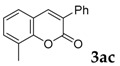	65
4	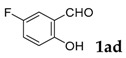	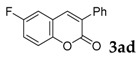	93
5	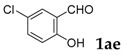	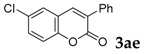	66
6	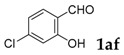	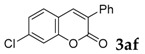	96
7	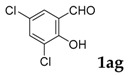	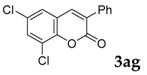	52
8	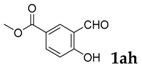	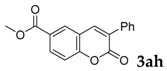	40
9	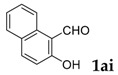	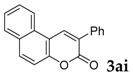	90

^[a]^ Reaction conditions: **1a** (1 mmol), **2** (1.5 mmol), *^t^*BuOK (2.0 equiv.), DMF (1 mL), 110 °C, 16 h. ^[b]^ Isolated yields.

**Table 3:**
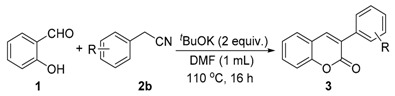
Synthesis of chromenones from 2-arylacetonitriles ^[a]^.

Entry	Substrate	Product	Yield (%) ^[b]^
1	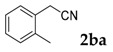	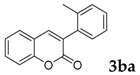	81
2	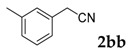	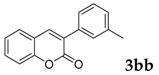	77
3	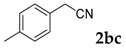	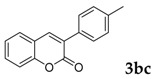	65
4	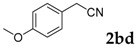	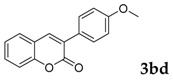	70
5	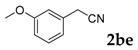	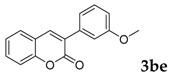	70
6	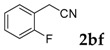	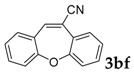	86
7	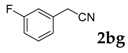	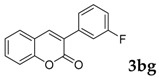	78
8	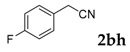	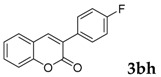	93
9	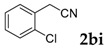	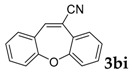	68
10	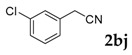	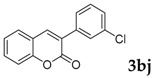	66
11	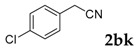	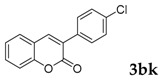	70
12	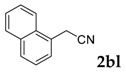	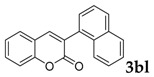	40
13	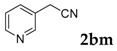	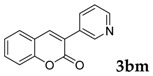	51
14	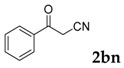	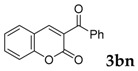	0
15	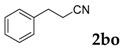	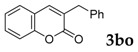	0
16	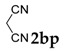	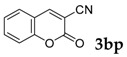	0

^[a]^ Reaction conditions: **1** (1 mmol), **2b** (1.5 mmol), *^t^*BuOK (2.0 equiv.), DMF (1 mL), 110 °C, 16 h. ^[b]^ Isolated yields.

## Supplementary Materials

Supplementary materials are available online.
