# Supplementary material for: Synthesis of 2H-Chromenones from Salicylaldehydes and Arylacetonitriles
Source: Molecules. 2017 Jul 18;22(7):1197. doi: 10.3390/molecules22071197 (PMC6152355; doi:10.3390/molecules22071197)
Supplement: Supplementary file 1 [file molecules-22-01197-s001.pdf]

# Synthesis of 2*H*-Chromenones from Salicylaldehydes and Arylacetonitriles

**Chengcai Li [1], Hailin Zhu [1,2,*], Hang Zhang [1], Yongfeng Yang [1] and Feng Wang [1,2]**

## 3-Phenyl-2*H*-chromen-2-one

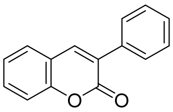


**$^{1}$H NMR (300 MHz, Chloroform-*d*)** δ 7.74 (d, *J* = 0.6 Hz, 1H), 7.66 – 7.60 (m, 2H), 7.50 – 7.41 (m, 2H), 7.41 – 7.32 (m, 3H), 7.29 (dq, *J* = 7.7, 0.9 Hz, 1H), 7.25 – 7.19 (m, 1H).

**$^{13}$C NMR (75 MHz, Chloroform-*d*)** δ 160.55, 153.48, 139.83, 134.67, 131.36, 128.83, 128.49, 128.44, 128.33, 127.87, 124.46, 119.64, 116.42.

**GC-MS (EI, 70ev):** m/z(%) = 222 (M+, 100), 195 (14), 194 (93), 166 (12), 165 (89), 164 (16), 163 (10), 82 (11).

## 6-Methyl-3-phenyl-2*H*-chromen-2-one

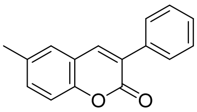


**$^{1}$H NMR (300 MHz, Chloroform-*d*)**δ 7.70 (s, 1H), 7.67 – 7.58 (m, 2H), 7.48 – 7.33 (m, 3H), 7.31 – 7.24 (m, 2H), 7.23 – 7.14 (m, 1H), 2.36 (s, 3H).

**$^{13}$C NMR (75 MHz, Chloroform-*d*)** δ 160.74, 151.61, 139.84, 134.81, 134.11, 132.40, 128.71, 128.48, 128.40, 128.14, 127.65, 119.36, 116.11, 20.76.

**GC-MS (EI, 70ev):** m/z(%) = 236 (M+, 100), 209 (10), 208 (67), 207 (62), 179 (24), 178 (40), 152 (16), 139 (10), 89 (12), 77 (13), 76 (12), 51 (11).

## 6-Fluoro-3-phenyl-2*H*-chromen-2-one

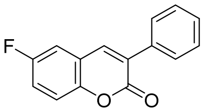


**$^{1}$H NMR (300 MHz, Chloroform-*d*)** δ 7.75 (s, 1H), 7.73 – 7.67 (m, 2H), 7.50 – 7.41 (m, 3H), 7.35 (dddd, *J* = 8.8, 4.5, 1.8, 1.1 Hz, 1H), 7.29 – 7.19 (m, 2H).

**$^{13}$C NMR (75 MHz, Chloroform-*d*)** δ 160.17, 149.63, 138.73, 134.28, 129.52, 129.16, 128.54, 128.53, 120.28, 118.76 (d, *J* = 24.6 Hz), 118.05, 117.94, 113.05 (d, *J* = 23.9 Hz).

**GC-MS (EI, 70ev):** m/z(%) = 240 (M+, 94), 213 (15), 212 (96), 184 (15),183 (100), 182 (12), 181 (10), 163 (11), 157 (13), 91 (10).

HRMS(EI): Calcd. for $[(M+H)^{+}$: $C_{15}H_{9}FO_{2}]^{+}$:241.06593, found: 241.06566.

## 6-Chloro-3-phenyl-2*H*-chromen-2-one

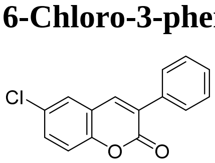

**$^{1}$H NMR (300 MHz, Chloroform-*d*)** δ 7.73 (t, *J* = 0.5 Hz, 1H), 7.72 – 7.66 (m, 2H), 7.53 (d, *J* = 2.4 Hz, 1H), 7.50 – 7.41 (m, 4H), 7.31 (dt, *J* = 8.8, 0.6 Hz, 1H).

**$^{13}$C NMR (75 MHz, Chloroform-*d*)** δ 160.02, 151.88, 138.45, 134.25, 131.31, 129.75, 128.69 – 128.43 (m), 129.56, 129.25, 128.58, 127.10, 120.73, 117.93.

**GC-MS (EI, 70ev):** m/z(%) = 256 (M+, 100), 230 (30), 229 (15), 166 (10), 165 (77), 164 (20), 163 (28), 139 (18), 82 (18), 63 (15).

## Methyl 2-oxo-3-phenyl-2*H*-chromene-6-carboxylate

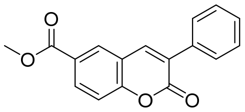

**$^{1}$H NMR (300 MHz, Chloroform-*d*)** δ 8.28 (d, *J* = 2.0 Hz, 1H), 8.19 (dd, *J* = 8.7, 2.0 Hz, 1H), 7.89 – 7.84 (m, 1H), 7.76 – 7.65 (m, 2H), 7.55 – 7.34 (m, 5H), 3.96 (s, 3H).

**$^{13}$C NMR (75 MHz, Chloroform-*d*)** δ 165.70, 159.85, 156.21, 139.23, 134.17, 132.28, 129.94, 129.19, 128.56, 128.50, 128.35, 126.59, 119.39, 116.65, 52.47.

**GC-MS (EI, 70ev):** m/z(%) = 280 (M+, 100), 252 (11), 249 (30), 221 (45), 193 (29), 165 (27), 164 (12), 163 (14), 139 (22), 83 (15).

## 8-Methyl-3-phenyl-2*H*-chromen-2-one

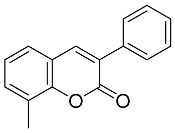

**$^{1}$H NMR (300 MHz, Chloroform-*d*)**δ 7.79 (s, 1H), 7.75 – 7.64 (m, 2H), 7.51 – 7.27 (m, 5H), 7.19 (dd, *J* = 8.1, 7.0 Hz, 1H), 2.49 (s, 3H).

**$^{13}$C NMR (75 MHz, Chloroform-*d*)** δ 160.59, 151.78, 140.19, 134.76, 132.61, 129.02, 128.64, 128.43, 128.35, 127.80, 125.78, 125.56, 123.97, 119.29, 15.38.

**GC-MS (EI, 70ev):** m/z(%) = 236 (M+, 100), 209 (12), 208 (76), 207 (45), 179 (19), 178 (36), 165 (30), 152 (12), 89 (14), 77 (10), 76 (12).

## 6,8-Dichloro-3-phenyl-2*H*-chromen-2-one

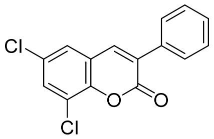

**$^{1}$H NMR (300 MHz, Chloroform-*d*)** δ 7.75 – 7.63 (m, 3H), 7.57 (d, *J* = 2.3 Hz, 1H), 7.48 – 7.39 (m, 4H).

**$^{13}$C NMR (75 MHz, Chloroform-*d*)** δ 158.81, 147.77, 137.89, 133.72, 131.20, 130.23, 129.50, 129.48, 128.60, 128.51, 125.61, 122.27, 121.44.

**GC-MS (EI, 70ev):** m/z(%) = 291 (M+, 63), 290 (94), 266 (11), 265 (10), 264 (65), 263 (16), 262 (100), 201 (20), 200 (10), 199 (62), 164 (28), 163 (60), 162 (10), 139 (10), 99 (16), 87 (11), 81 (19), 63 (10).

## 7-Chloro-3-phenyl-2*H*-chromen-2-one

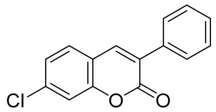

**$^1$H NMR (300 MHz, Chloroform-*d*)** δ 7.66 (s, 1H), 7.61 – 7.52 (m, 2H), 7.44 – 7.30 (m, 4H), 7.28 – 7.24 (m, 1H), 7.21 – 7.09 (m, 1H).

**$^{13}$C NMR (75 MHz, Chloroform-*d*)** δ 159.84, 153.66, 138.90, 137.23, 134.30, 129.03, 128.64, 128.49, 128.44, 128.26, 125.08, 124.91, 118.21, 116.73.

**GC-MS (EI, 70ev):** m/z(%) = 256 (M+, 100), 230 (16), 228 (100), 166 (12), 165 (85), 164 (27), 163 (28), 139 (16), 115 (14), 114 (12), 82 (11), 63 (15).

HRMS(EI): Calcd. for $[(M+H)^+$: $C_{15}H_9ClO_2]^+$:257.03638, found: 257.03614.

## 2-Phenyl-3*H*-benzo[*f*]chromen-3-one

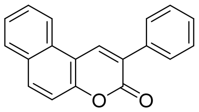

**$^1$H NMR (300 MHz, Chloroform-*d*)**δ 8.36 (d, *J* = 1.7 Hz, 1H), 8.09 (d, *J* = 8.4 Hz, 1H), 7.83 – 7.68 (m, 2H), 7.67 – 7.59 (m, 2H), 7.50 (ddd, *J* = 8.4, 7.0, 1.4 Hz, 1H), 7.42 – 7.26 (m, 5H).

**$^{13}$C NMR (75 MHz, Chloroform-*d*)** δ 160.55, 153.04, 135.60, 135.00, 132.62, 130.23, 129.01, 128.80, 128.50 (d, *J* = 2.1 Hz), 128.12, 127.10, 125.96, 121.34, 116.58, 113.65.

**GC-MS (EI, 70ev):** m/z(%) = 272 (M+, 92), 245 (23), 244 (100), 243 (23), 215 (60), 213 (27), 189 (10), 122 (10), 107 (25), 94 (18).

## 3-(*o*-Tolyl)-2*H*-chromen-2-one

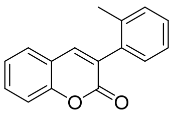

**$^1$H NMR (300 MHz, Chloroform-*d*)** δ 7.65 (s, 1H), 7.60 – 7.46 (m, 2H), 7.39 (ddt, *J* = 8.2, 1.2, 0.6 Hz, 1H), 7.36 – 7.28 (m, 3H), 7.28 – 7.22 (m, 2H).

**$^{13}$C NMR (75 MHz, Chloroform-*d*)** δ 160.21, 153.80, 141.59, 136.82, 134.66, 131.41, 130.30, 129.73, 128.81, 127.81, 125.85, 124.43, 119.28, 116.55, 19.92.

**GC-MS (EI, 70ev):** m/z(%) = 236 (M+, 100), 220 (12), 219 (64), 208 (37), 207 (86), 189 (27), 179 (26), 178 (53), 177 (10), 176 (11), 165 (24), 152 (21), 117 (12), 115 (23), 89 (18), 76 (14), 63 (18), 39 (11).

## 3-(*m*-Tolyl)-2*H*-chromen-2-one

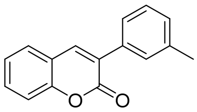

**$^{1}$H NMR (300 MHz, Chloroform-*d*)**δ 7.76 (s, 1H), 7.55 – 7.43 (m, 4H), 7.37 – 7.22 (m, 3H), 7.22 – 7.16 (m, 1H), 2.39 (s, 3H).

**$^{13}$C NMR (75 MHz, Chloroform-*d*)** δ 160.55, 153.42, 139.70, 138.03, 134.59, 131.24, 129.59, 129.09, 128.44, 128.32, 127.81, 125.60, 124.40, 119.65, 116.36, 21.45.

**GC-MS (EI, 70ev):** m/z(%) = 236 (M+, 100), 209 (14), 208 (81), 207 (18), 179 (14), 178 (30), 165 (38), 152 (13), 117 (11), 89 (13), 63 (10).

## 3-(*p*-Tolyl)-2*H*-chromen-2-one

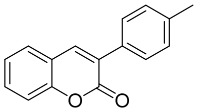

**$^{1}$H NMR (300 MHz, Chloroform-*d*)**δ 7.67 (s, 1H), 7.55 – 7.47 (m, 2H), 7.45 – 7.35 (m, 2H), 7.25 (dt, *J* = 7.8, 0.9 Hz, 1H), 7.21 – 7.09 (m, 3H), 2.29 (s, 3H).

**$^{13}$C NMR (75 MHz, Chloroform-*d*)** δ 160.60, 153.32, 139.12, 138.82, 131.71, 131.10, 129.09, 128.31, 128.17, 127.74, 124.36, 119.68, 116.30, 21.23.

**GC-MS (EI, 70ev):** m/z(%) = 236 (M+, 100), 209 (10), 208 (62), 207 (37), 179 (13), 178 (28), 165 (26), 152 (12), 89 (11), 63 (10), 114 (12), 82 (11), 63 (15).

## 3-(Naphthalen-1-yl)-2*H*-chromen-2-one

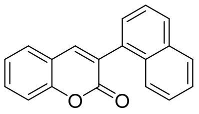

**$^{1}$H NMR (300 MHz, Chloroform-*d*)** δ 7.97 – 7.87 (m, 2H), 7.84 – 7.76 (m, 2H), 7.64 – 7.41 (m, 7H), 7.38 – 7.30 (m, 1H).

**$^{13}$C NMR (75 MHz, Chloroform-*d*)** δ 160.77, 153.97, 142.77, 133.66, 132.64, 131.65, 131.53, 129.36, 128.53, 128.37, 127.93, 127.63, 126.48, 126.07, 125.23, 124.54, 119.32, 116.68.

**GC-MS (EI, 70ev):** m/z(%) = 272 (M+, 100), 273 (19), 271 (79), 255 (11), 244 (24), 243 (50), 216 (11), 215 (58), 214 (10), 213 (28), 189 (17), 107 (18), 95 (17), 63 (11).

## 3-(4-Methoxyphenyl)-2*H*-chromen-2-one

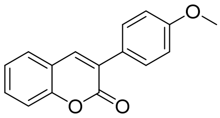

**$^{1}$H NMR (300 MHz, Chloroform-*d*)** δ 7.75 (s, 1H), 7.71 – 7.63 (m, 2H), 7.56 – 7.44 (m, 2H), 7.34 (ddd, *J* = 8.0, 1.3, 0.7 Hz, 1H), 7.31 – 7.23 (m, 1H), 7.02 – 6.90 (m, 2H), 3.85 (s, 3H).

**$^{13}$C NMR (75 MHz, Chloroform-*d*)** δ 160.74, 160.10, 153.24, 138.43, 130.95, 129.78, 127.81, 127.65, 127.02, 124.38, 119.79, 116.32, 113.87, 55.32.

**GC-MS (EI, 70ev):** m/z(%) = 252 (M+, 100), 224 (10), 210 (10), 209 (65), 181 (41), 152 (35).

## 3-(3-Methoxyphenyl)-2*H*-chromen-2-one

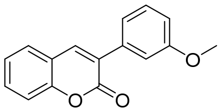

**$^1$H NMR (300 MHz, Chloroform-*d*)**δ 7.81 (s, 1H), 7.53 (td, *J* = 7.4, 1.6 Hz, 2H), 7.40 – 7.32 (m, 2H), 7.32 – 7.25 (m, 3H), 6.95 (ddd, *J* = 8.1, 2.6, 1.2 Hz, 1H), 3.85 (s, 3H).

**$^{13}$C NMR (75 MHz, Chloroform-*d*)** δ 160.41, 159.48, 153.45, 139.94, 135.96, 131.40, 129.43, 128.09, 127.89, 124.44, 120.86, 119.55, 116.38, 114.47, 114.16, 55.32.

**GC-MS (EI, 70ev):** m/z(%) = 252 (M+, 100), 224 (46), 194 (10), 182 (10), 181 (68), 167 (10), 165 (21), 153 (13), 152 (62),151 (16), 127 (10), 126 (14), 63 (16), 39 (10).

## 3-(4-Fluorophenyl)-2*H*-chromen-2-one

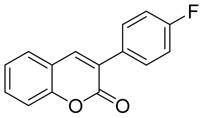

**$^1$H NMR (300 MHz, Chloroform-*d*)** δ 7.79 (s, 1H), 7.75 – 7.64 (m, 2H), 7.54 (ddt, *J* = 7.6, 6.0, 1.8 Hz, 2H), 7.37 (dt, *J* = 8.8, 0.8 Hz, 1H), 7.34 – 7.27 (m, 1H), 7.19 – 7.06 (m, 2H).

**$^{13}$C NMR (75 MHz, Chloroform-*d*)** δ 164.70, 160.51, 153.47, 139.65, 131.48, 130.70, 130.39 (d, *J* = 8.3 Hz), 127.87, 127.30, 124.56, 119.54, 116.47, 115.46 (d, *J* = 21.6 Hz).

**GC-MS (EI, 70ev):** m/z(%) = 240 (M+, 93), 212 (100), 184 (14), 183 (81), 181 (14), 157 (19), 107 (12), 106 (21), 92 (12), 91 (13).

## 3-(3-Fluorophenyl)-2*H*-chromen-2-one

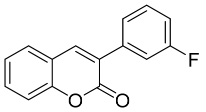

**$^1$H NMR (300 MHz, Chloroform-*d*)** δ 7.79 (s, 1H), 7.56 – 7.47 (m, 2H), 7.47 – 7.38 (m, 2H), 7.38 – 7.28 (m, 2H), 7.27 – 7.19 (m, 1H), 7.05 (tdd, *J* = 8.3, 2.6, 1.1 Hz, 1H).

**$^{13}$C NMR (75 MHz, Chloroform-*d*)** δ 164.24, 160.57 (d, *J* = 61.2 Hz), 153.54, 140.38, 136.63 (d, *J* = 8.1 Hz), 131.78, 129.95 (d, *J* = 8.4 Hz), 128.04, 126.99 (d, *J* = 2.4 Hz), 124.61, 124.13 (d, *J* = 3.1 Hz), 119.36, 116.49, 115.84 (d, *J* = 7.0 Hz), 115.55 (d, *J* = 8.9 Hz).

**GC-MS (EI, 70ev):** m/z(%) = 240 (M+, 80), 212 (90), 183 (100),157 (10), 63 (10).

## Dibenzo[*b,f*]oxepine-10-carbonitrile

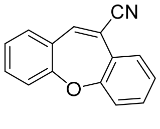

**$^1$H NMR (300 MHz, Chloroform-*d*)** δ 7.62 (dd, *J* = 8.1, 1.7 Hz, 1H), 7.54 – 7.41 (m, 3H), 7.35 – 7.18 (m, 5H).

**$^{13}$C NMR (75 MHz, Chloroform-*d*)** δ 158.30, 157.43, 142.37, 132.86, 131.91, 130.42, 128.29, 128.03, 126.17, 125.60, 125.41, 121.91, 121.67, 118.46, 113.99.

**GC-MS (EI, 70ev):** m/z(%) = 219 (M+, 100), 191 (25), 190 (93), 165 (12), 164 (30), 163 (25), 82 (10), 63 (12).

## 3-(3-Chlorophenyl)-2*H*-chromen-2-one

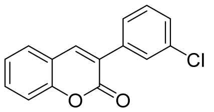


**$^{1}$H NMR (300 MHz, Chloroform-*d*)** δ 7.83 (s, 1H), 7.70 (td, *J* = 1.7, 1.0 Hz, 1H), 7.64 – 7.59 (m, 1H), 7.59 – 7.51 (m, 2H), 7.42 – 7.37 (m, 2H), 7.37 – 7.28 (m, 2H).

**$^{13}$C NMR (75 MHz, Chloroform-*d*)** δ 160.15, 153.59, 140.43, 136.35, 134.38, 131.82, 129.68, 128.89, 128.54, 128.05, 126.96, 126.74, 124.63, 119.37, 116.53.

**GC-MS (EI, 70ev):** m/z(%) = 256 (M+, 100), 230 (27), 229 (17), 228 (95), 166 (10), 165 (80), 164 (22), 163 (27), 139 (12), 110 (10), 82 (13), 75 (12), 63 (12).

HRMS(EI): Calcd. for $[(M+H)^{+}$: $C_{15}H_9ClO_2]^{+}$:257.03638, found: 257.03633.

## 3-(4-Chlorophenyl)-2*H*-chromen-2-one

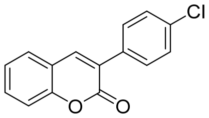


**$^{1}$H NMR (300 MHz, Chloroform-*d*)** δ 7.82 (d, *J* = 0.6 Hz, 1H), 7.71 – 7.62 (m, 2H), 7.59 – 7.50 (m, 2H), 7.46 – 7.40 (m, 2H), 7.37 (dt, *J* = 8.9, 0.8 Hz, 1H), 7.34 – 7.28 (m, 1H).

**$^{13}$C NMR (75 MHz, Chloroform-*d*)** δ 160.32, 153.52, 139.91, 134.92, 133.05, 131.66, 129.82, 128.67, 127.95, 127.15, 124.60, 119.46, 116.50.

**GC-MS (EI, 70ev):** m/z(%) = 256 (M+, 100), 230 (24), 229 (10), 228 (73), 165 (60), 164 (18), 163 (20).

## 3-(Pyridin-3-yl)-2*H*-chromen-2-one

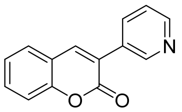


**$^{1}$H NMR (300 MHz, Chloroform-*d*)** δ 8.80 (d, *J* = 2.4 Hz, 1H), 8.61 (dd, *J* = 4.9, 1.7 Hz, 1H), 8.05 (d, *J* = 8.5 Hz, 1H), 7.28 – 7.22 (m, 1H), 7.44 – 7.31 (m, 3H), 7.25 (s, 1H), 7.14 (td, *J* = 7.6, 1.0 Hz, 2H).

**$^{13}$C NMR (75 MHz, Chloroform-*d*)** δ 153.33, 149.55, 149.14, 136.43, 134.27, 132.25, 130.90, 127.68, 123.61, 122.92, 119.62, 115.38.

**GC-MS (EI, 70ev):** m/z(%) = 221 (M+, 100), 222 (26), 139 (12).

## 4-Methyl-3-phenyl-2*H*-chromen-2-one

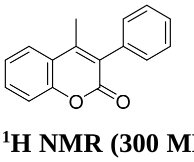


**$^{1}$H NMR (300 MHz, Chloroform-*d*)**δ 7.69 (dd, *J* = 8.0, 1.5 Hz, 1H), 7.55 (ddd, *J* = 8.6, 7.2, 1.5 Hz, 1H), 7.50 – 7.36 (m, 4H), 7.36 – 7.28 (m, 3H), 2.32 (s, 3H).

**$^{13}$C NMR (75 MHz, Chloroform-*d*)** δ 160.93, 152.66, 147.59, 134.42, 131.29, 129.99, 128.40, 128.18, 127.33, 125.08, 124.22, 120.54, 116.85, 16.56.
**GC-MS (EI, 70ev):** m/z(%) = 236 (M+, 96), 235 (82), 208 (60), 207 (100), 179 (24), 178 (62), 177 (11), 176 (13), 165 (22), 152 (22), 151 (10), 139 (15), 131 (28), 115 (20), 102 (12), 89 (23), 77 (21), 75 (10), 63 (21), 51 (17), 50 (10), 39 (15).

## 3,4-Diphenyl-2*H*-chromen-2-one

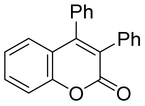


**$^{1}$H NMR (300 MHz, Chloroform-*d*)** δ 7.54 (ddd, *J* = 8.6, 6.6, 2.2 Hz, 1H), 7.44 (ddd, *J* = 8.3, 1.2, 0.6 Hz, 1H), 7.34 – 7.28 (m, 3H), 7.23 – 7.10 (m, 9H).
**$^{13}$C NMR (75 MHz, Chloroform-*d*)** δ 161.26, 153.22, 151.57, 134.46, 133.84, 131.43, 130.51, 129.35, 128.33, 128.25, 127.78, 127.73, 127.63, 126.99, 124.11, 120.51, 116.76.
**GC-MS (EI, 70ev):** m/z(%) = 298 (M+, 100), 297 (90), 281 (11), 270 (28), 269 (28), 268 (16), 255 (13), 253 (17), 252 (11), 241 (32), 240 (10), 239 (47), 165 (12), 119 (19).

## 2*H*-Chromen-2-one

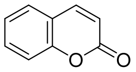


**$^{1}$H NMR (300 MHz, Chloroform-*d*)** δ 7.62 (d, *J* = 9.6 Hz, 1H), 7.50 – 7.35 (m, 2H), 7.30 – 7.13 (m, 2H), 6.34 (d, *J* = 9.5 Hz, 1H).
**$^{13}$C NMR (75 MHz, Chloroform-*d*)** δ 160.74, 154.03, 143.39, 131.80, 127.83, 124.39, 118.81, 116.88, 116.69.
**GC-MS (EI, 70ev):** m/z(%) = 146 (M+, 56), 118 (100), 90 (44), 89 (41), 64 (10), 63 (28), 62 (12).

## (*Z*)-2,3-Diphenylacrylonitrile

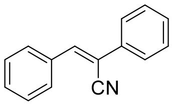


**$^{1}$H NMR (300 MHz, Chloroform-*d*)** δ 7.98 – 7.85 (m, 2H), 7.73 – 7.64 (m, 2H), 7.55 (s, 1H), 7.52 – 7.39 (m, 6H).
**$^{13}$C NMR (75 MHz, Chloroform-*d*)** δ 142.20, 134.41, 133.66, 130.49, 129.22, 129.16, 129.02, 128.91, 125.95, 117.95, 111.64.
**GC-MS (EI, 70ev):** m/z(%) = 205 (M+, 100), 204 (92), 203 (26), 190 (52), 178 (23), 177 (27), 176 (24), 165 (13), 151 (13), 102 (12), 89 (14), 88 (11), 77 (11), 76 (16), 75 (11), 63 (13), 51 (22), 50 (14), 39 (11).

## (*Z*)-3-(4-Hydroxyphenyl)-2-phenylacrylonitrile

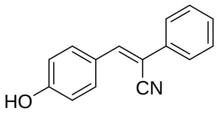

**$^{1}$H NMR (300 MHz, DMSO-*d*$_6$)** δ 10.29 (s, 1H), 7.93 – 7.76 (m, 3H), 7.75 – 7.64 (m, 2H), 7.54 – 7.43 (m, 2H), 7.42 – 7.31 (m, 1H), 6.92 (d, *J* = 8.7 Hz, 2H).

**$^{13}$C NMR (75 MHz, DMSO-*d*$_6$)** δ 160.02, 142.91, 134.37, 131.44, 129.14, 128.61, 125.41, 124.79, 118.64, 115.89, 105.85.

**GC-MS (EI, 70ev):** m/z(%) = 221 (M+, 100), 206 (18), 204 (10), 203 (11), 202 (24), 192 (11), 191 (14) 190 (19), 177 (11), 165 (40), 164 (13), 63 (12), 51 (16), 39 (10).

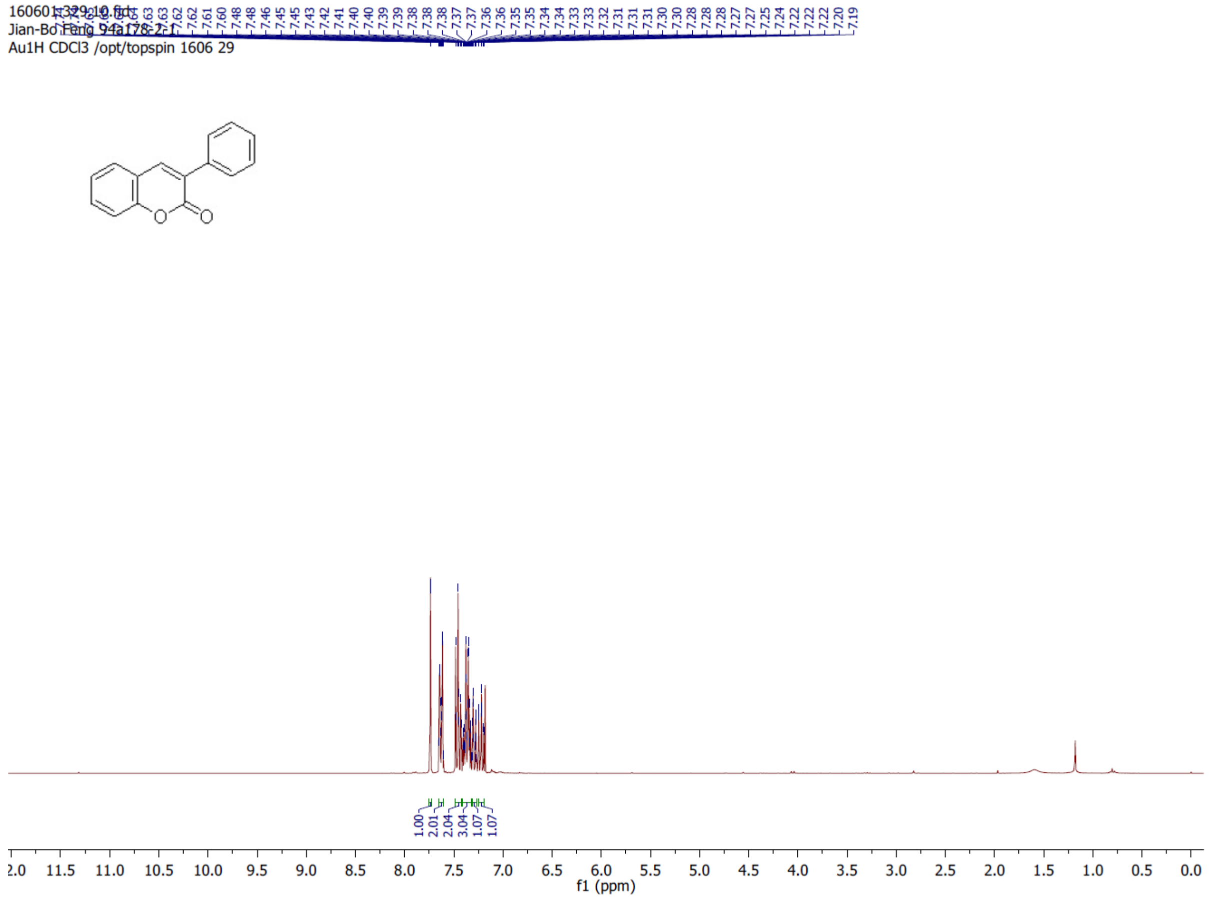

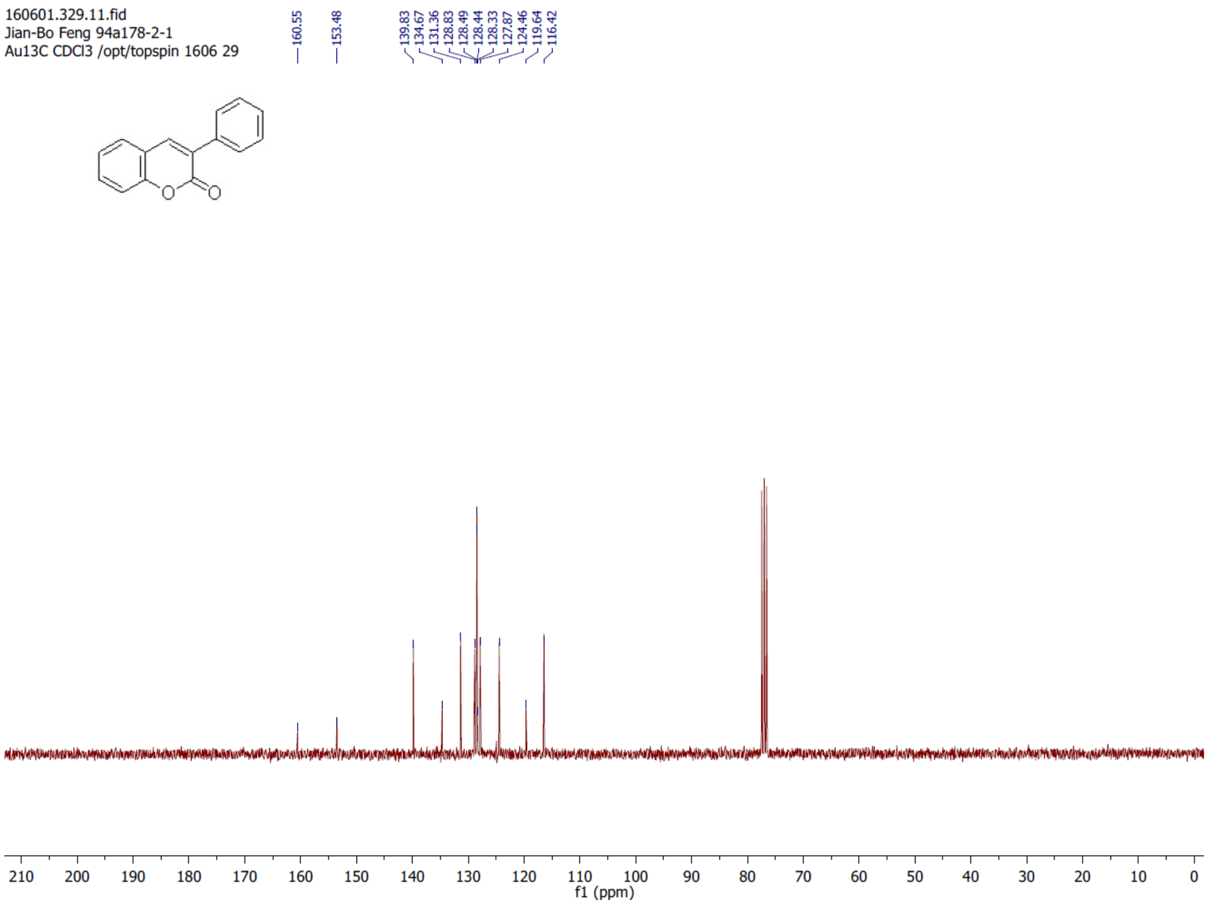

160601.329.11.fid
Jian-Bo Feng 94a178-2-1
Au13C CDCl3 /opt/topspin 1606 29
160.55
153.48
139.83
134.67
131.36
128.83
128.49
128.44
128.33
127.87
124.46
119.64
116.42
210 200 190 180 170 160 150 140 130 120 110 100 90 80 70 60 50 40 30 20 10 0
f1 (ppm)


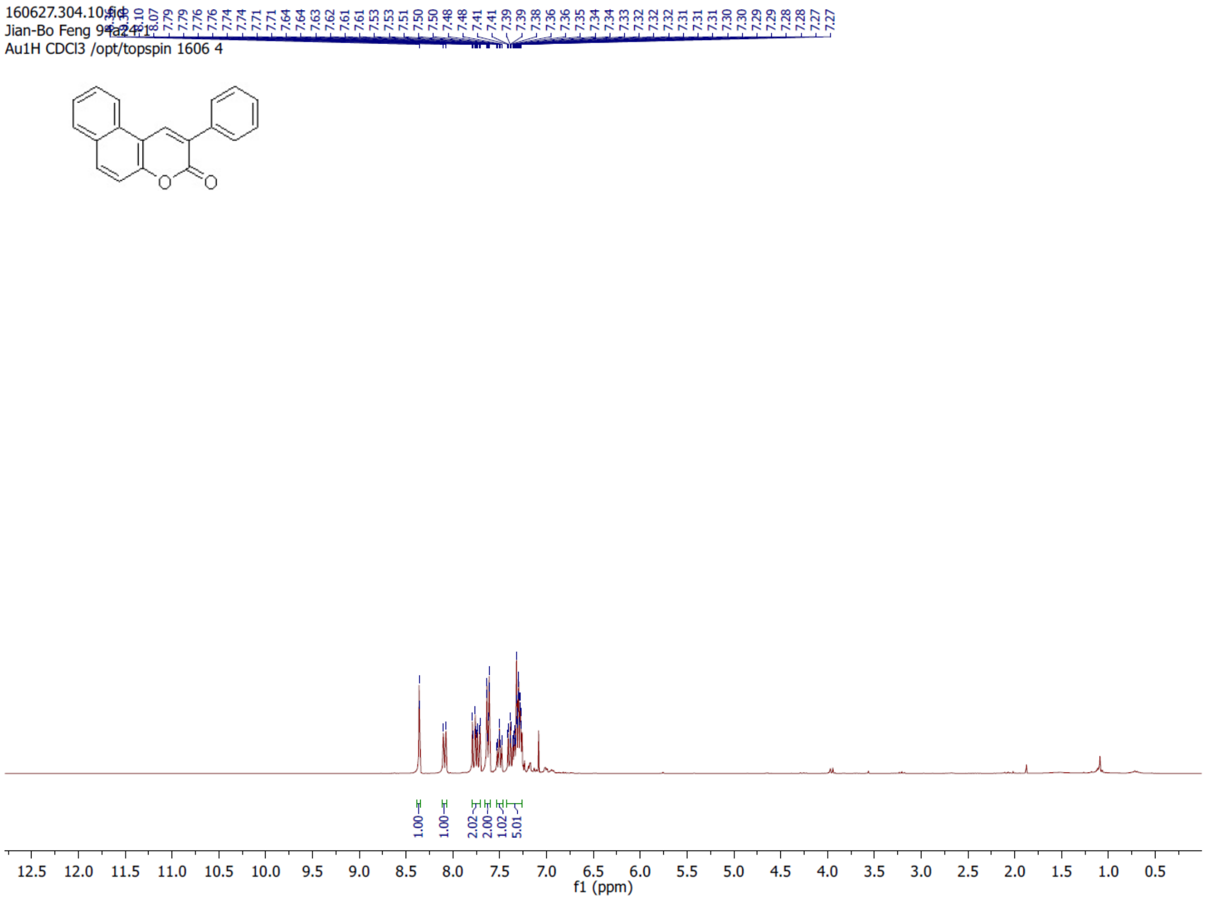

160627.304.10.fid
Jian-Bo Feng 94a24-1
Au1H CDCl3 /opt/topspin 1606 4
8.07
7.79
7.79
7.76
7.76
7.74
7.74
7.71
7.64
7.64
7.63
7.62
7.61
7.61
7.53
7.53
7.51
7.50
7.50
7.48
7.48
7.41
7.41
7.39
7.39
7.38
7.36
7.36
7.35
7.34
7.34
7.33
7.32
7.32
7.32
7.31
7.31
7.30
7.30
7.29
7.29
7.28
7.28
7.27
7.27
1.00
1.00
2.02
2.00
1.02
5.01
12.5 12.0 11.5 11.0 10.5 10.0 9.5 9.0 8.5 8.0 7.5 7.0 6.5 6.0 5.5 5.0 4.5 4.0 3.5 3.0 2.5 2.0 1.5 1.0 0.5
f1 (ppm)

160627.304.11.fid
Jian-Bo Feng 94a24-1
Au13C CDCl3 /opt/topspin 1606 4

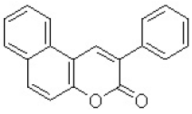

160.55, 153.04, 135.60, 135.00, 132.62, 130.23, 129.01, 128.80, 128.51, 128.49, 128.12, 127.10, 125.96, 121.34, 116.58, 113.65

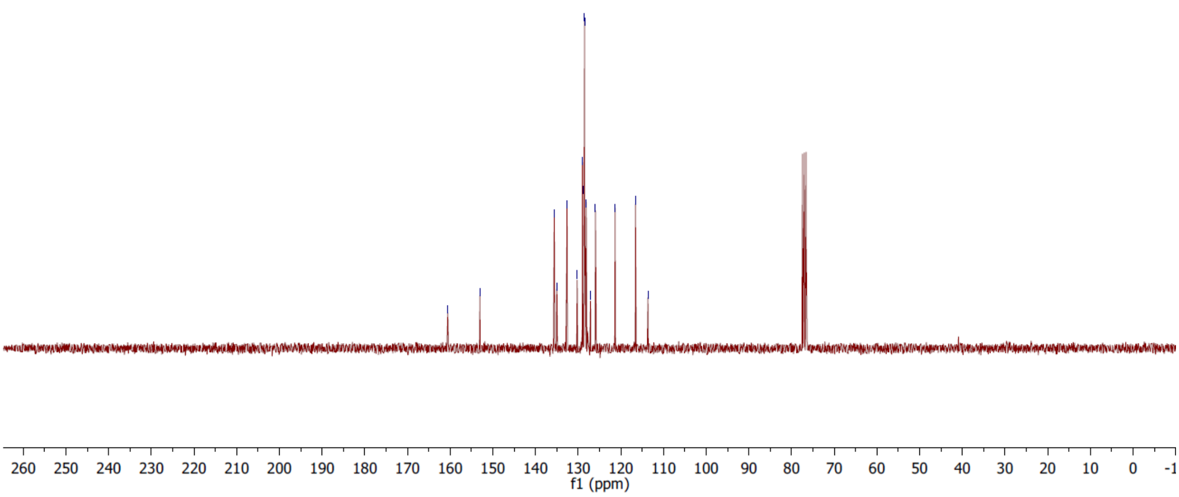


160704.f307.10.fid
Jian-Bo Feng 94a24-2
PROTON CDCl3 {C:\Bruker\TopSpin3.2PL6} 1606 7

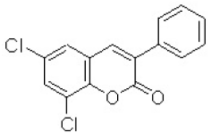


7.71, 7.70, 7.70, 7.69, 7.69, 7.69, 7.68, 7.68, 7.67, 7.57, 7.56, 7.47, 7.47, 7.46, 7.46, 7.45, 7.44, 7.44, 7.43

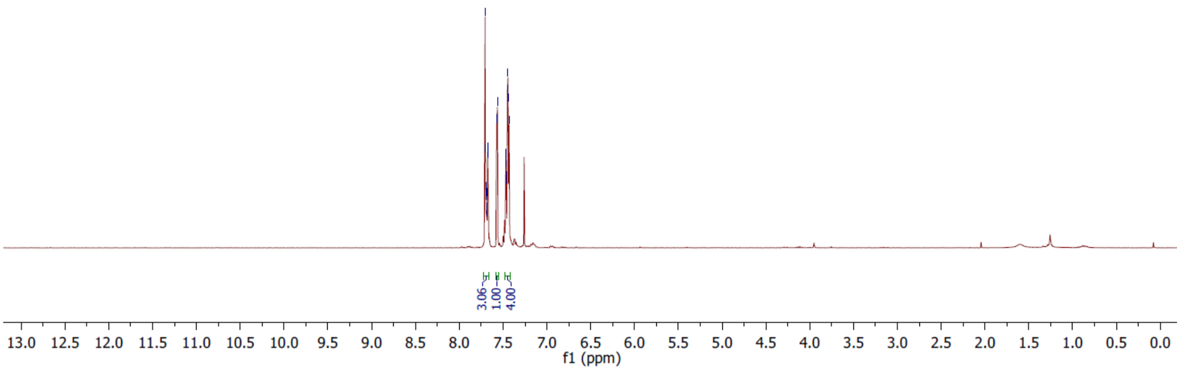

160704.f307.11.fid
Jian-Bo Feng 94a24-2
C13CPD CDCl3 {C:\Bruker\TopSpin3.2PL6} 1606 7

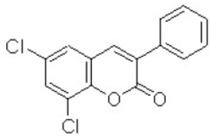


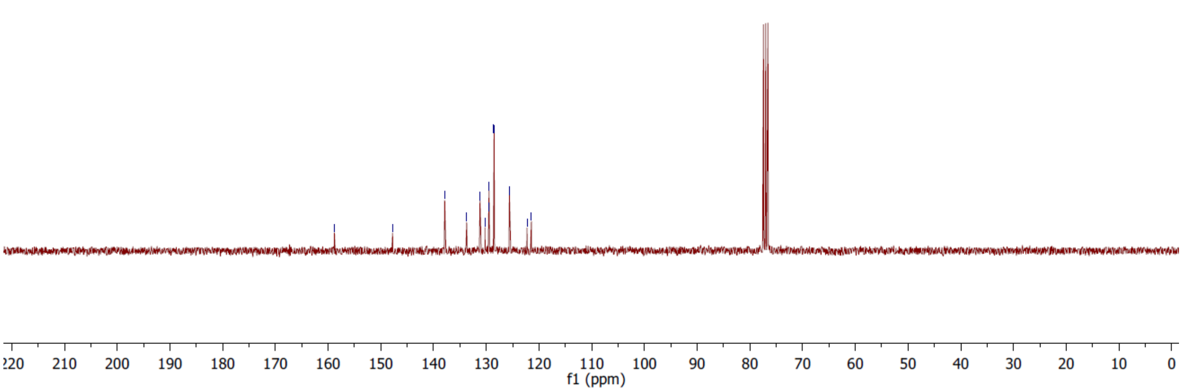


160627.305.10.fid
Jian-Bo Feng 94a24-3
Au1H CDCl3 /opt/topspin 1606 5

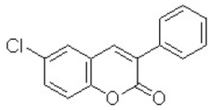


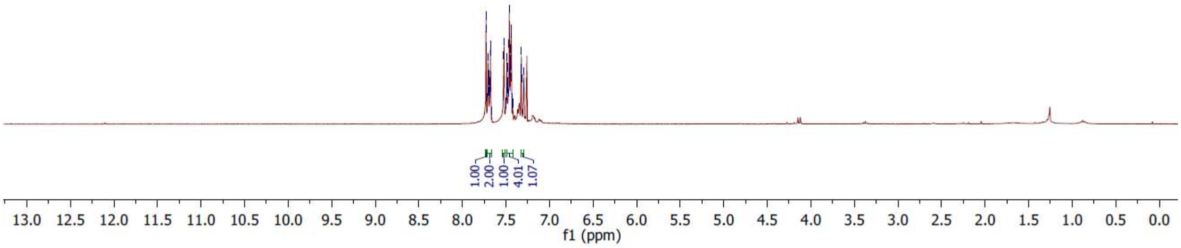

160627.305.11.fid
Jian-Bo Feng 94a24-3
Au13C CDCl3 /opt/topspin 1606 5

160.02, 151.88, 138.45, 134.25, 131.31, 129.75, 129.56, 129.25, 128.58, 127.10, 120.73, 117.93

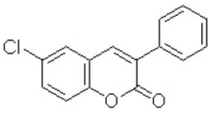

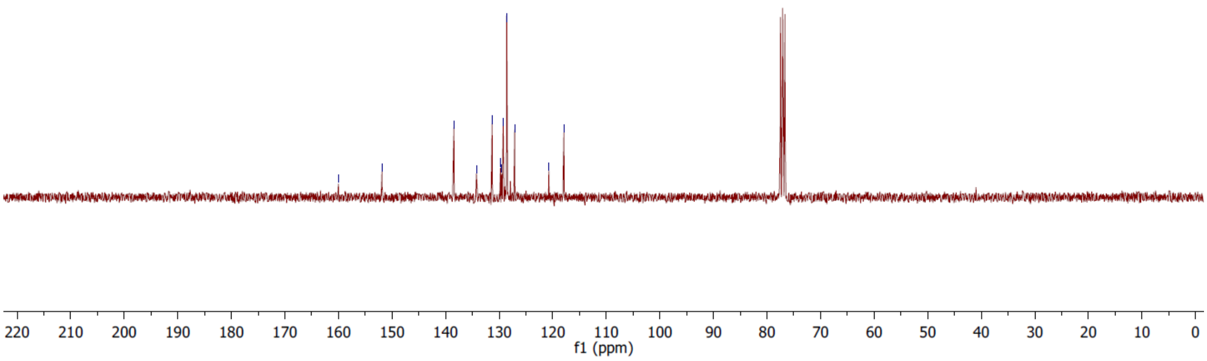

160729.f344.10.fid
Jian-Bo Feng 94a73-2
PROTON CDCl3 {C:\Bruker\TopSpin3.2PL6} 1607 44

7.75, 7.71, 7.71, 7.71, 7.70, 7.70, 7.69, 7.69, 7.68, 7.49, 7.49, 7.48, 7.48, 7.47, 7.47, 7.46, 7.46, 7.45, 7.45, 7.44, 7.44, 7.44, 7.43, 7.43, 7.42, 7.42, 7.42, 7.37, 7.37, 7.37, 7.36, 7.36, 7.36, 7.35, 7.35, 7.35, 7.34, 7.34, 7.34, 7.33, 7.33, 7.33, 7.32, 7.28, 7.27, 7.26, 7.25, 7.25, 7.24, 7.24, 7.22, 7.22, 7.21

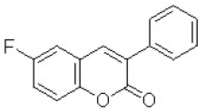

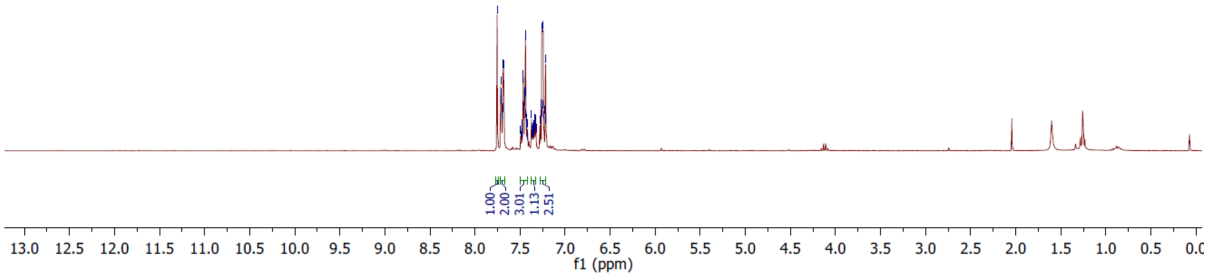

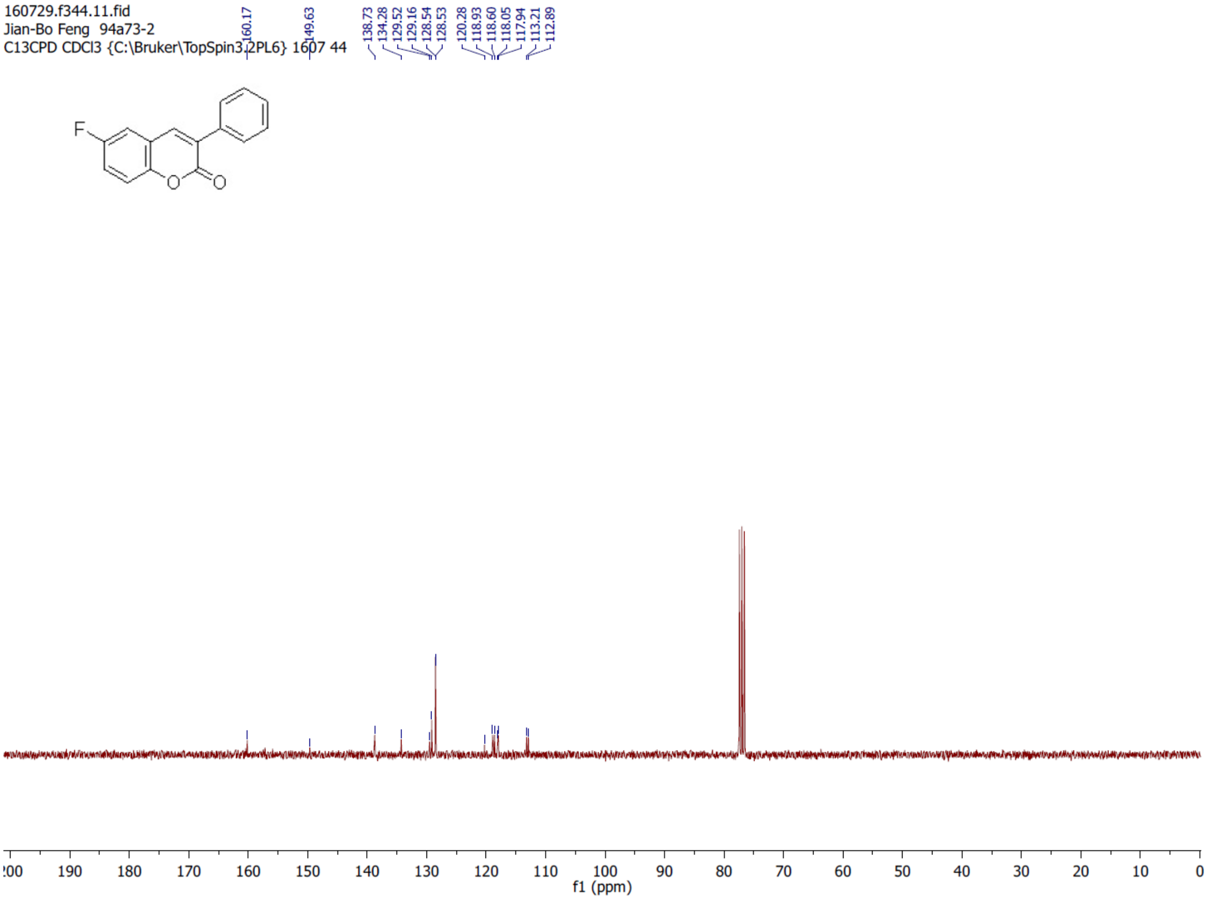
160729.f344.11.fid
Jian-Bo Feng 94a73-2
C13CPD CDCl3 {C:\Bruker\TopSpin3.2PL6} 1607 44
160.17
149.63
138.73
134.28
129.52
129.16
128.54
128.53
120.28
118.93
118.60
118.05
117.94
113.21
112.89
F
O
O
f1 (ppm)

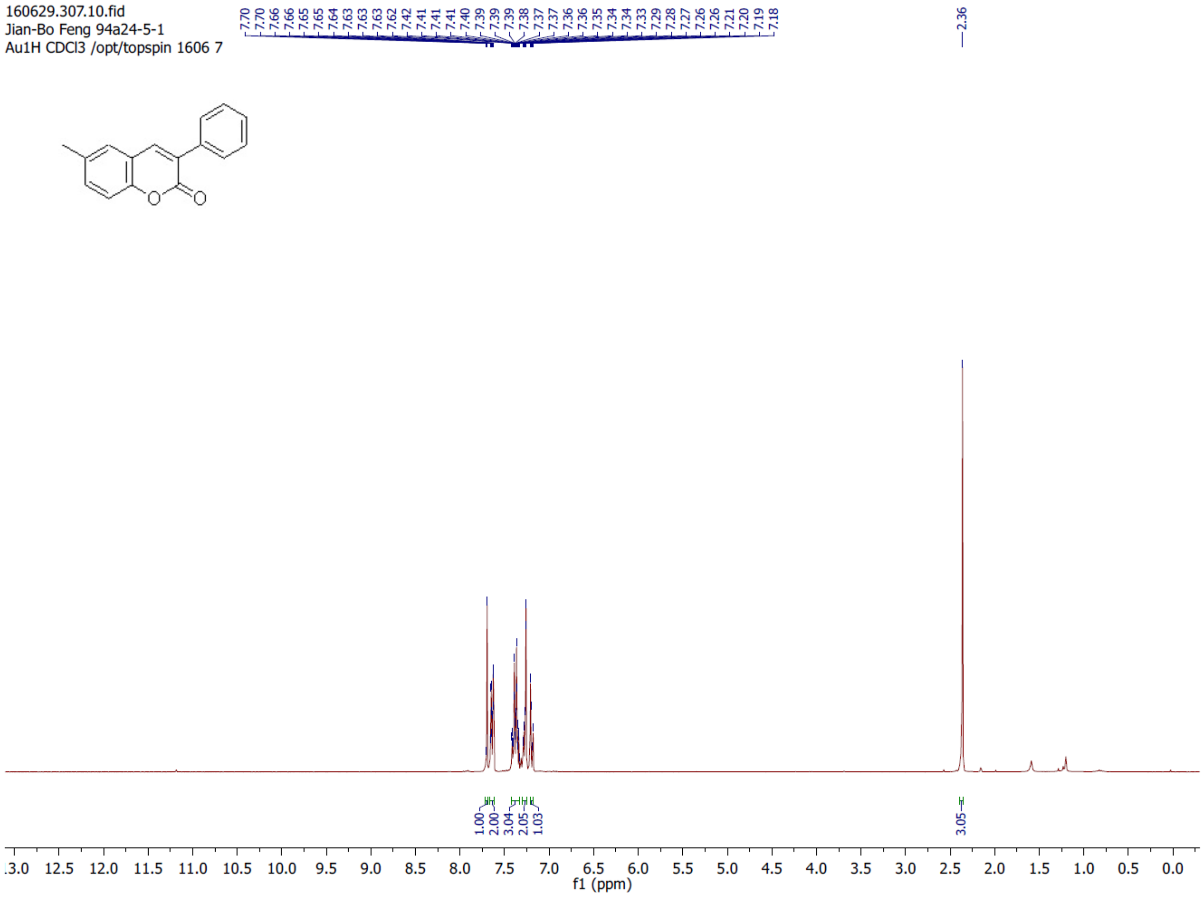
160629.307.10.fid
Jian-Bo Feng 94a24-5-1
Au1H CDCl3 /opt/topspin 1606 7
7.70
7.70
7.66
7.65
7.65
7.64
7.63
7.63
7.62
7.42
7.41
7.41
7.41
7.40
7.39
7.39
7.39
7.38
7.37
7.37
7.36
7.36
7.35
7.34
7.34
7.33
7.29
7.28
7.27
7.26
7.26
7.21
7.20
7.19
7.18
2.36
O
O
1.00
2.00
3.04
2.05
1.03
3.05
f1 (ppm)

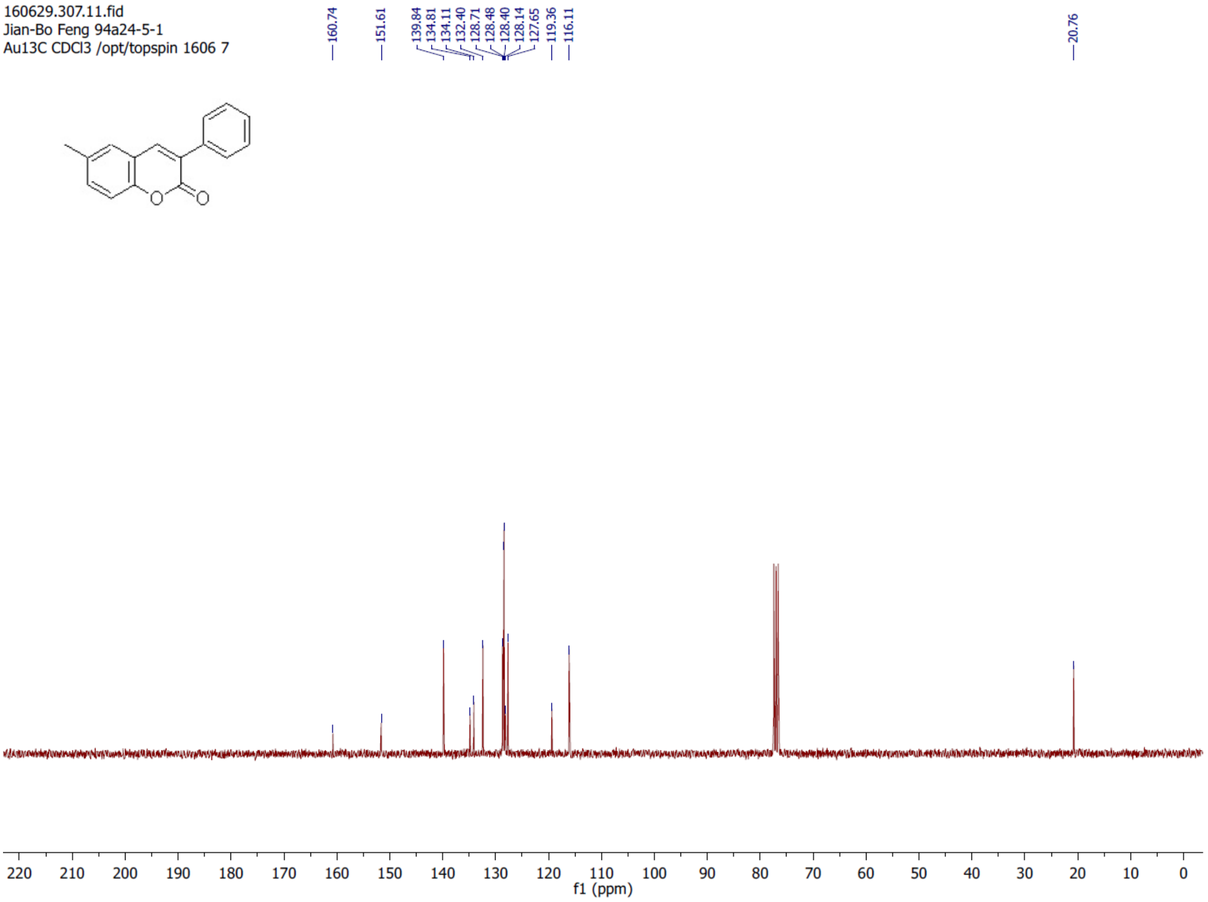

160629.307.11.fid
Jian-Bo Feng 94a24-5-1
Au13C CDCl3 /opt/topspin 1606 7
160.74
151.61
139.84
134.81
134.11
132.40
128.71
128.48
128.40
128.14
127.65
119.36
116.11
20.76
f1 (ppm)


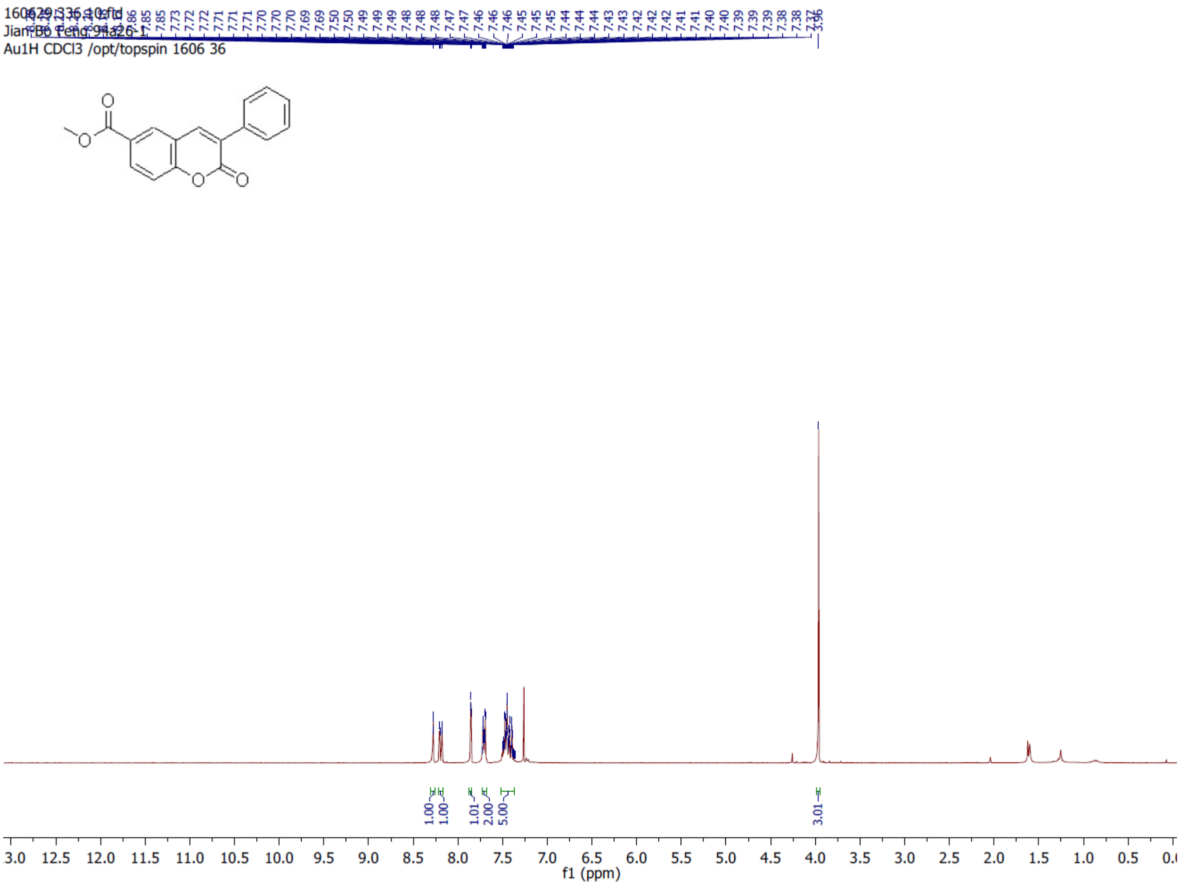

160629.336.10.fid
Jian-Bo Feng 94a26-1
Au1H CDCl3 /opt/topspin 1606 36
3.96
1.00
1.00
1.01
2.00
5.00
3.01
f1 (ppm)

160629.336.11.fid
Jian-Bo Feng 94a26-1
Au13C CDCl3 /opt/topspin 1606 36

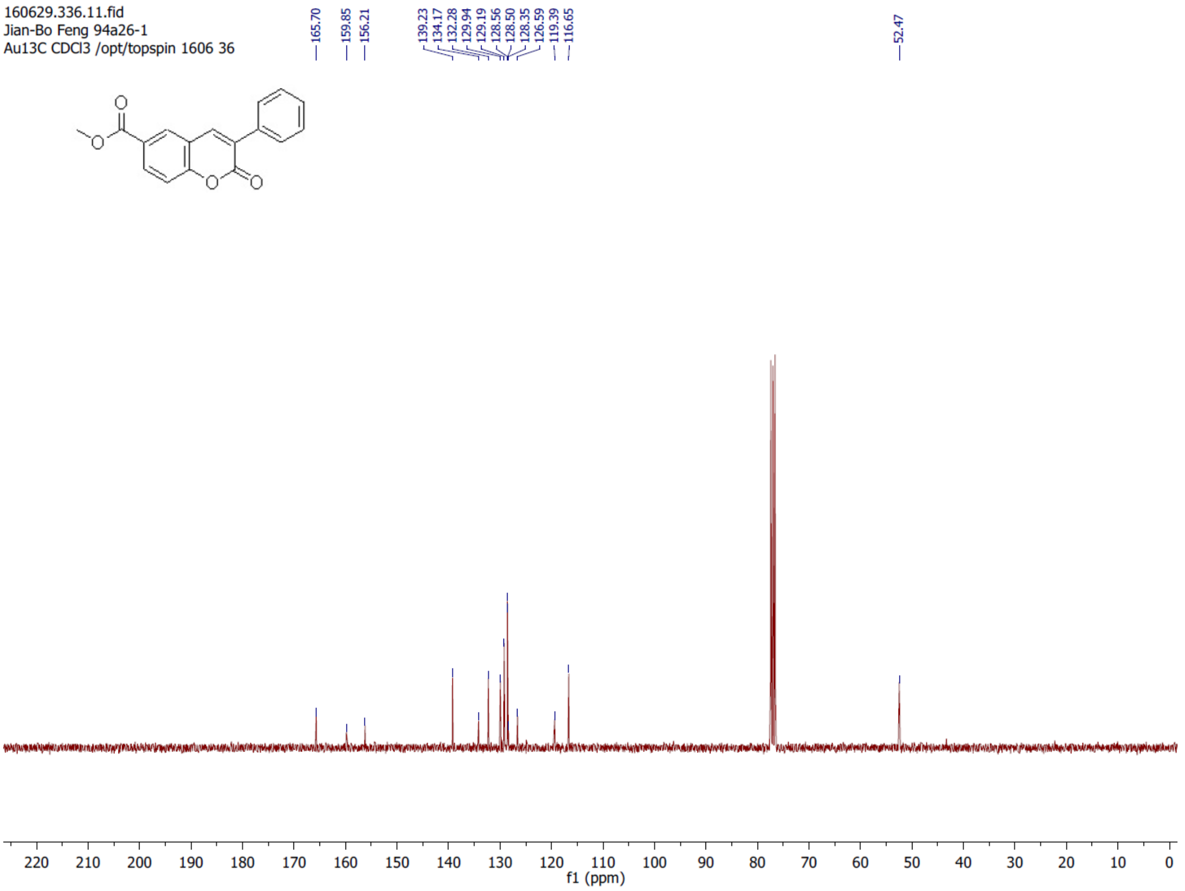


160629.337.10.fid
Jian-Bo Feng 94a26-2
Au1H CDCl3 /opt/topspin 1606 37

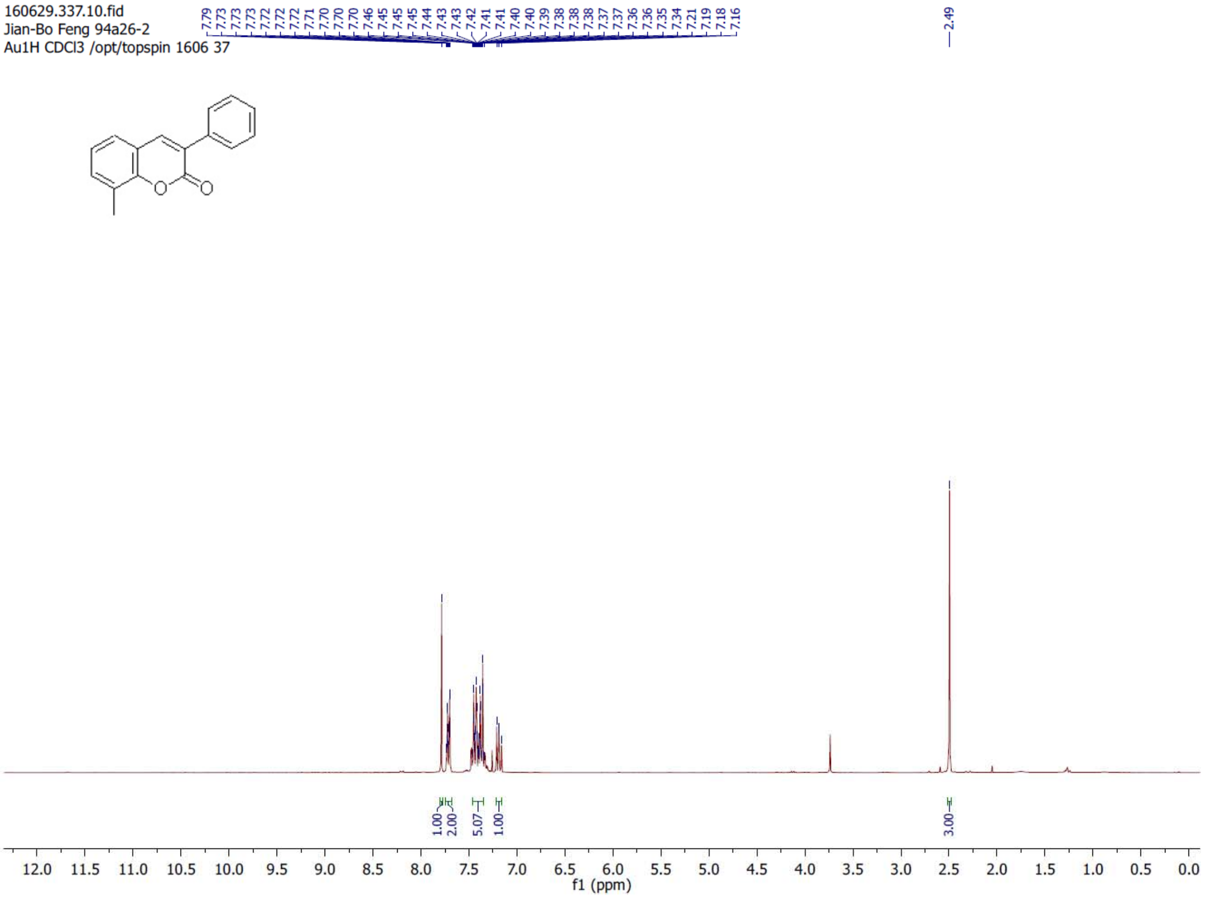

160629.337.11.fid
Jian-Bo Feng 94a26-2
Au13C CDCl3 /opt/topspin 1606 37

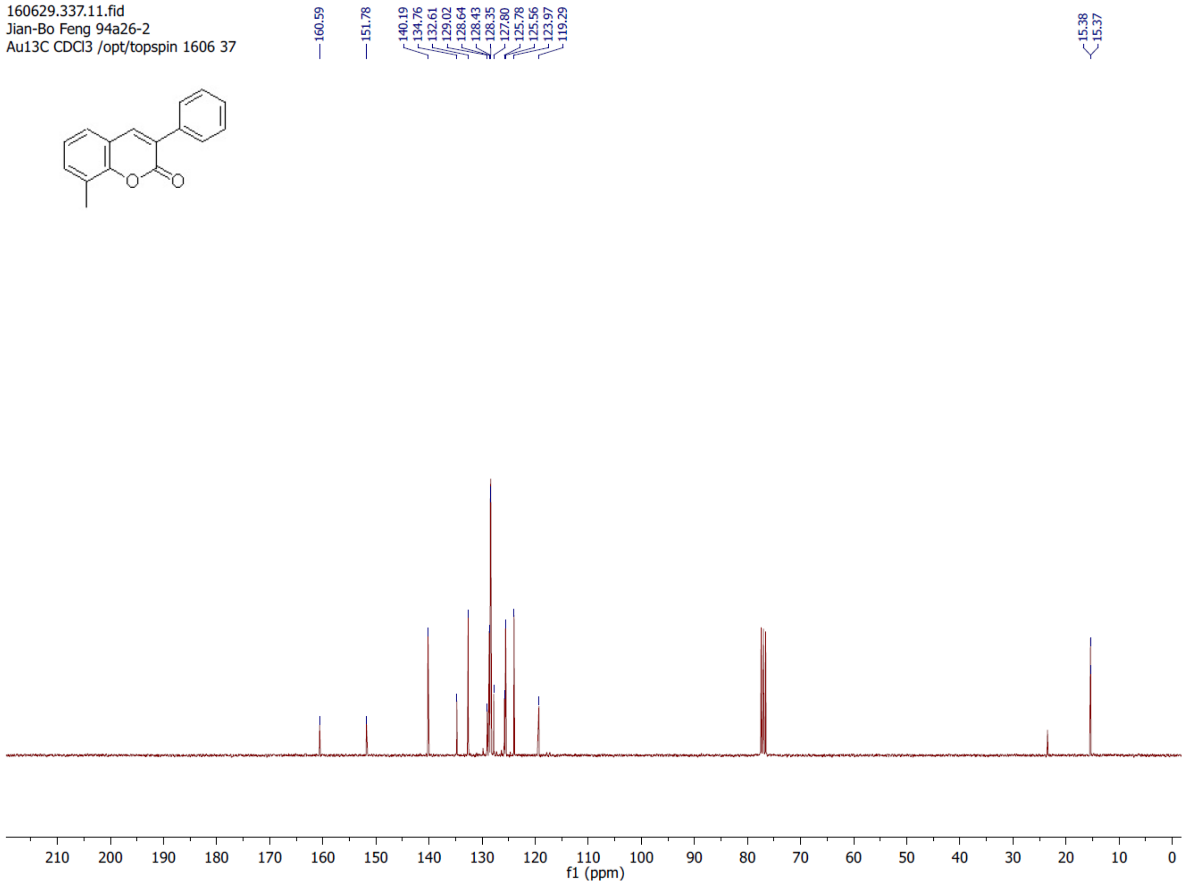


160707.306.10.fid
Jian-Bo Feng 94a42-2
Au1H CDCl3 /opt/topspin 1607 6

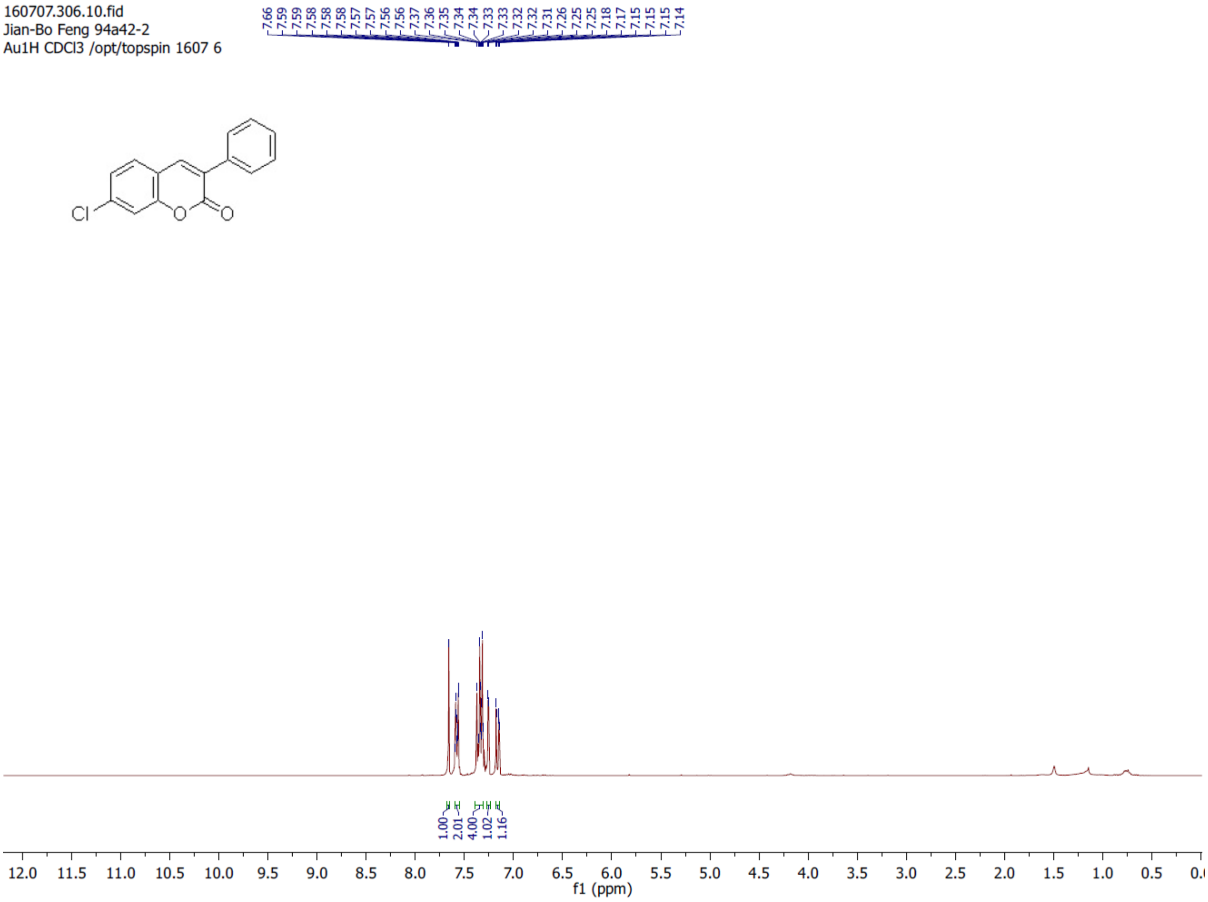

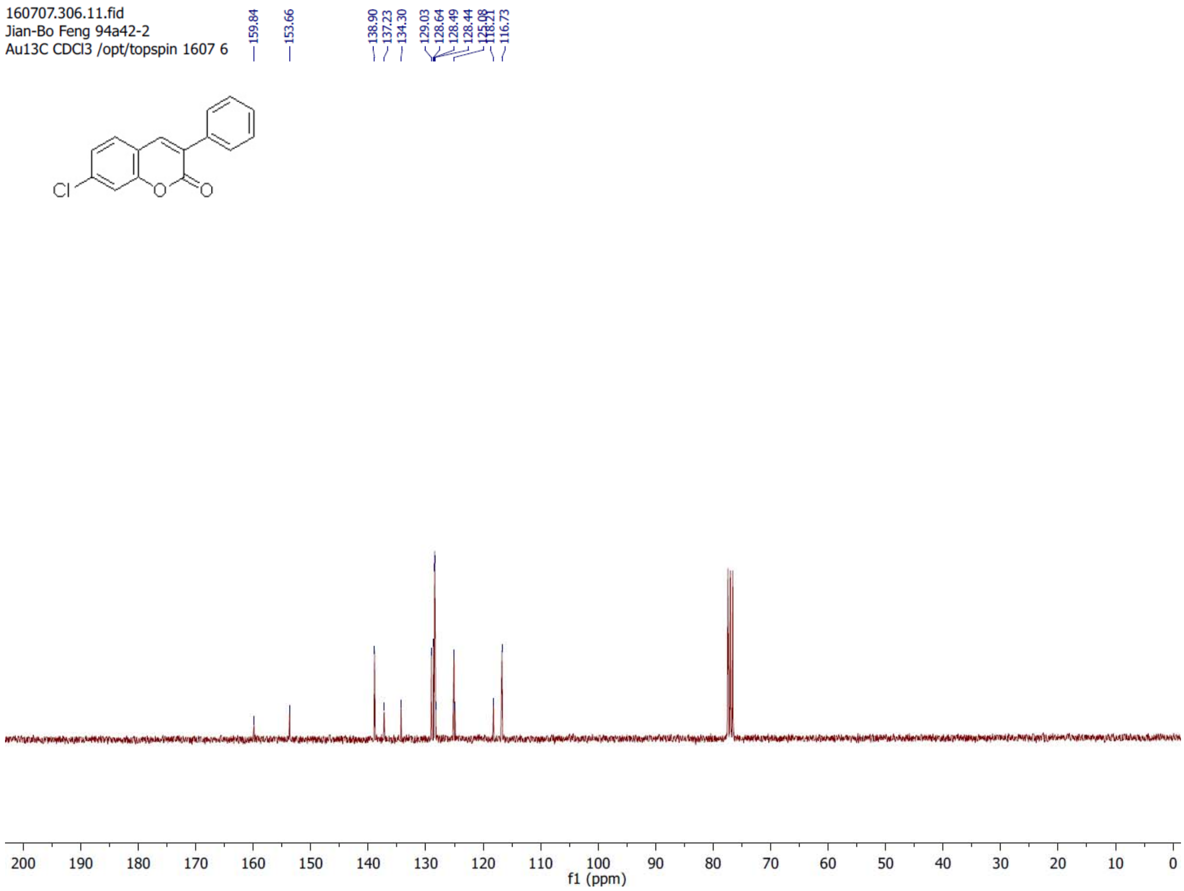

160707.306.11.fid
Jian-Bo Feng 94a42-2
Au13C CDCl3 /opt/topspin 1607 6
159.84
153.66
138.90
137.23
134.30
129.03
128.64
128.49
128.44
125.08
118.21
116.73
Cl
O
O
200 190 180 170 160 150 140 130 120 110 100 90 80 70 60 50 40 30 20 10 0
f1 (ppm)


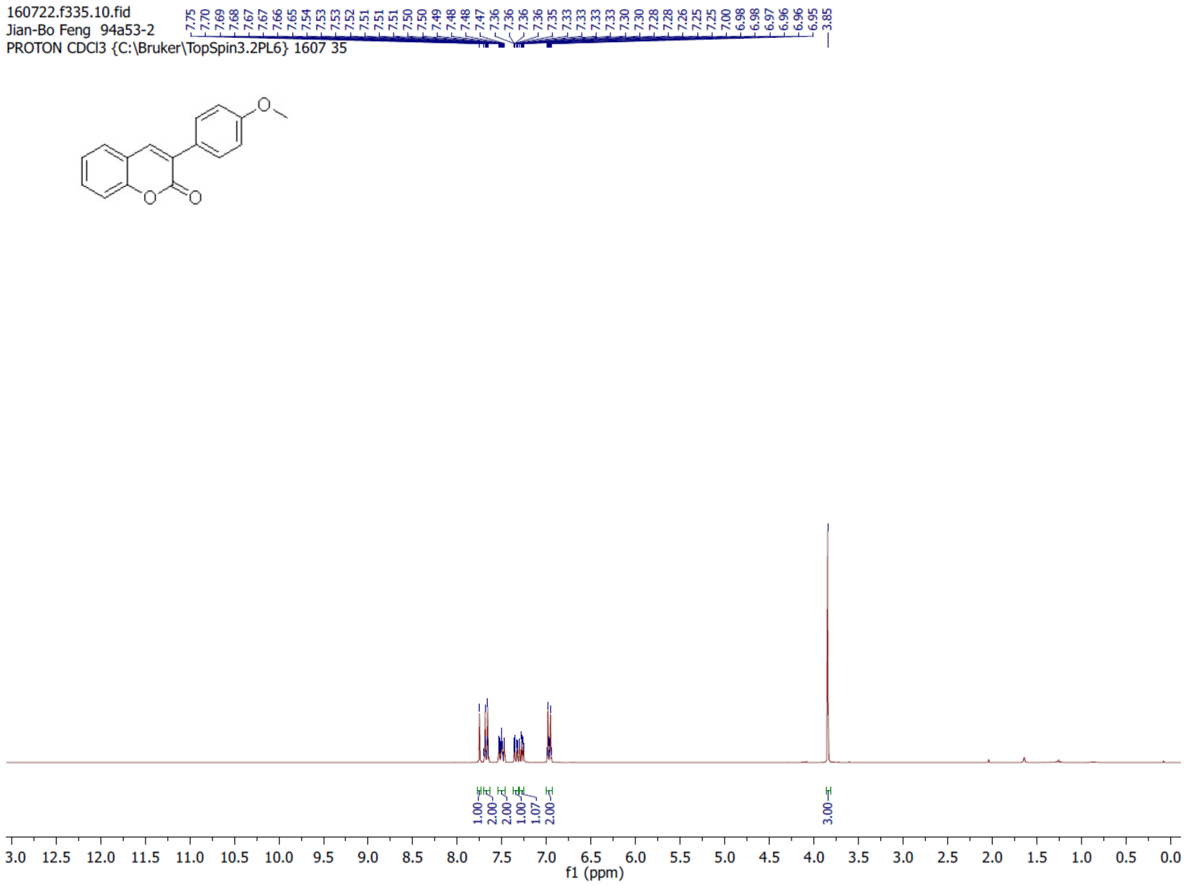

160722.f335.10.fid
Jian-Bo Feng 94a53-2
PROTON CDCl3 {C:\Bruker\TopSpin3.2PL6} 1607 35
7.75 7.70 7.69 7.68 7.67 7.66 7.65 7.54 7.53 7.53 7.52 7.51 7.51 7.50 7.49 7.48 7.48 7.47 7.36 7.36 7.36 7.35 7.33 7.33 7.33 7.30 7.28 7.26 7.25 7.00 6.98 6.98 6.97 6.96 6.96 6.95 3.85
O
O
O
1.00
2.00
2.00
1.00
1.07
2.00
3.00
3.0 12.5 12.0 11.5 11.0 10.5 10.0 9.5 9.0 8.5 8.0 7.5 7.0 6.5 6.0 5.5 5.0 4.5 4.0 3.5 3.0 2.5 2.0 1.5 1.0 0.5 0.0
f1 (ppm)

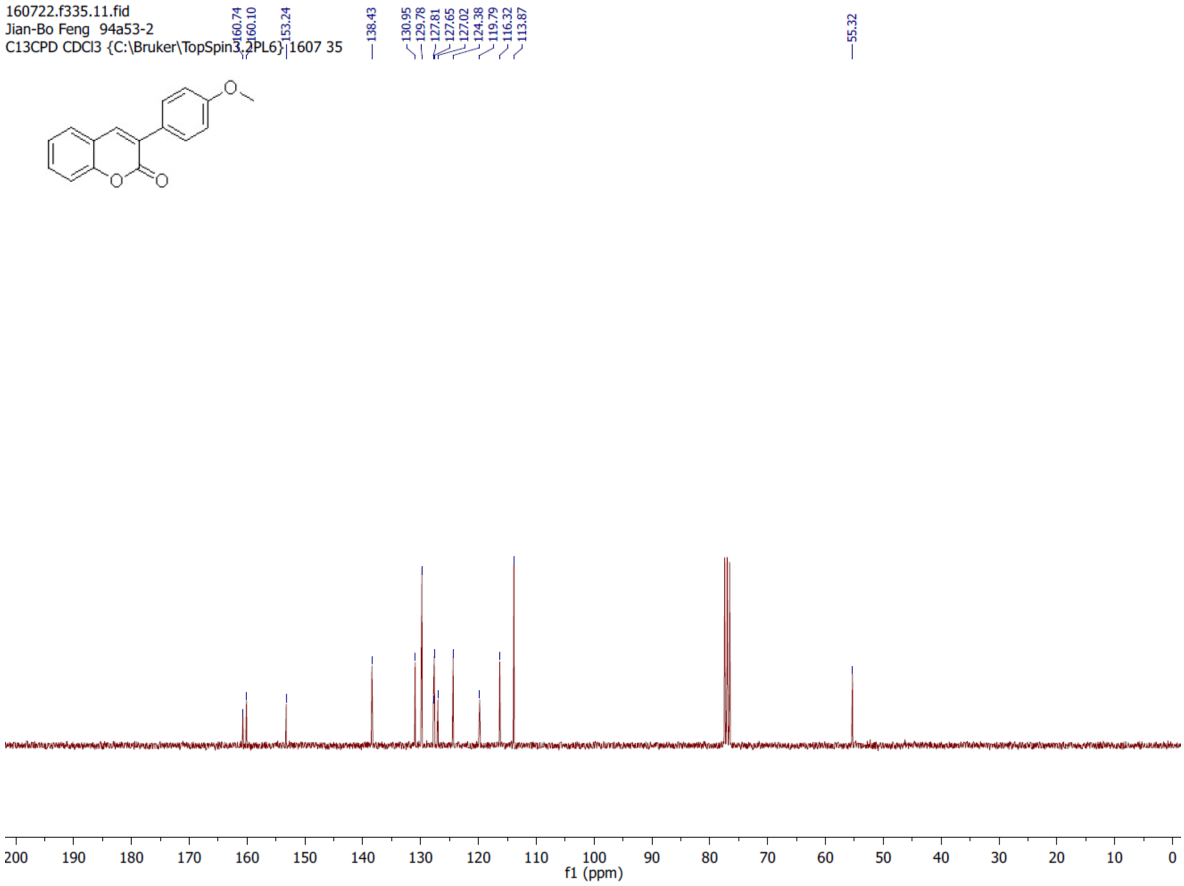

160722.f335.11.fid
Jian-Bo Feng 94a53-2
C13CPD CDCl3 {C:\Bruker\TopSpin3.2PL6} 1607 35
160.74
160.10
153.24
138.43
130.95
129.78
127.81
127.65
127.02
124.38
119.79
116.32
113.87
55.32
O
O
O
200 190 180 170 160 150 140 130 120 110 100 90 80 70 60 50 40 30 20 10 0
f1 (ppm)


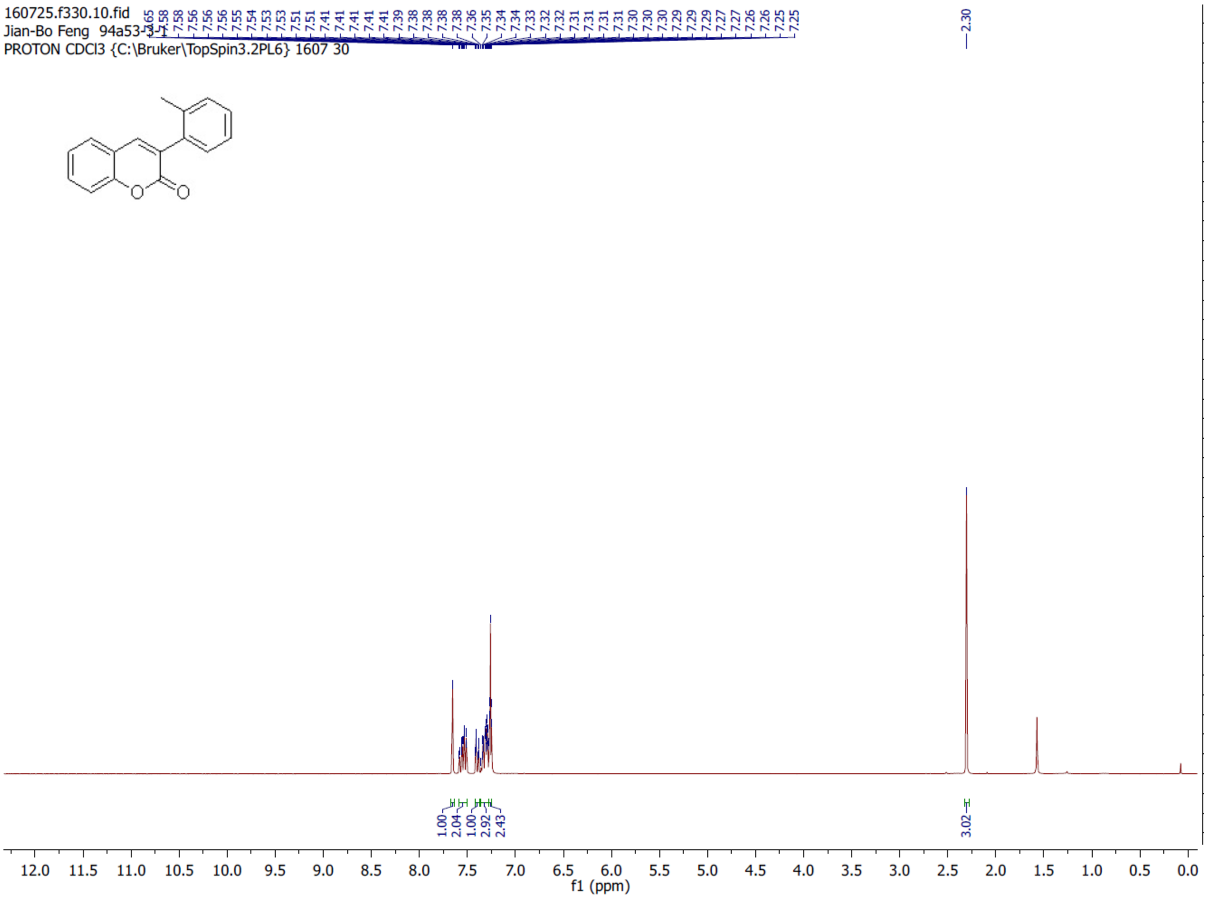

160725.f330.10.fid
Jian-Bo Feng 94a53-3-1
PROTON CDCl3 {C:\Bruker\TopSpin3.2PL6} 1607 30
7.65
7.58
7.56
7.56
7.56
7.55
7.54
7.53
7.53
7.51
7.51
7.41
7.41
7.41
7.41
7.39
7.38
7.38
7.38
7.38
7.36
7.35
7.34
7.34
7.33
7.32
7.32
7.31
7.31
7.31
7.30
7.30
7.30
7.29
7.29
7.27
7.26
7.25
7.25
2.30
O
O
1.00
2.04
1.00
2.92
2.43
3.02
12.0 11.5 11.0 10.5 10.0 9.5 9.0 8.5 8.0 7.5 7.0 6.5 6.0 5.5 5.0 4.5 4.0 3.5 3.0 2.5 2.0 1.5 1.0 0.5 0.0
f1 (ppm)

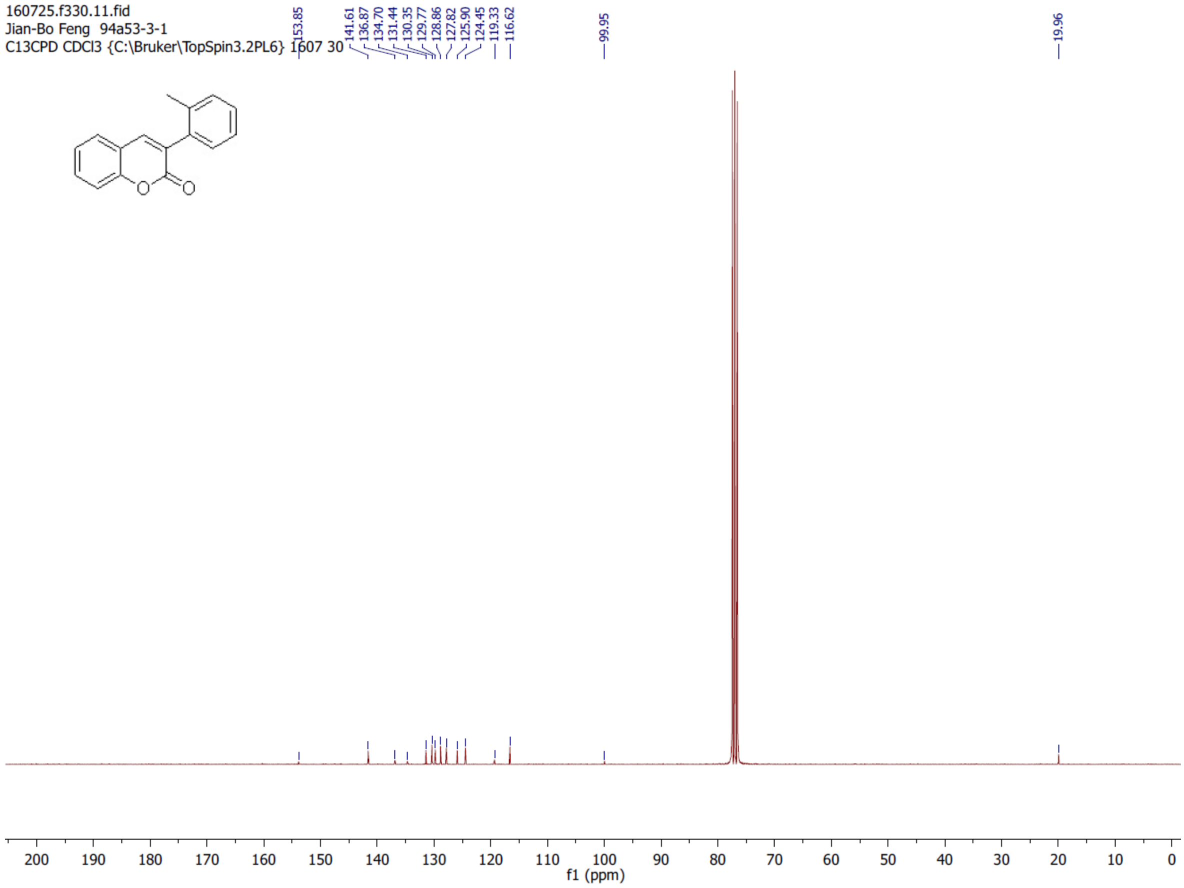
160725.f330.11.fid
Jian-Bo Feng 94a53-3-1
C13CPD CDCl3 {C:\Bruker\TopSpin3.2PL6} 1607 30
153.85
141.61
136.87
134.70
131.44
130.35
129.77
128.86
127.82
125.90
124.45
119.33
116.62
99.95
19.96
f1 (ppm)

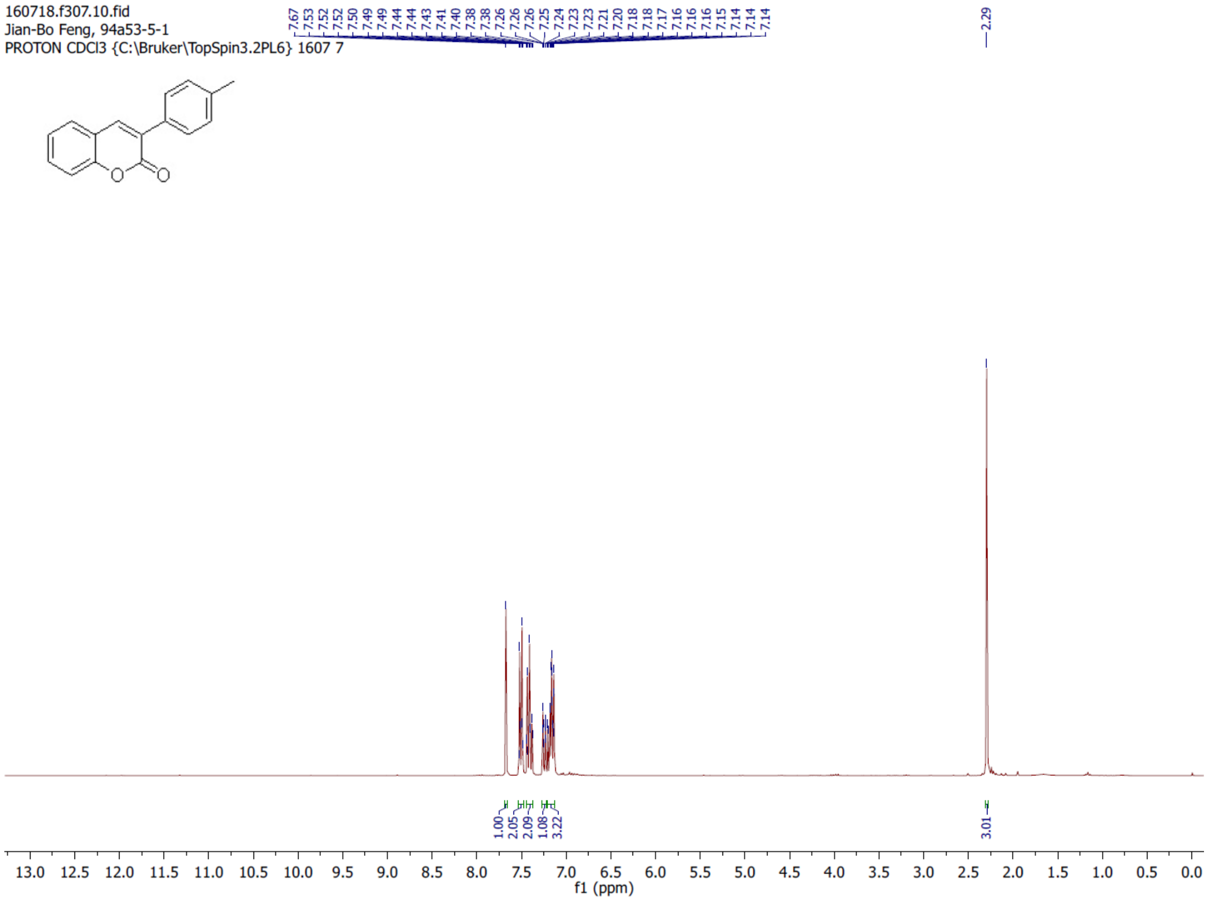
160718.f307.10.fid
Jian-Bo Feng, 94a53-5-1
PROTON CDCl3 {C:\Bruker\TopSpin3.2PL6} 1607 7
7.67
7.53
7.52
7.52
7.50
7.49
7.49
7.44
7.44
7.43
7.41
7.40
7.38
7.38
7.26
7.26
7.26
7.25
7.24
7.23
7.23
7.21
7.20
7.18
7.18
7.17
7.16
7.16
7.15
7.14
7.14
7.14
2.29
1.00
2.05
2.09
1.08
3.22
3.01
f1 (ppm)

160718.f307.11.fid
Jian-Bo Feng, 94a53-5-1
C13CPD CDCl3 {C:\Bruker\TopSpin3.2PL6} 1607 7

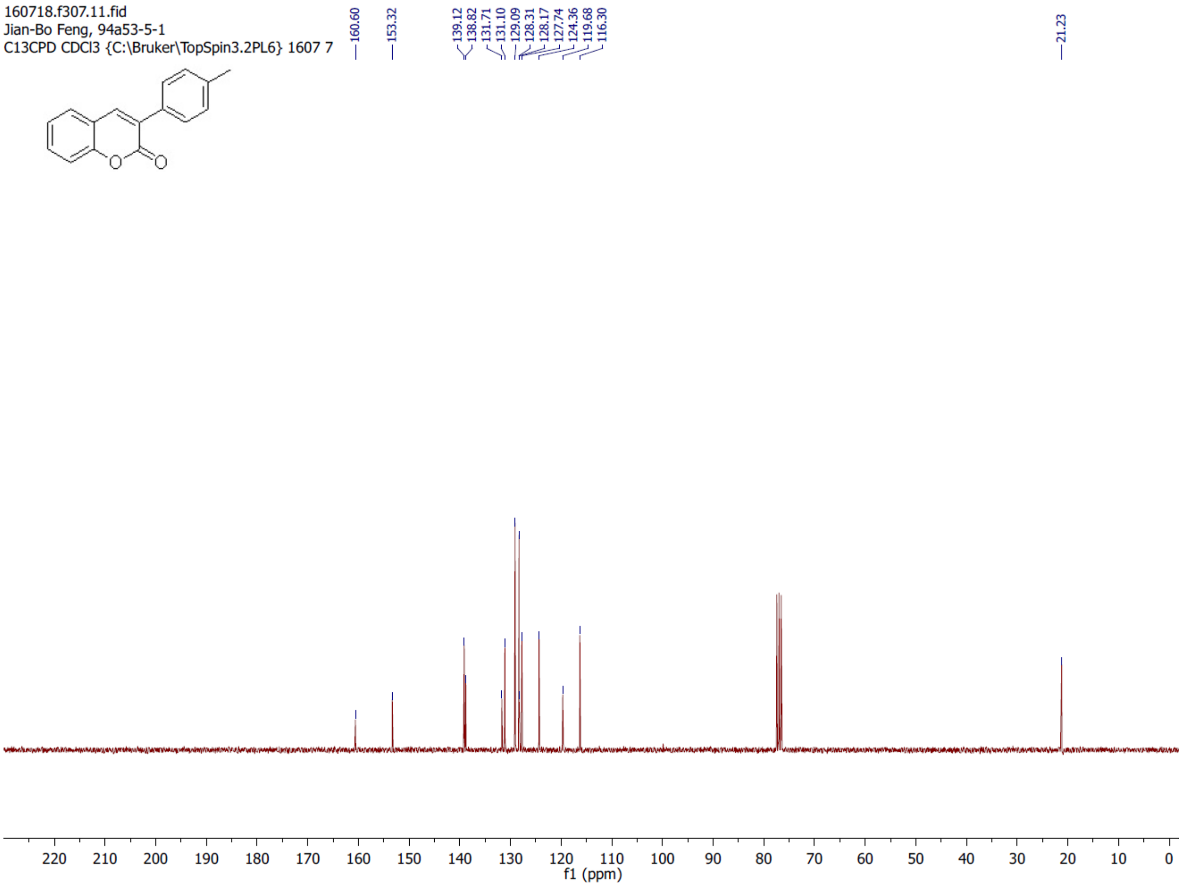


160718.f308.10.fid
Jian-Bo Feng, 94a53-6-1
PROTON CDCl3 {C:\Bruker\TopSpin3.2PL6} 1607 8

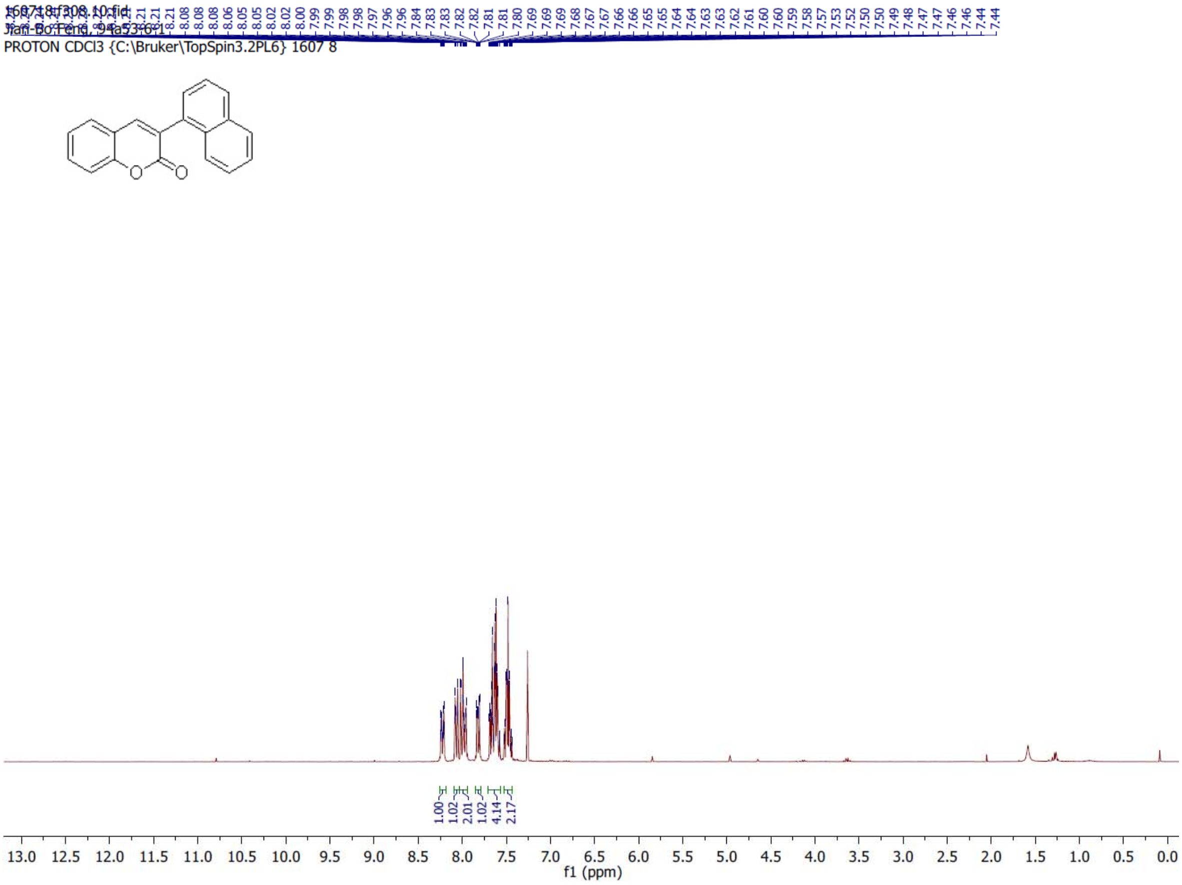

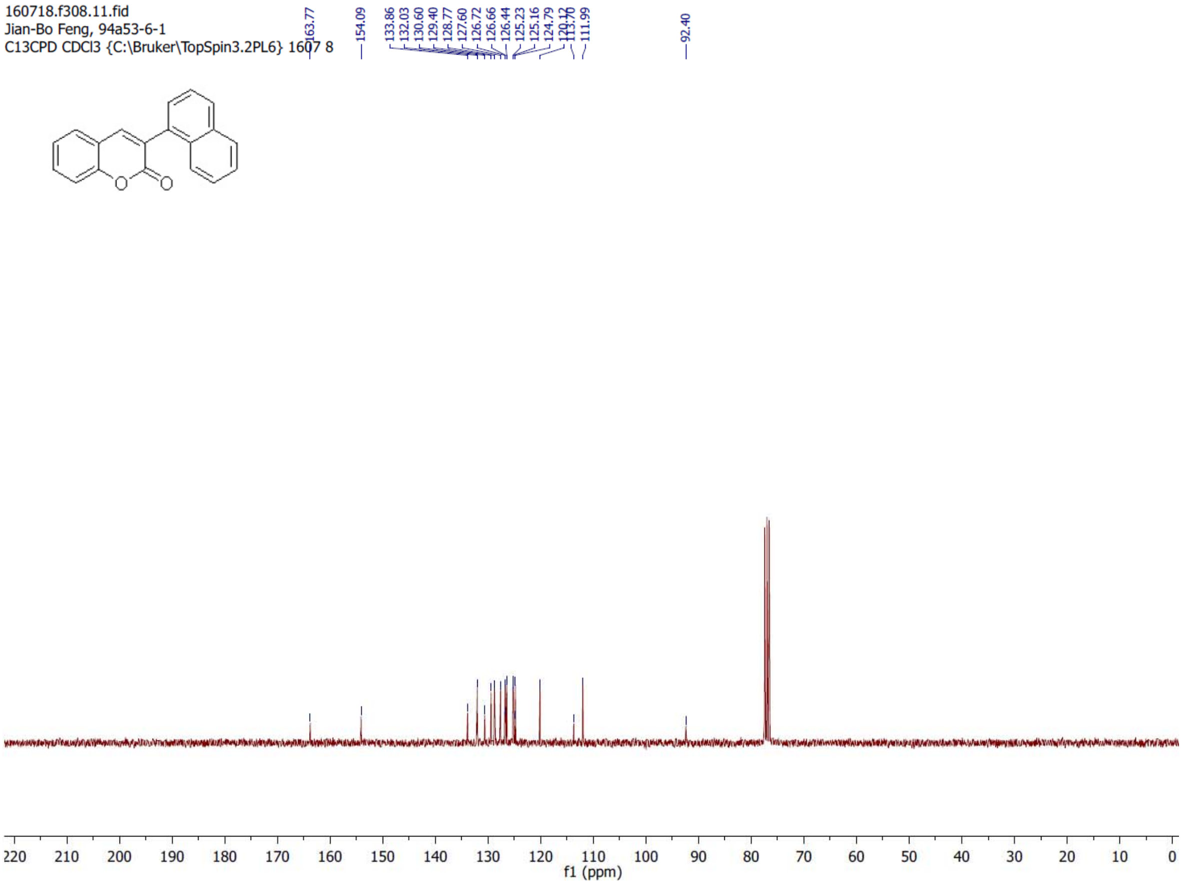
160718.f308.11.fid
Jian-Bo Feng, 94a53-6-1
C13CPD CDCl3 {C:\Bruker\TopSpin3.2PL6} 1607 8
163.77
154.09
133.86
132.03
130.60
129.40
128.77
127.60
126.72
126.66
126.44
125.23
125.16
124.79
120.12
113.70
111.99
92.40
220 210 200 190 180 170 160 150 140 130 120 110 100 90 80 70 60 50 40 30 20 10 0
f1 (ppm)

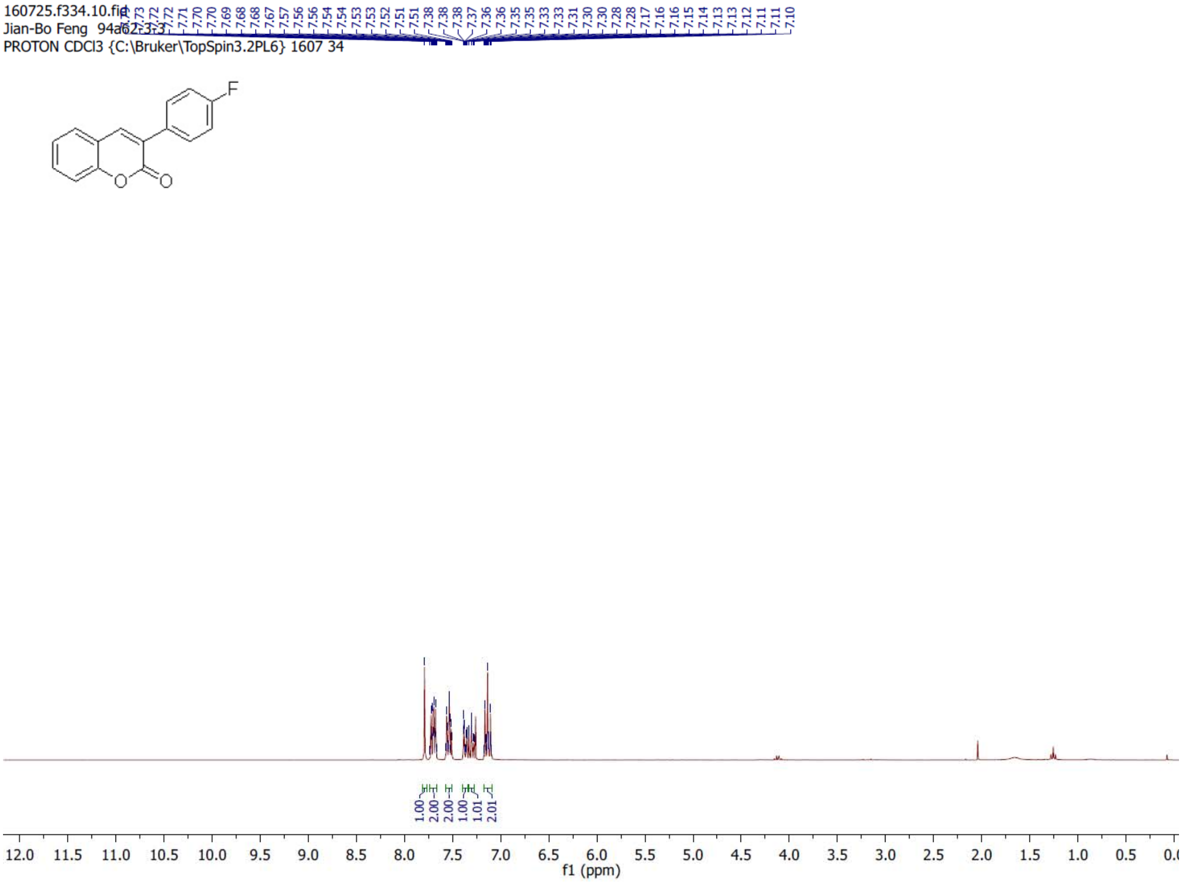
160725.f334.10.fid
Jian-Bo Feng 94a62-3-3
PROTON CDCl3 {C:\Bruker\TopSpin3.2PL6} 1607 34
7.73
7.72
7.72
7.71
7.70
7.70
7.69
7.68
7.68
7.67
7.57
7.56
7.56
7.54
7.54
7.53
7.53
7.52
7.51
7.51
7.38
7.38
7.38
7.37
7.36
7.36
7.35
7.35
7.33
7.33
7.31
7.30
7.30
7.28
7.28
7.17
7.16
7.16
7.15
7.14
7.13
7.13
7.12
7.11
7.11
7.10
F
1.00
2.00
2.00
1.00
1.01
2.01
12.0 11.5 11.0 10.5 10.0 9.5 9.0 8.5 8.0 7.5 7.0 6.5 6.0 5.5 5.0 4.5 4.0 3.5 3.0 2.5 2.0 1.5 1.0 0.5 0.0
f1 (ppm)

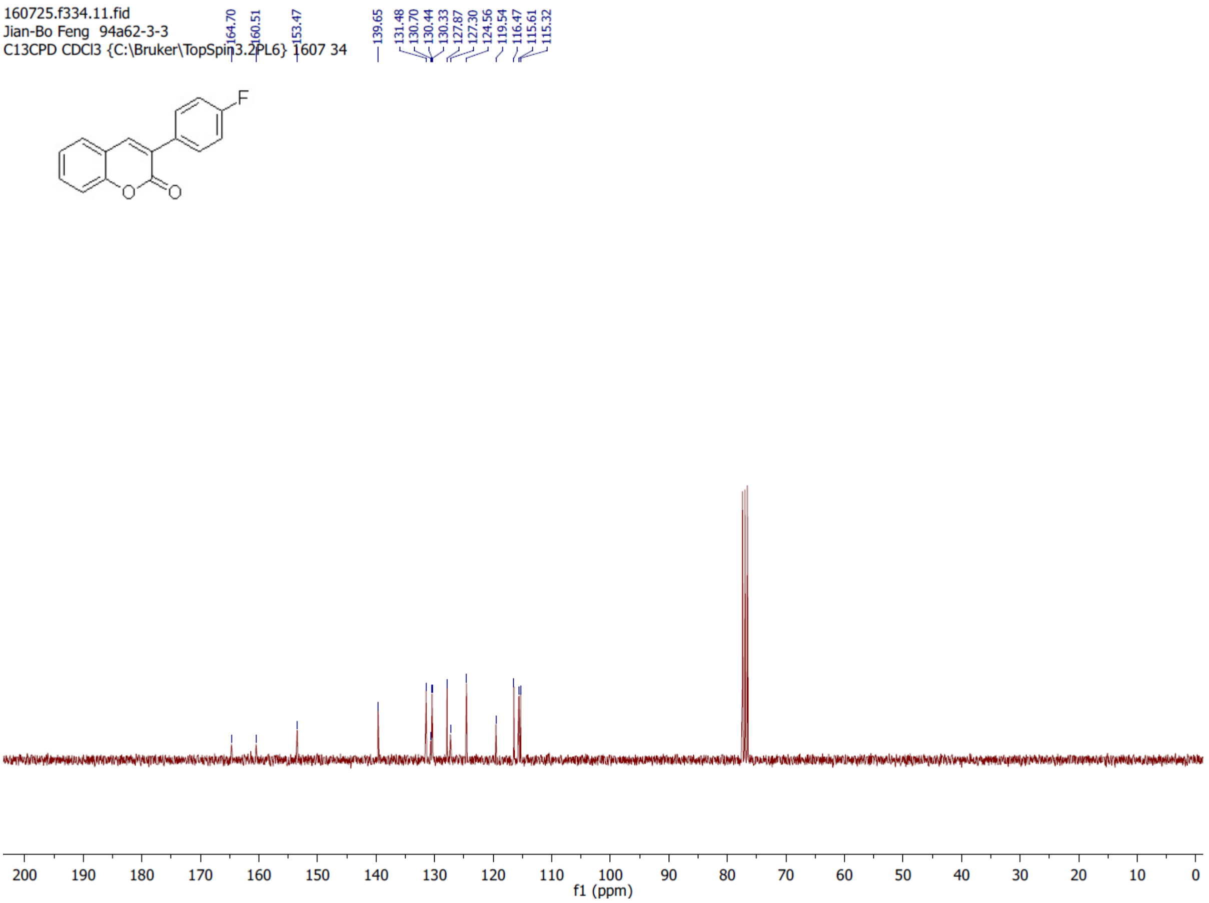
160725.f334.11.fid
Jian-Bo Feng 94a62-3-3
C13CPD CDCl3 {C:\Bruker\TopSpin3.2PL6} 1607 34
164.70
160.51
153.47
139.65
131.48
130.70
130.44
130.33
127.87
127.30
124.56
119.54
116.47
115.61
115.32
F
O
O
200 190 180 170 160 150 140 130 120 110 100 90 80 70 60 50 40 30 20 10 0
f1 (ppm)

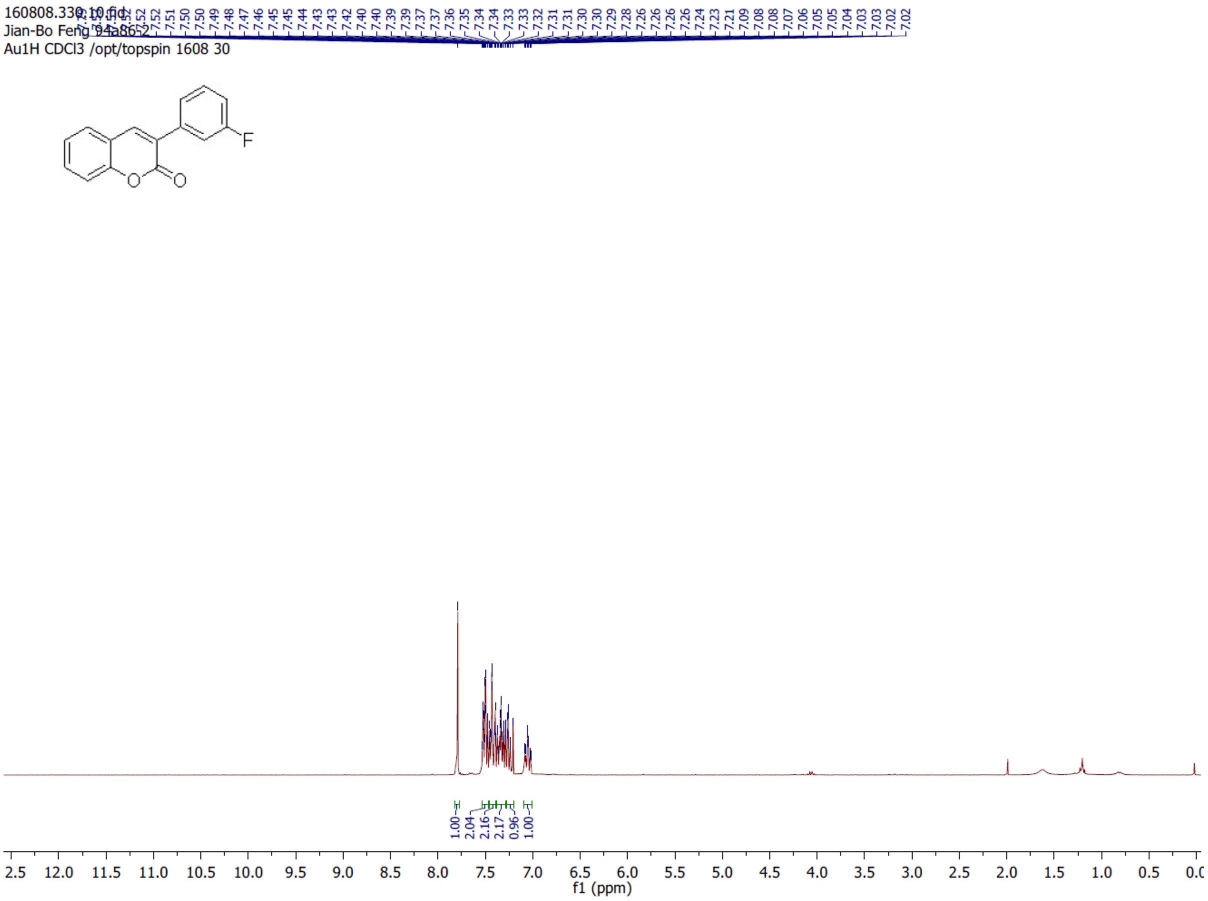
160808.330.10.fid
Jian-Bo Feng 94a86-2
Au1H CDCl3 /opt/topspin 1608 30
7.52
7.52
7.51
7.50
7.50
7.49
7.48
7.47
7.46
7.45
7.45
7.44
7.43
7.43
7.42
7.40
7.40
7.39
7.39
7.37
7.37
7.36
7.35
7.34
7.34
7.33
7.33
7.32
7.31
7.31
7.30
7.30
7.29
7.28
7.26
7.26
7.26
7.26
7.24
7.23
7.21
7.09
7.08
7.08
7.07
7.06
7.05
7.05
7.04
7.03
7.03
7.02
7.02
F
O
O
1.00
2.04
2.16
2.17
0.96
1.00
12.0 11.5 11.0 10.5 10.0 9.5 9.0 8.5 8.0 7.5 7.0 6.5 6.0 5.5 5.0 4.5 4.0 3.5 3.0 2.5 2.0 1.5 1.0 0.5
f1 (ppm)

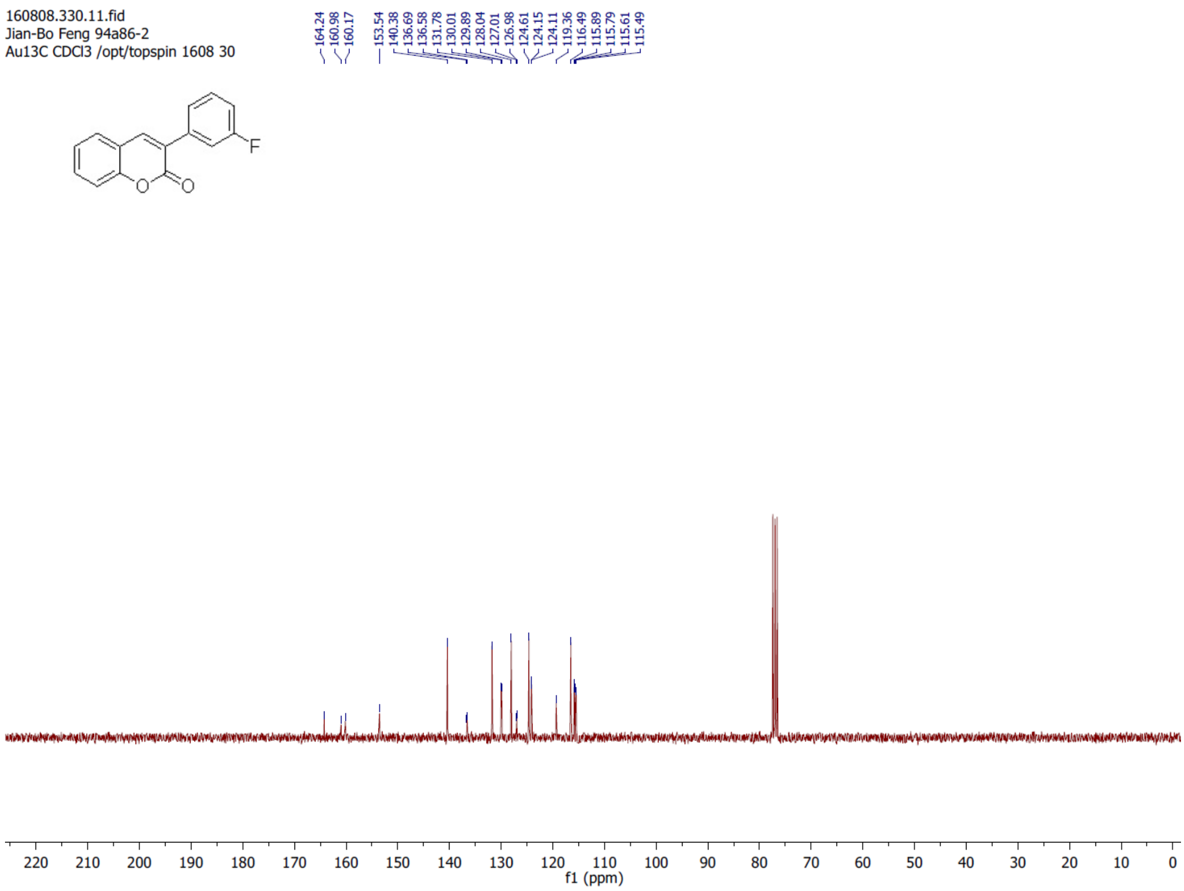
160808.330.11.fid
Jian-Bo Feng 94a86-2
Au13C CDCl3 /opt/topspin 1608 30
164.24
160.98
160.17
153.54
140.38
136.69
136.58
131.78
130.01
129.89
128.04
127.01
126.98
124.61
124.15
124.11
119.36
116.49
115.89
115.79
115.61
115.49
F
O
O
220
210
200
190
180
170
160
150
140
130
120
110
100
90
80
70
60
50
40
30
20
10
0
f1 (ppm)

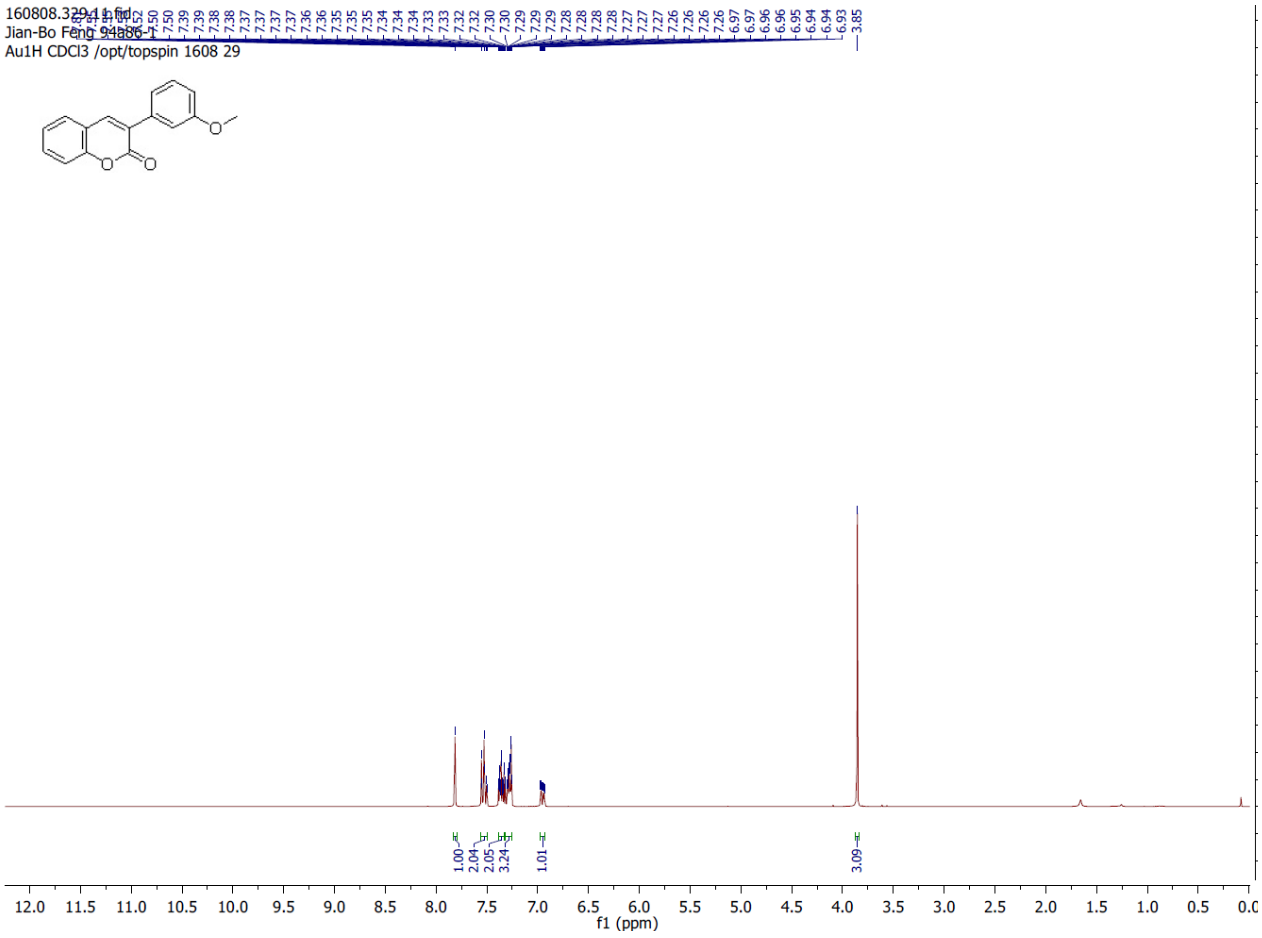
160808.329.11.fid
Jian-Bo Feng 94a86-1
Au1H CDCl3 /opt/topspin 1608 29
7.52
7.50
7.50
7.39
7.39
7.38
7.38
7.37
7.37
7.37
7.37
7.36
7.36
7.35
7.35
7.35
7.34
7.34
7.34
7.33
7.33
7.32
7.32
7.30
7.30
7.29
7.29
7.29
7.28
7.28
7.28
7.28
7.27
7.27
7.27
7.26
7.26
7.26
7.26
6.97
6.97
6.96
6.96
6.95
6.94
6.94
6.93
3.85
O
O
O
1.00
2.04
2.05
3.24
1.01
3.09
12.0
11.5
11.0
10.5
10.0
9.5
9.0
8.5
8.0
7.5
7.0
6.5
6.0
5.5
5.0
4.5
4.0
3.5
3.0
2.5
2.0
1.5
1.0
0.5
0.0
f1 (ppm)

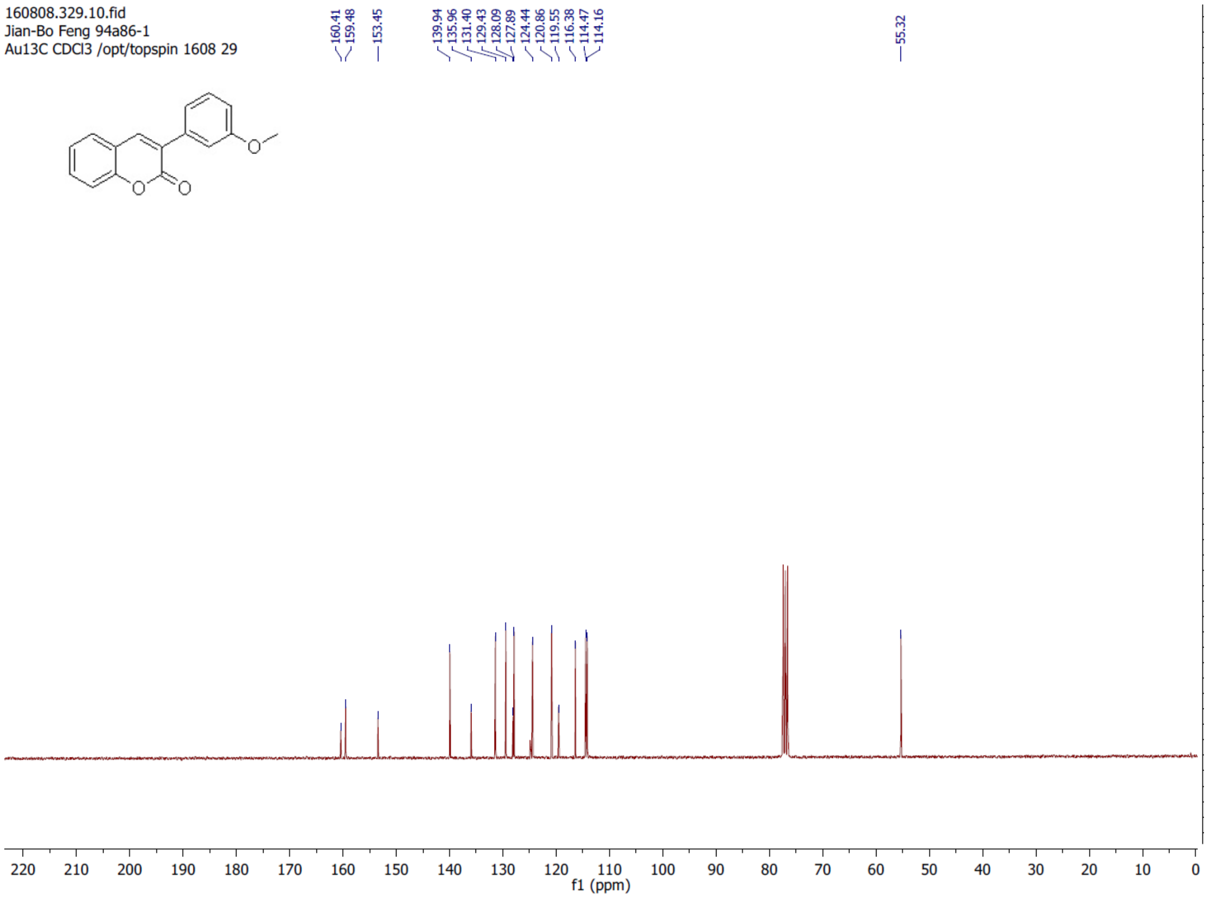
160808.329.10.fid
Jian-Bo Feng 94a86-1
Au13C CDCl3 /opt/topspin 1608 29
160.41
159.48
153.45
139.94
135.96
131.40
129.43
128.09
127.89
124.44
120.86
119.55
116.38
114.47
114.16
55.32
f1 (ppm)

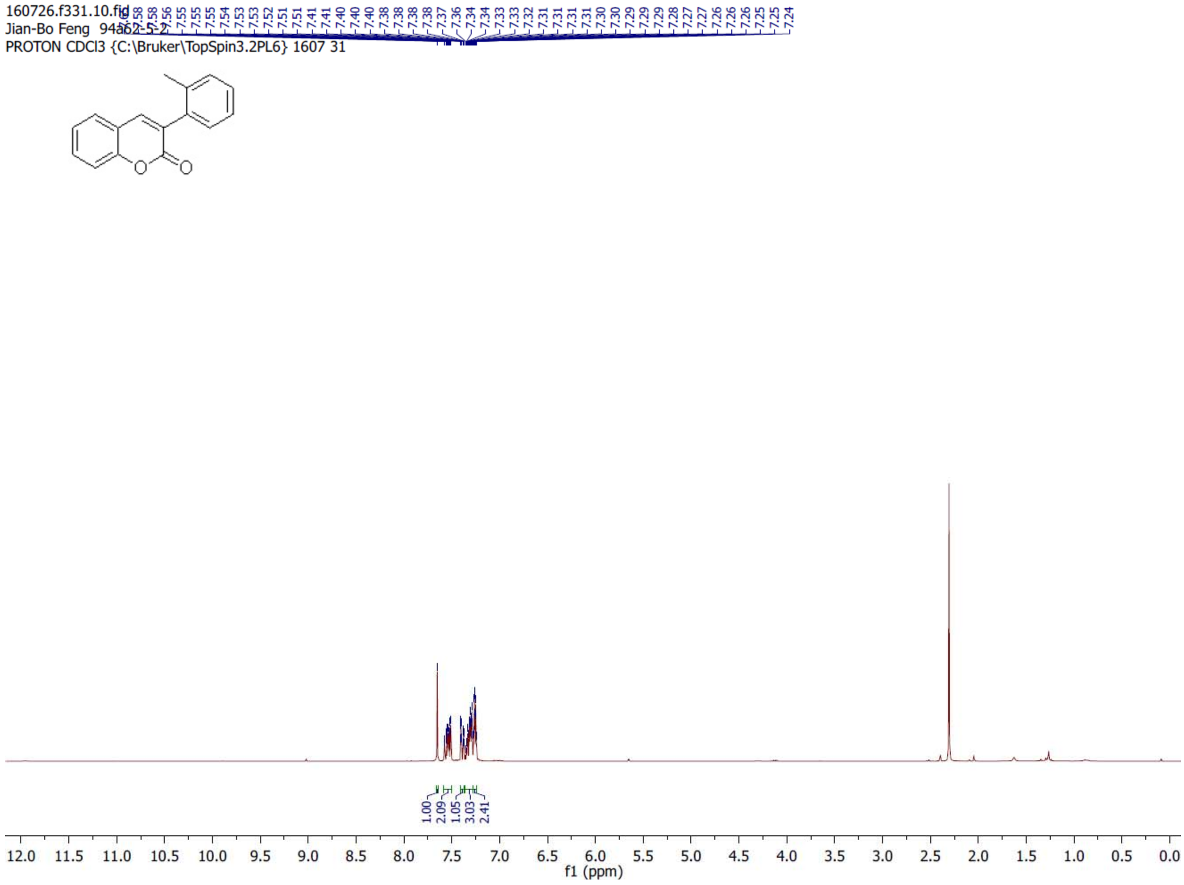
160726.f331.10.fid
Jian-Bo Feng 94a62-5-2
PROTON CDCl3 {C:\Bruker\TopSpin3.2PL6} 1607 31
7.58
7.56
7.55
7.55
7.55
7.54
7.53
7.53
7.52
7.51
7.51
7.41
7.41
7.40
7.40
7.38
7.38
7.38
7.38
7.37
7.36
7.34
7.34
7.33
7.33
7.32
7.31
7.31
7.31
7.30
7.30
7.29
7.29
7.29
7.28
7.27
7.27
7.26
7.26
7.25
7.25
7.24
1.00
2.09
1.05
3.03
2.41
f1 (ppm)

160726.f331.11.fid
Jian-Bo Feng 94a62-5-2
C13CPD CDCl3 {C:\Bruker\TopSpin3.2PL6} 1607 31

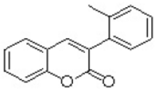

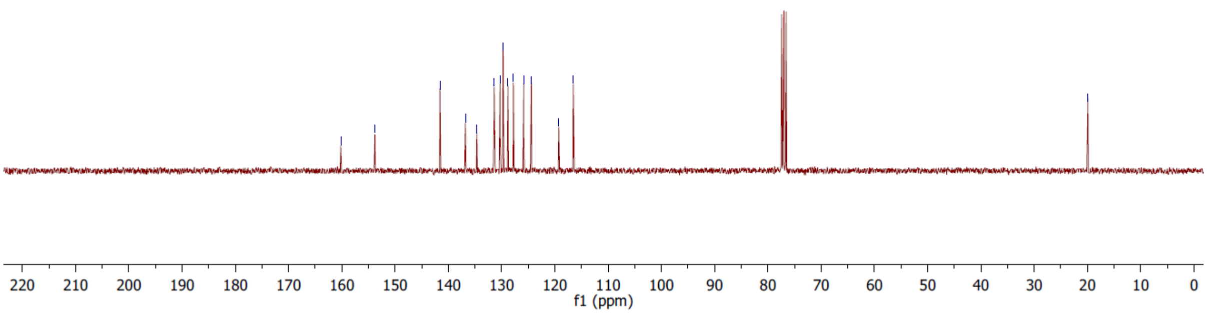

160728.f326.10.fid
Jian-Bo Feng 94a69-1-2
PROTON CDCl3 {C:\Bruker\TopSpin3.2PL6} 1607 26

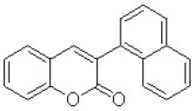

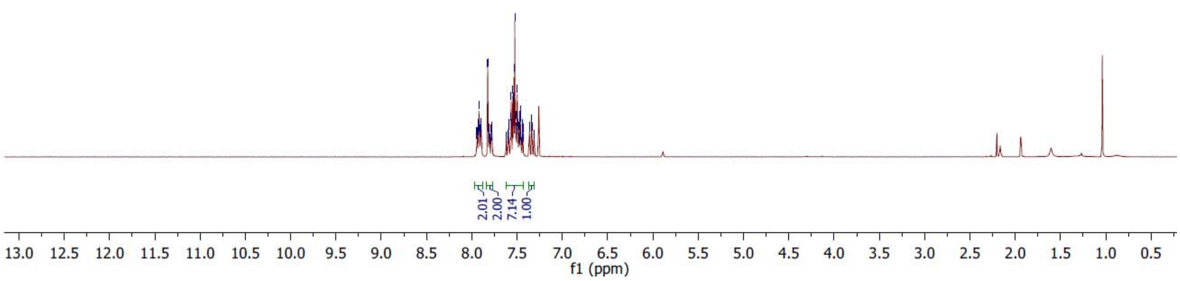


160728.f326.11.fid
Jian-Bo Feng 94a69-1-2
C13CPD CDCl3 {C:\Bruker\TopSpin3.2PL6} 1607 26

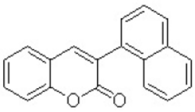

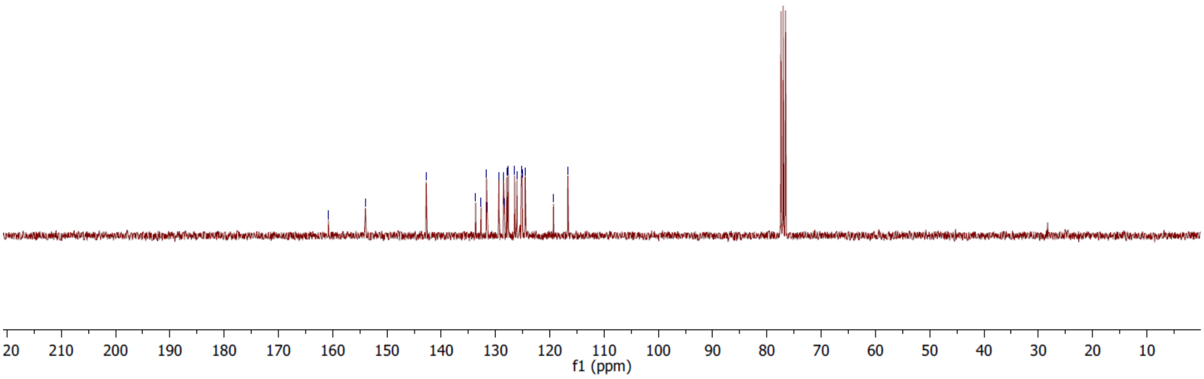

160727.f336.10.fid
Jian-Bo Feng 94a69-2-2
PROTON CDCl3 {C:\Bruker\TopSpin3.2PL6} 1607 36

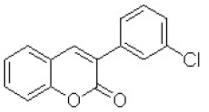


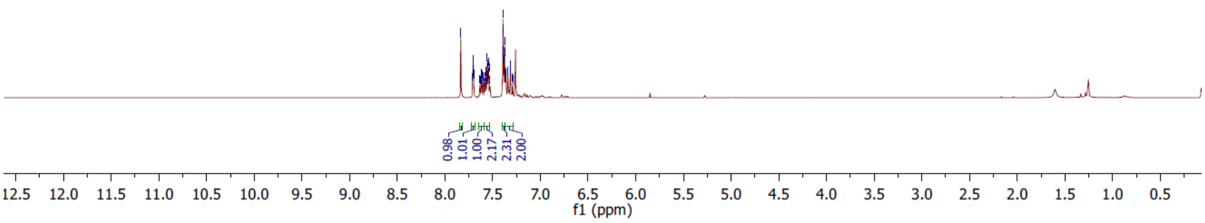


160727.f336.11.fid
Jian-Bo Feng 94a69-2-2
C13CPD CDCl3 {C:\Bruker\TopSpin3.2PL6} 1607 36

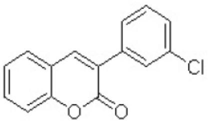


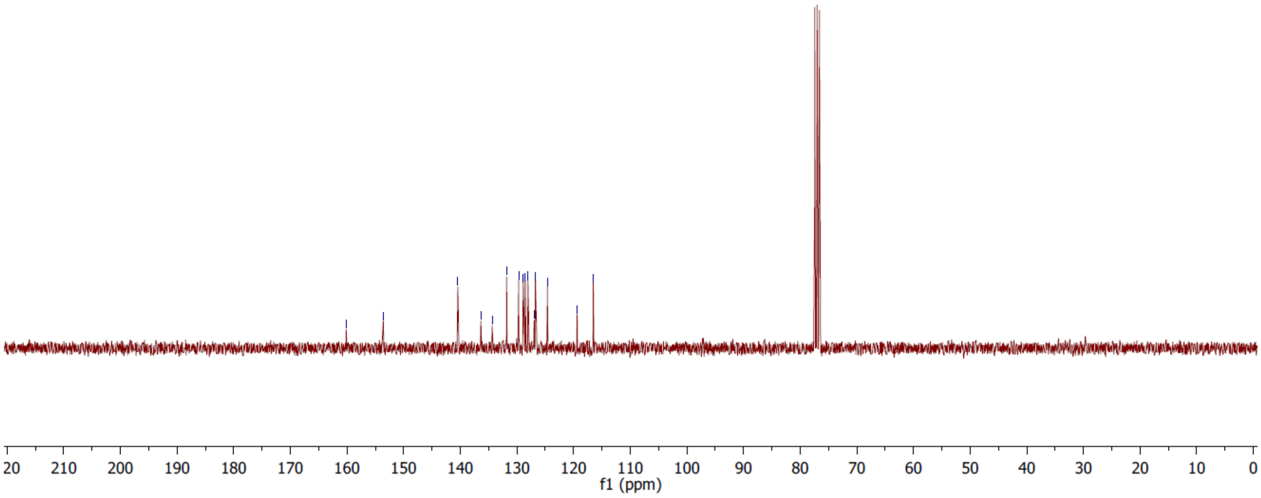

160906.320.11.fid
Feng/ 94a125-1
Au1H CDCl3 /opt/topspin 1609 20

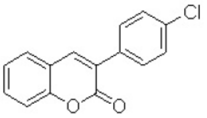


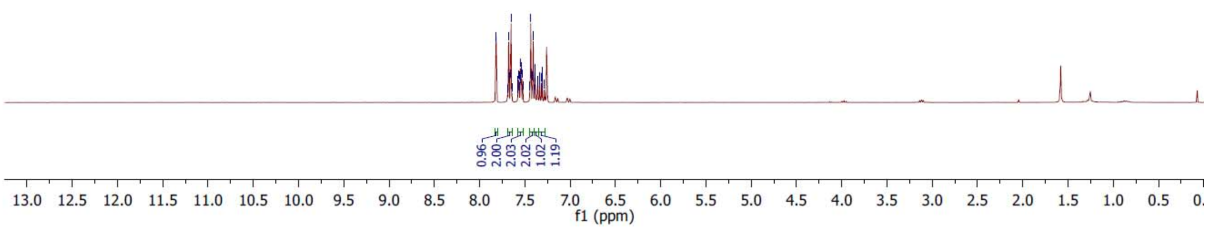


160906.320.10.fid
Feng/ 94a125-1
Au13C CDCl3 /opt/topspin 1609 20

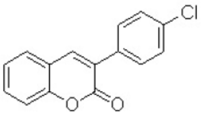


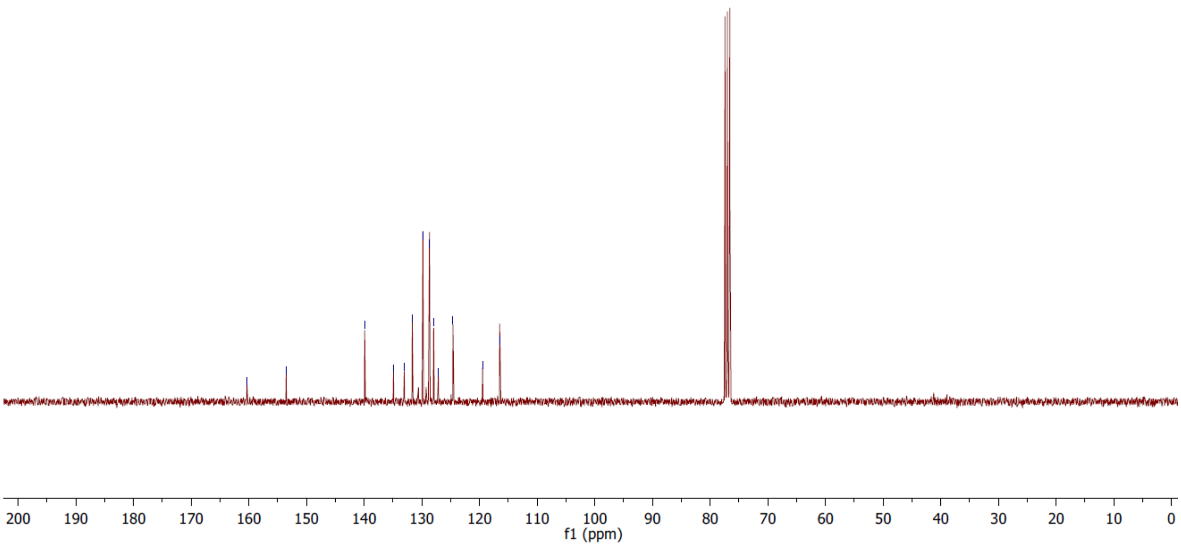

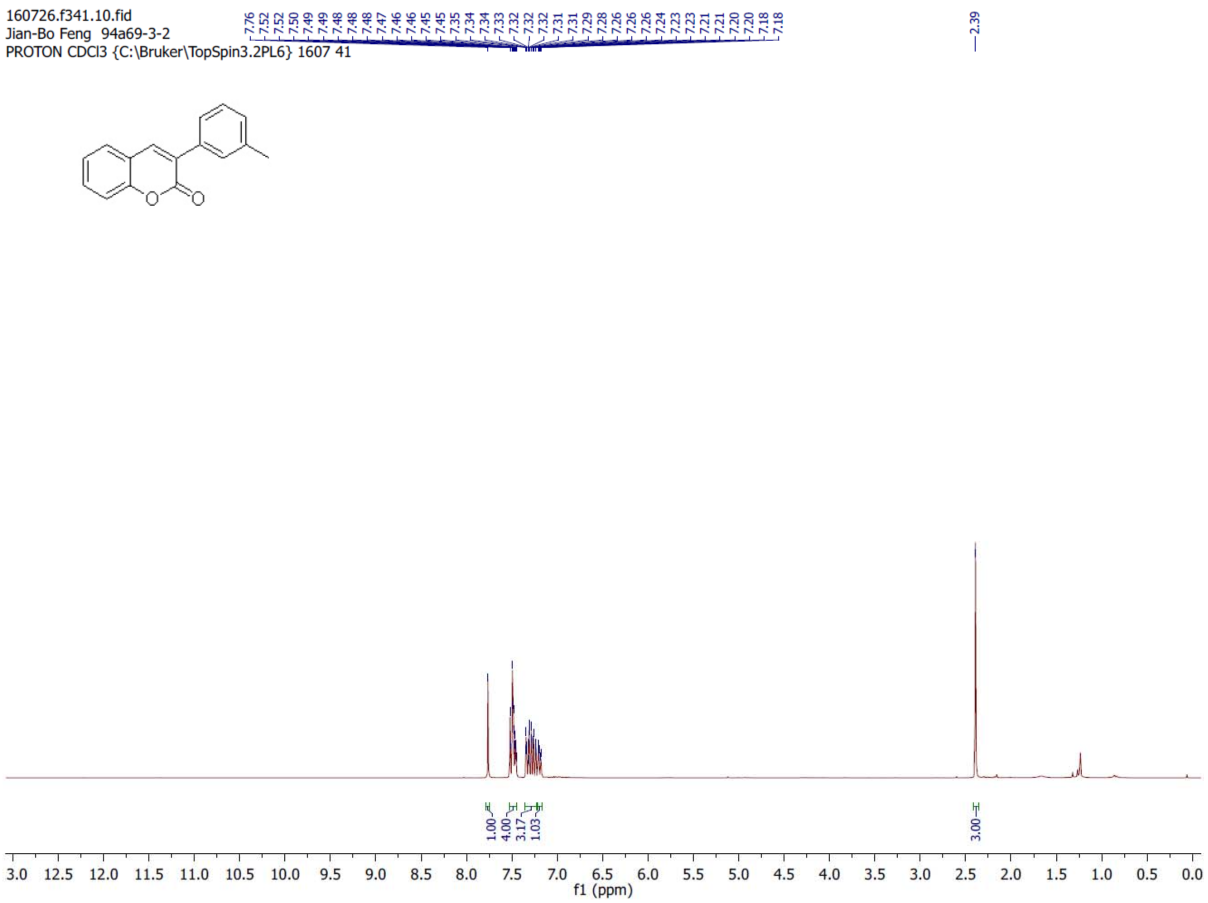
160726.f341.10.fid
Jian-Bo Feng 94a69-3-2
PROTON CDCl3 {C:\Bruker\TopSpin3.2PL6} 1607 41
7.76 7.52 7.52 7.50 7.49 7.49 7.48 7.48 7.47 7.46 7.46 7.45 7.45 7.35 7.34 7.34 7.33 7.32 7.32 7.32 7.31 7.31 7.29 7.28 7.26 7.26 7.24 7.23 7.23 7.21 7.21 7.20 7.20 7.18 7.18
2.39
1.00 4.00 3.17 1.03
3.00
f1 (ppm)

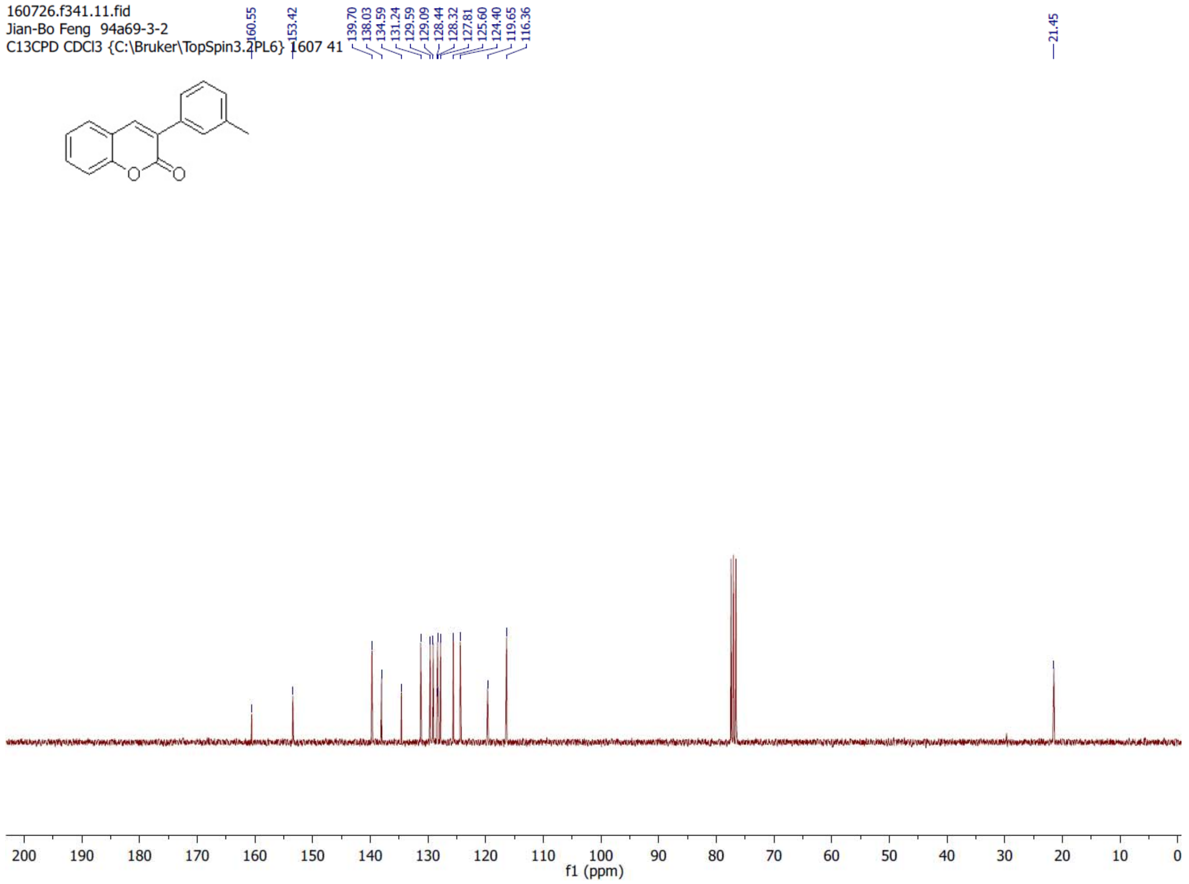
160726.f341.11.fid
Jian-Bo Feng 94a69-3-2
C13CPD CDCl3 {C:\Bruker\TopSpin3.2PL6} 1607 41
160.55 153.42 139.70 138.03 134.59 131.24 129.59 129.09 128.44 128.32 127.81 125.60 124.40 119.65 116.36
21.45
f1 (ppm)

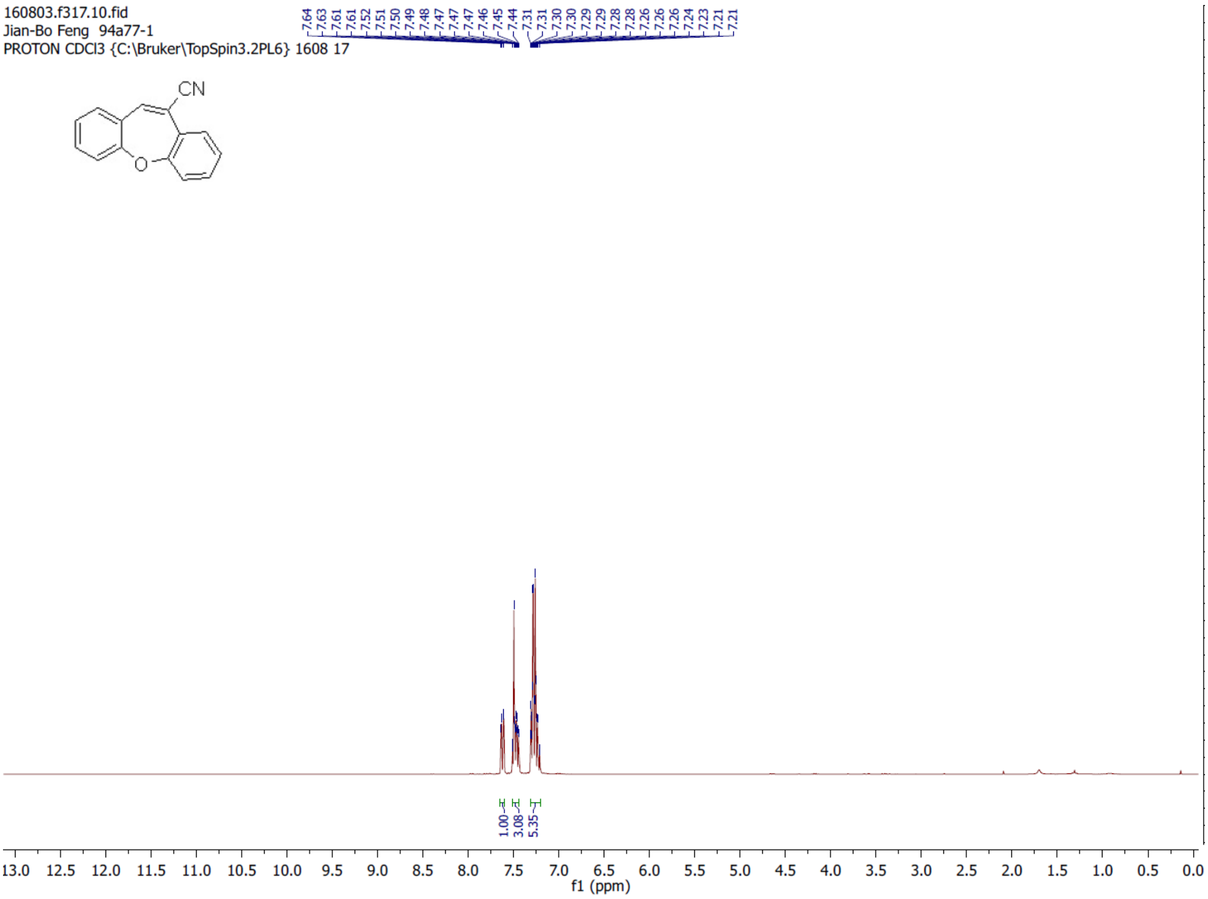
160803.f317.10.fid
Jian-Bo Feng 94a77-1
PROTON CDCl3 {C:\Bruker\TopSpin3.2PL6} 1608 17
CN
O
7.64
7.63
7.61
7.61
7.52
7.51
7.50
7.49
7.48
7.47
7.47
7.47
7.46
7.45
7.44
7.31
7.31
7.30
7.30
7.29
7.29
7.28
7.28
7.26
7.26
7.26
7.24
7.23
7.21
7.21
1.00
3.08
5.35
13.0 12.5 12.0 11.5 11.0 10.5 10.0 9.5 9.0 8.5 8.0 7.5 7.0 6.5 6.0 5.5 5.0 4.5 4.0 3.5 3.0 2.5 2.0 1.5 1.0 0.5 0.0
f1 (ppm)

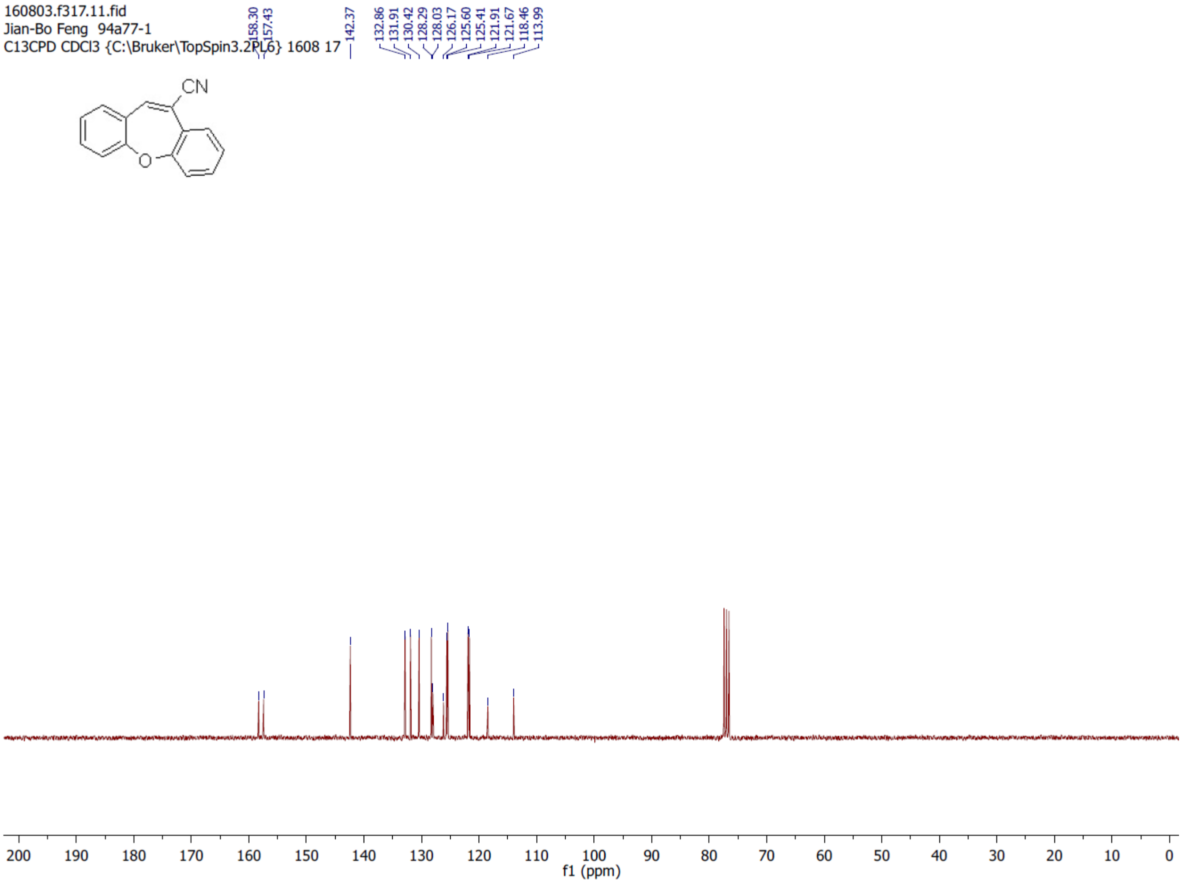
160803.f317.11.fid
Jian-Bo Feng 94a77-1
C13CPD CDCl3 {C:\Bruker\TopSpin3.2PL6} 1608 17
CN
O
158.30
157.43
142.37
132.86
131.91
130.42
128.29
128.03
126.17
125.60
125.41
121.91
121.67
118.46
113.99
200 190 180 170 160 150 140 130 120 110 100 90 80 70 60 50 40 30 20 10 0
f1 (ppm)

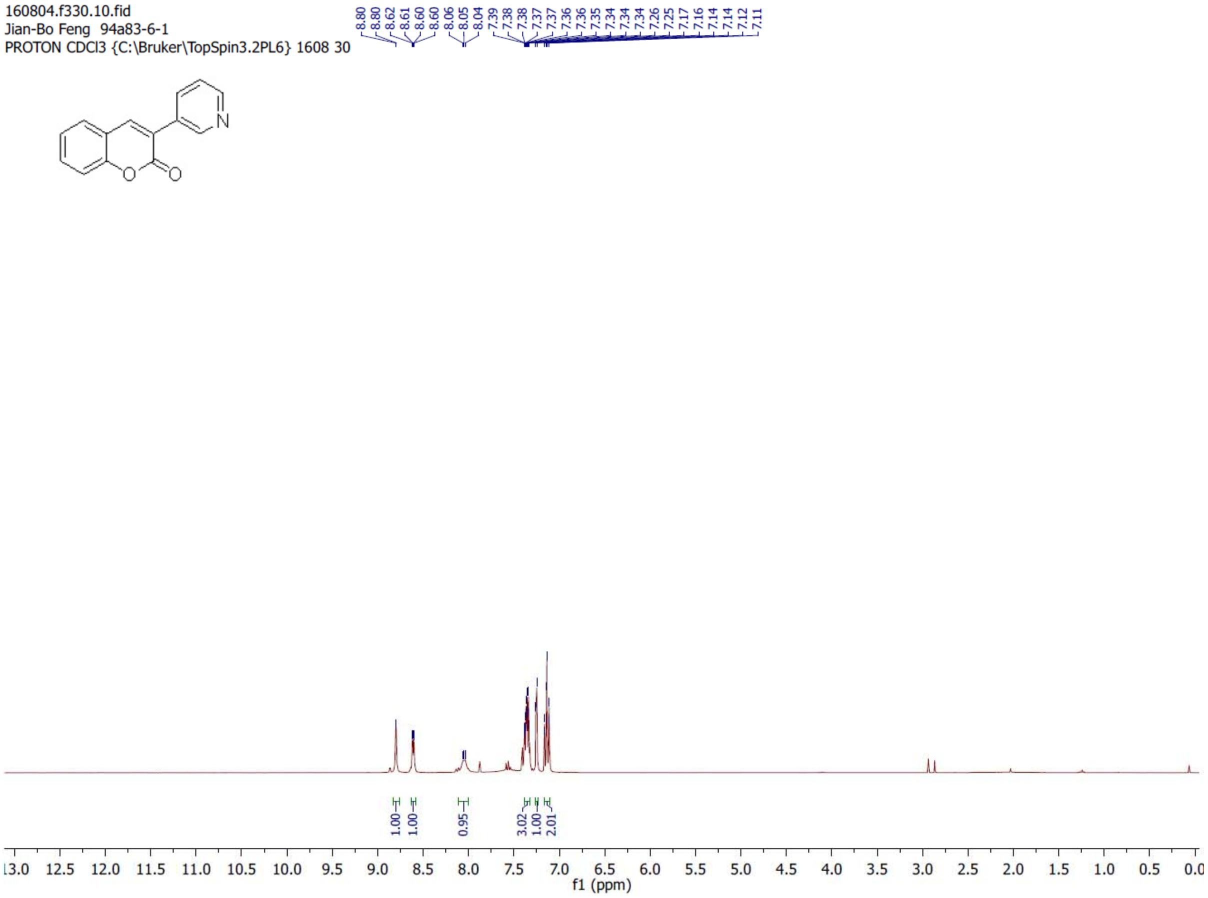

160804.f330.10.fid
Jian-Bo Feng 94a83-6-1
PROTON CDCl3 {C:\Bruker\TopSpin3.2PL6} 1608 30
8.80 8.80 8.62 8.61 8.60 8.60 8.06 8.05 8.04 7.39 7.38 7.38 7.37 7.37 7.36 7.36 7.35 7.34 7.34 7.34 7.34 7.26 7.25 7.17 7.16 7.14 7.14 7.12 7.11
N
O
O
1.00
1.00
0.95
3.02
1.00
2.01
13.0 12.5 12.0 11.5 11.0 10.5 10.0 9.5 9.0 8.5 8.0 7.5 7.0 6.5 6.0 5.5 5.0 4.5 4.0 3.5 3.0 2.5 2.0 1.5 1.0 0.5 0.0
f1 (ppm)


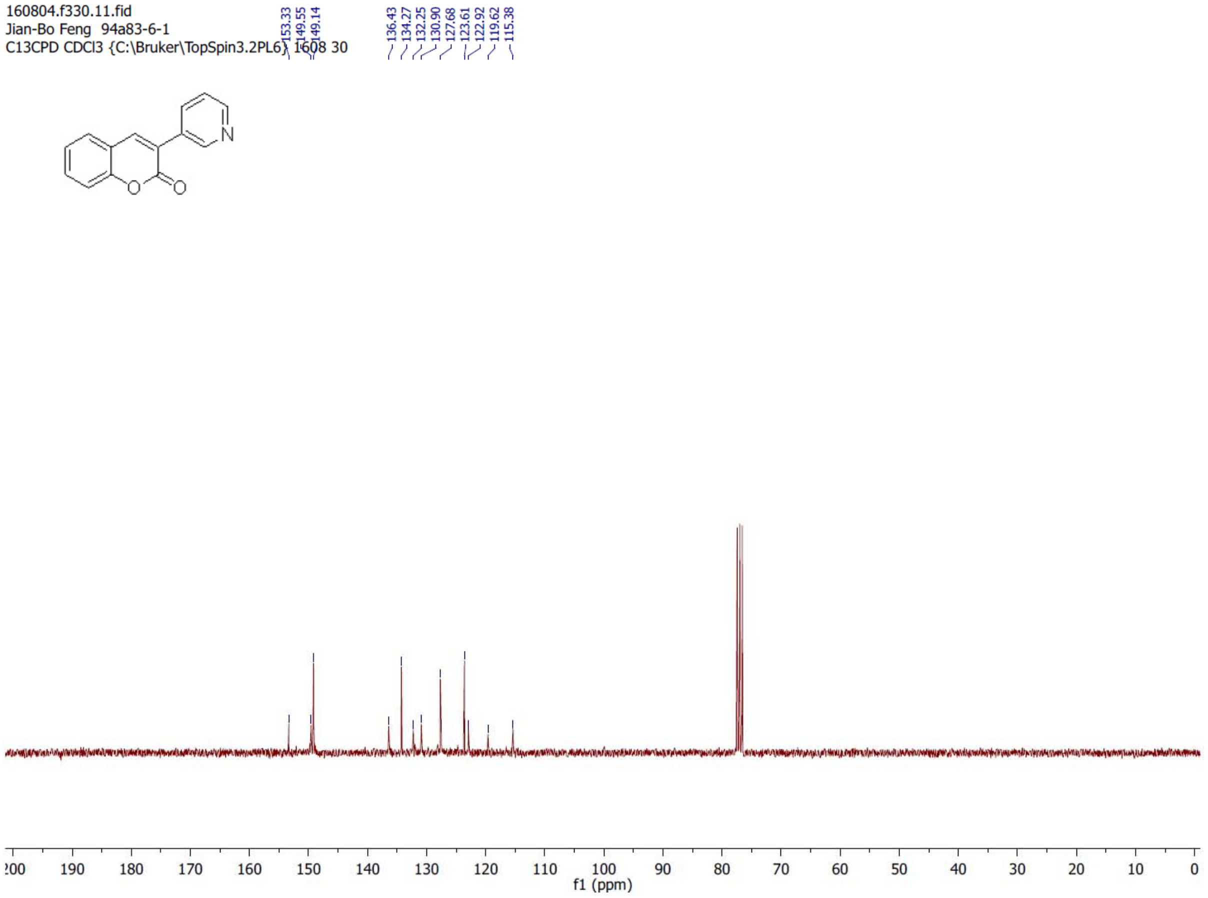

160804.f330.11.fid
Jian-Bo Feng 94a83-6-1
C13CPD CDCl3 {C:\Bruker\TopSpin3.2PL6} 1608 30
153.33 149.55 149.14
136.43 134.27 132.25 130.90 127.68 123.61 122.92 119.62 115.38
N
O
O
200 190 180 170 160 150 140 130 120 110 100 90 80 70 60 50 40 30 20 10 0
f1 (ppm)

160803.f318.10.fid
Jian-Bo Feng 94a80-1-1
PROTON CDCl3 {C:\Bruker\TopSpin3.2PL6} 1608 18

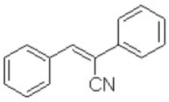


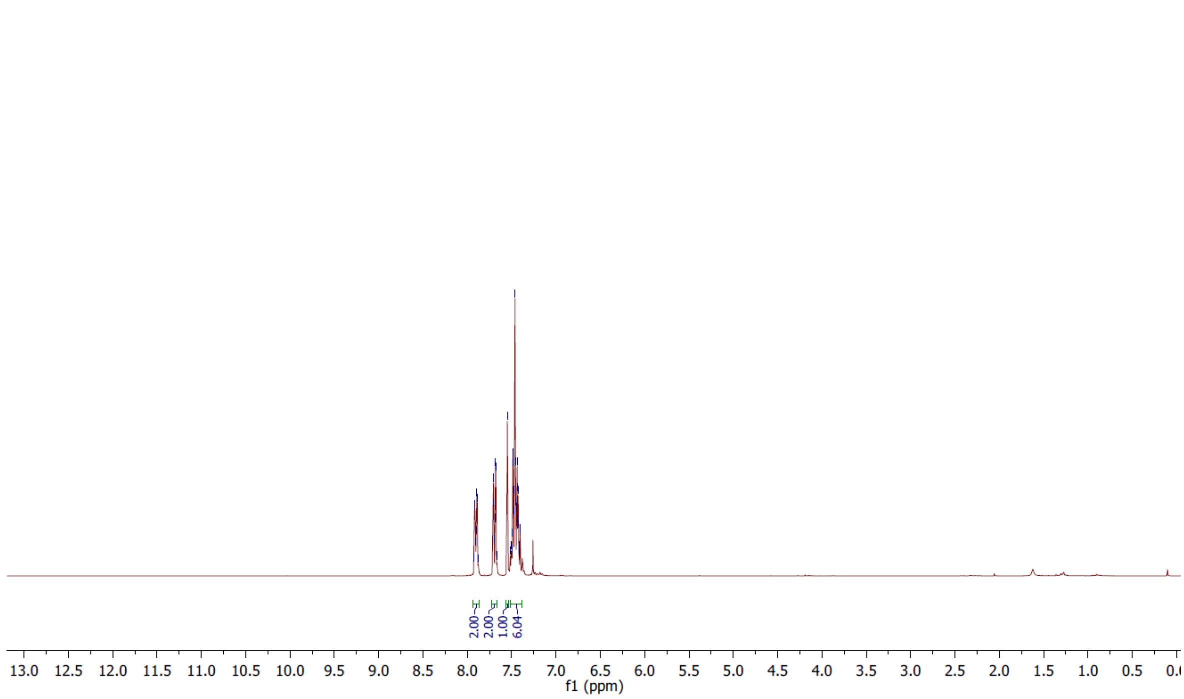


160803.f318.11.fid
Jian-Bo Feng 94a80-1-1
C13CPD CDCl3 {C:\Bruker\TopSpin3.2PL6} 1608 18

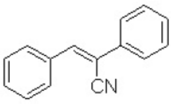


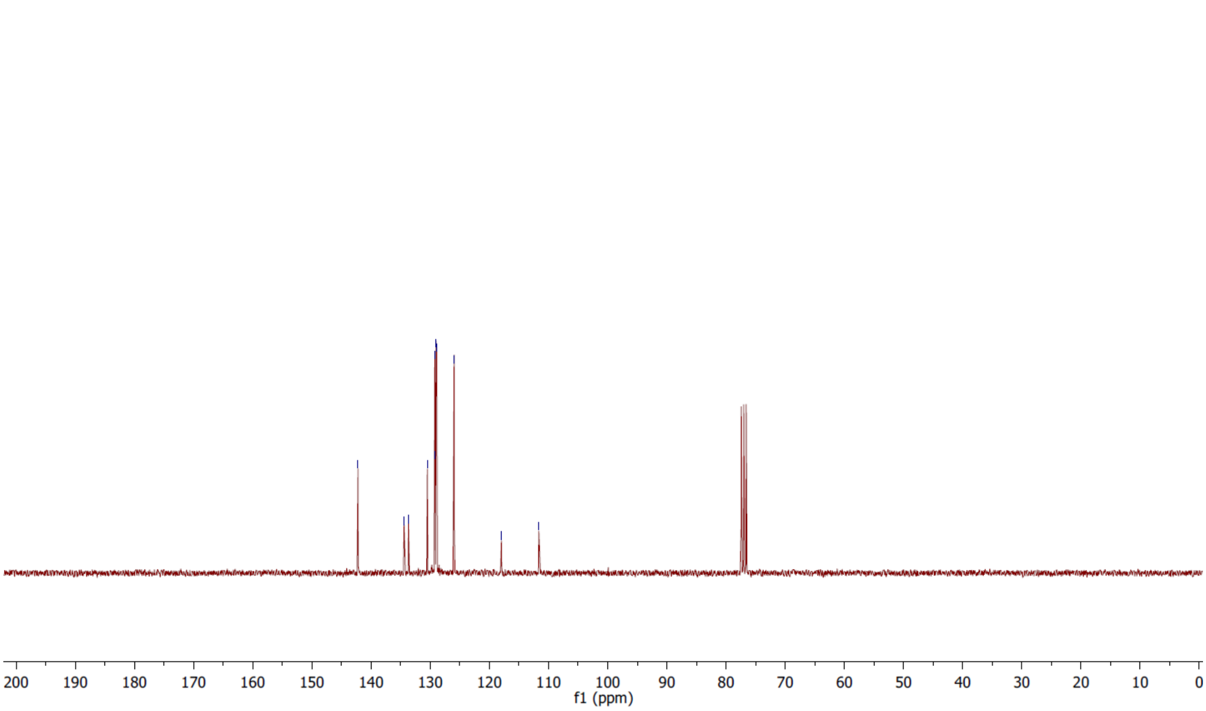

160808.303.10.fid
Jian-Bo Feng 94a83-2
Au1H DMSO /opt/topspin 1608 3

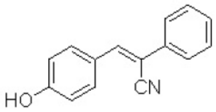

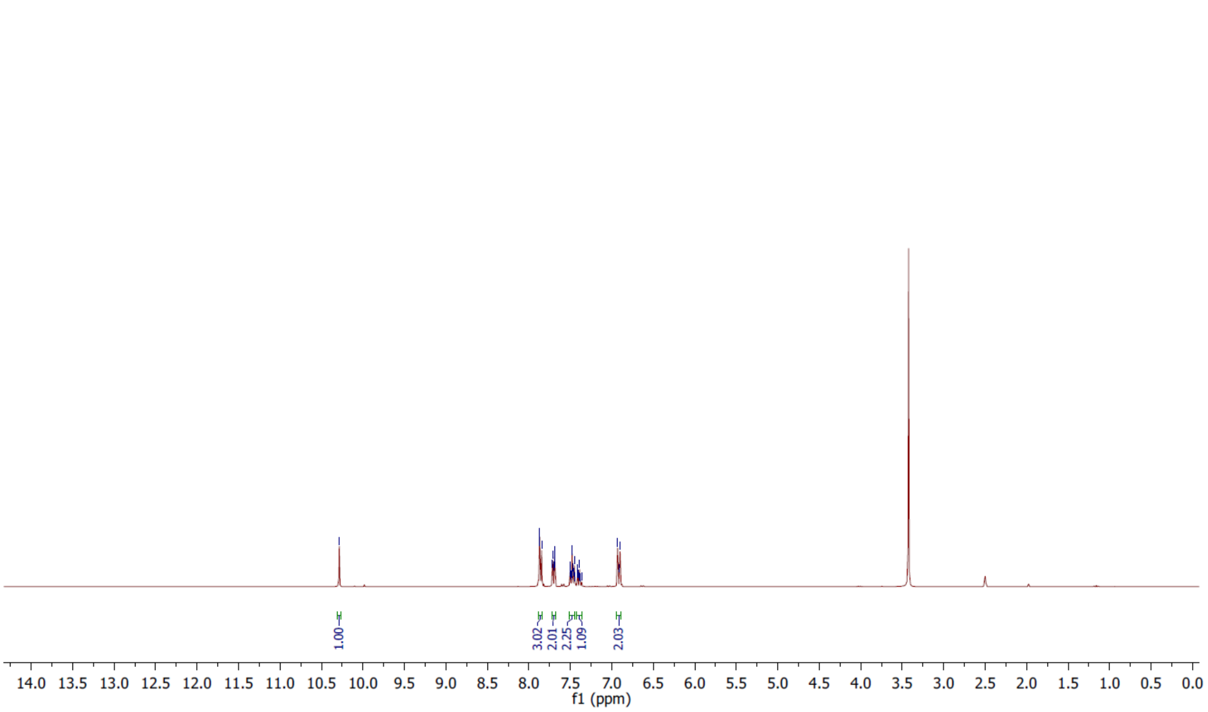


160808.303.11.fid
Jian-Bo Feng 94a83-2
Au13C DMSO /opt/topspin 1608 3

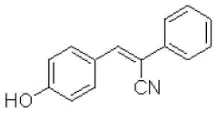

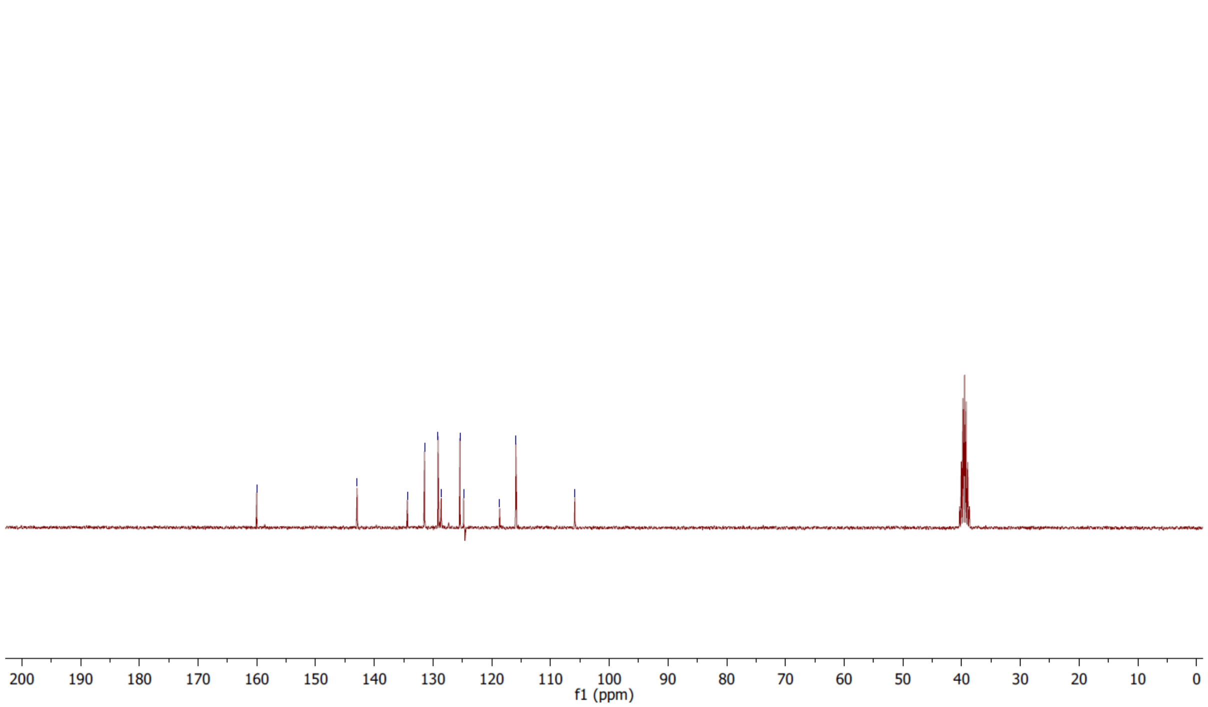

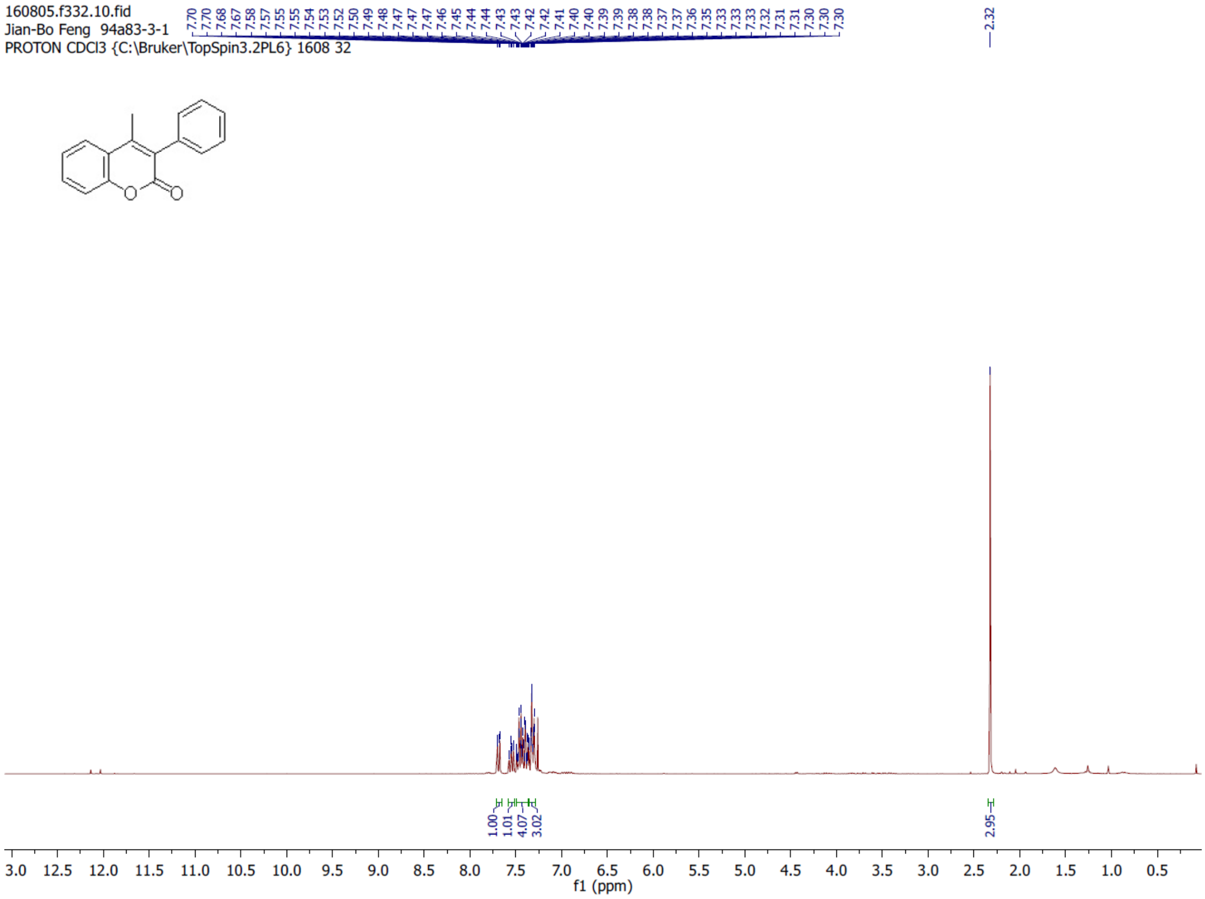
160805.f332.10.fid
Jian-Bo Feng 94a83-3-1
PROTON CDCl3 {C:\Bruker\TopSpin3.2PL6} 1608 32
7.70 7.70 7.68 7.67 7.58 7.57 7.55 7.55 7.54 7.53 7.52 7.50 7.49 7.48 7.47 7.47 7.47 7.46 7.45 7.44 7.44 7.43 7.43 7.42 7.42 7.41 7.40 7.40 7.39 7.39 7.38 7.38 7.37 7.37 7.36 7.35 7.33 7.33 7.33 7.32 7.31 7.31 7.30 7.30 7.30
2.32
1.00
1.01
4.07
3.02
2.95
f1 (ppm)

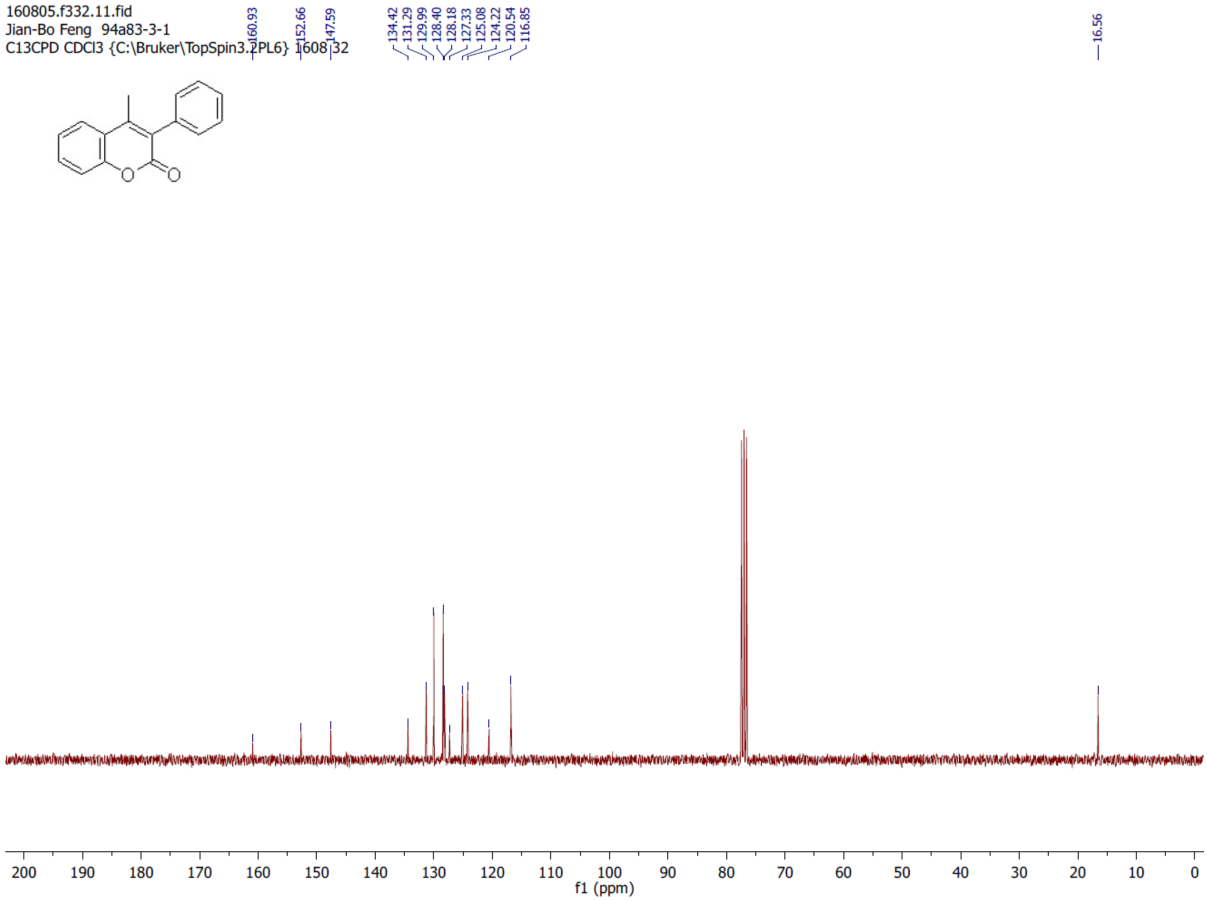
160805.f332.11.fid
Jian-Bo Feng 94a83-3-1
C13CPD CDCl3 {C:\Bruker\TopSpin3.2PL6} 1608 32
160.93
152.66
147.59
134.42 131.29 129.99 128.40 128.18 127.33 125.08 124.22 120.54 116.85
16.56
f1 (ppm)
